# Structure–Activity Relationships of Triple-Action Platinum(IV) Prodrugs with Albumin-Binding Properties and Immunomodulating Ligands

**DOI:** 10.1021/acs.jmedchem.1c00770

**Published:** 2021-08-17

**Authors:** Philipp Fronik, Isabella Poetsch, Alexander Kastner, Theresa Mendrina, Sonja Hager, Katharina Hohenwallner, Hemma Schueffl, Dietmar Herndler-Brandstetter, Gunda Koellensperger, Evelyn Rampler, Joanna Kopecka, Chiara Riganti, Walter Berger, Bernhard K. Keppler, Petra Heffeter, Christian R. Kowol

**Affiliations:** † Faculty of Chemistry, Institute of Inorganic Chemistry, University of Vienna, Waehringer Strasse 42, 1090 Vienna, Austria; ‡ Institute of Cancer Research and Comprehensive Cancer Center, Medical University of Vienna, Borschkegasse 8a, 1090 Vienna, Austria; § Research Cluster “Translational Cancer Therapy Research”, 1090 Vienna, Austria; ∥ Faculty of Chemistry, Institute of Analytical Chemistry, University of Vienna, Waehringer Strasse 38, 1090 Vienna, Austria; ⊥ Department of Oncology, University of Torino, via Santena 5/bis, 10126 Torino, Italy

## Abstract

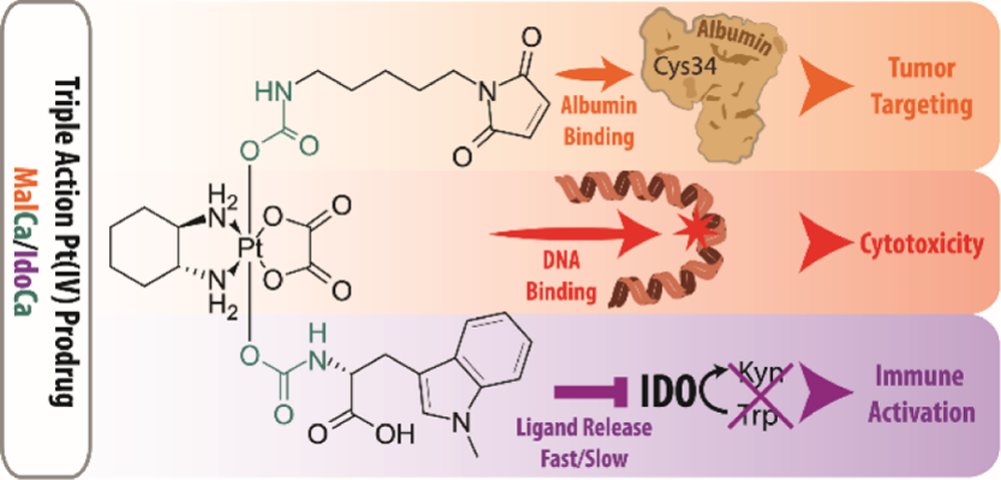

Chemotherapy with platinum complexes is essential for clinical anticancer therapy. However, due to side effects and drug resistance, further drug improvement is urgently needed. Herein, we report on triple-action platinum(IV) prodrugs, which, in addition to tumor targeting *via* maleimide-mediated albumin binding, release the immunomodulatory ligand 1-methyl-d-tryptophan (1-MDT). Unexpectedly, structure–activity relationship analysis showed that the mode of 1-MDT conjugation distinctly impacts the reducibility and thus activation of the prodrugs. This in turn affected ligand release, pharmacokinetic properties, efficiency of immunomodulation, and the anticancer activity *in vitro* and in a mouse model *in vivo*. Moreover, we could demonstrate that the design of albumin-targeted multi-modal prodrugs using platinum(IV) is a promising strategy to enhance the cellular uptake of bioactive ligands with low cell permeability (1-MDT) and to improve their selective delivery into the malignant tissue. This will allow tumor-specific anticancer therapy supported by a favorably tuned immune microenvironment.

## Introduction

The antitumor activity of cisplatin was already discovered in the 1960s, resulting in its approval in 1978.^1,2^ Subsequently, two additional platinum(II) complexes, carboplatin and oxaliplatin, have been approved worldwide.^3^ This compound class is still widely used as a first-line treatment in many therapeutic schemes and has more recently caused another surge of interest, due to its synergism with immune checkpoint inhibitors like pembrolizumab.^3^ Moreover, it is already widely accepted that oxaliplatin requires an intact immune system to fully unfold its anticancer activity.^3^ However, the low selectivity of platinum drugs for the malignant tissue is still a major limitation as treatment with these compounds frequently results in severe side effects.^4^ Additionally, intrinsic or acquired resistance, often based on reduced drug accumulation in the cancer tissue, represents another major drawback.^5^ Consequently, drug combination strategies, exploiting the specific cancer biology, are of high interest to improve the efficacy of therapy in tandem with reduced side effects.

Cancer cells are known to actively inhibit immune recognition through diverse mechanisms, including loss of the antigen-presenting machinery or expression of inhibitory molecules and enzymes that induce T-cell suppression.^6^ One specific enzyme is indoleamine 2,3-dioxygenase (IDO), which catabolizes the amino acid tryptophan (Trp) to kynurenine (Kyn). Binding of Kyn to the aryl hydrocarbon receptor inhibits T-cell activation and supports regulatory T-cell proliferation.^7^ IDO expression has been described for several tumor types and identified as a major mechanism supporting immune evasion of cancer cells. The interest in this enzyme is also reflected by the clinical development of several IDO inhibitors, such as 1-methyltryptophan (1-MT).^8^ In numerous preclinical studies, 1-MT has been investigated, both as pure stereoisomers as well as racemic mixtures.^9−11^ Interestingly, even though the l-isomer [1-methyl-l-tryptophan (1-MLT)] inhibits IDO more efficiently in cell culture, the d-isomer [1-methyl-d-tryptophan (1-MDT); indoximod] has emerged as preferential compound for clinical development, due to its higher immunogenic anticancer activity *in vivo*.^10^ With regard to platinum drugs, there is strong evidence that the combination with IDO inhibition (*e.g.*, by 1-MDT) is highly synergistic.^12−15^ There are already some promising reports on nano-formulations combining platinum-based chemotherapy with IDO inhibitors.^13,14,16^ However, such nano-formulations exhibit various major limitations for clinical use including challenging preparation procedures, difficulties in batch-to-batch reproducibility, stability/shelf-life, and varying loading efficiency.^17^ Therefore, other more stable and controllable approaches are of interest, such as platinum(IV) prodrugs. Platinum(IV) complexes are promising tools to design multi-modulatory drugs as they are not only kinetically more inert than their platinum(II) counterparts but also offer the ability to attach additional ligands.^18,19^ Thus, upon reduction, platinum(IV) complexes release both, the cytotoxic platinum(II) species and the bioactive compound(s). In this way, simultaneous, tumor-specific release of two or more drugs is possible, which can target the cancer cells by different and ideally synergistic modes of actions.^20^ Moreover, this strategy is especially interesting for ligands which are characterized by a low cell penetration. The clinically investigated IDO inhibitor 1-MDT is a zwitterionic amino acid under physiological pH conditions, which hampers its cellular uptake. Consequently, usually very high levels of 1-MDT have to be applied for sufficient activity. Formation of a platinum(IV) prodrug with intracellular release of 1-MDT is an elegant way to modulate the characteristics of this IDO inhibitor. Recently, the first prodrug approach using cisplatin-releasing complexes and an albumin-targeting prodrug strategy has been reported by Awuah *et al.*(16)

Binding to serum albumin represents one of the most efficient strategies to target the tumor tissue.^21,22^ Due to the fact that albumin serves as a transporter for several nutrients in the blood stream, fast-growing tumor cells are characterized by an increased uptake of this plasma protein and additionally use albumin as an amino acid source.^23^ Furthermore, the enhanced permeability and retention effect, that is based on the combination of leaky blood vessels together with impaired lymph drainage, contributes to albumin accumulation in the malignant tissue.^24,25^ The potential of targeting cancer cells by their enhanced need for albumin has been demonstrated by nab (nanoparticle albumin-bound) paclitaxel (ABRAXANE), which is already approved for treatment of various cancer types.^26,27^ In addition, aldoxorubicin, a compound which successfully finished a phase III clinical trial (study number NCT02049905) in soft tissue sarcoma, is of note. This compound utilizes a maleimide moiety, which specifically targets the single free cysteine residue of albumin at position 34.^28^ We have recently reported on the first maleimide-bearing platinum(IV) complexes.^29,30^ Here, especially, the oxaliplatin derivatives not only showed excellent reduction properties and tumor accumulation but also promising antitumor activity *in vivo*.^29^ Noteworthy, our studies revealed that for the success of an albumin-targeted prodrug strategy, very stable platinum(IV) complexes are necessary. Platinum(IV) complexes with a cisplatin core faced much faster reduction kinetics (compared to oxaliplatin or carboplatin derivatives)^31,32^ and are therefore less ideal for long-circulating drug delivery systems like albumin-conjugated prodrugs.^29^ Indeed, in the study by Awuah *et al.*, the IDO-releasing cisplatin drug faced several problems. The authors used a very lipophilic C_16_–alkyl chain for non-covalent albumin binding and, consequently, the compound was hardly soluble and had to be encapsulated into polylactide-*co*-glycolide–polyethylene glycol polymers to achieve sufficient solubility. Despite their efforts, the *in vivo* plasma half-life of the drug was only 1 h, indicating that the effect of the albumin nano-carrier did not apply. In order to successfully develop tumor-specific 1-MDT-releasing prodrugs, new complexes with enhanced stability and albumin-binding properties are required to achieve high tumor accumulation and anticancer activity. Based on their high reduction stability, oxaliplatin-releasing compounds are ideal candidates for this approach. Moreover, oxaliplatin is known for its strong immunogenic activity and promising synergistic activity with IDO inhibitors.^13,33,34^

In the present study, we designed the first oxaliplatin-based albumin-targeted platinum(IV) complexes with an 1-MDT ligand. For endogenous albumin targeting, ideally maleimides are used because of their exceptionally fast binding rates in serum and sufficient solubility for *in vivo* studies. Other moieties usually suffer either from very low solubility (long aliphatic alkyl chains^16^) or too slow binding kinetics (*e.g.*, carbonylacrylic reagents^35^). In order to generate a lead candidate for further preclinical investigations, several 1-MDT-bearing derivatives with different maleimide linkage types were synthesized to investigate chemical and pharmacological drug properties. As maleimide compounds are difficult to test in cell culture due to their proneness to hydrolysis and their reactivity with cell medium components, succinimide derivatives were additionally synthesized for *in vitro* analysis.

## Results and Discussion

### Synthesis

**Scheme 1:**
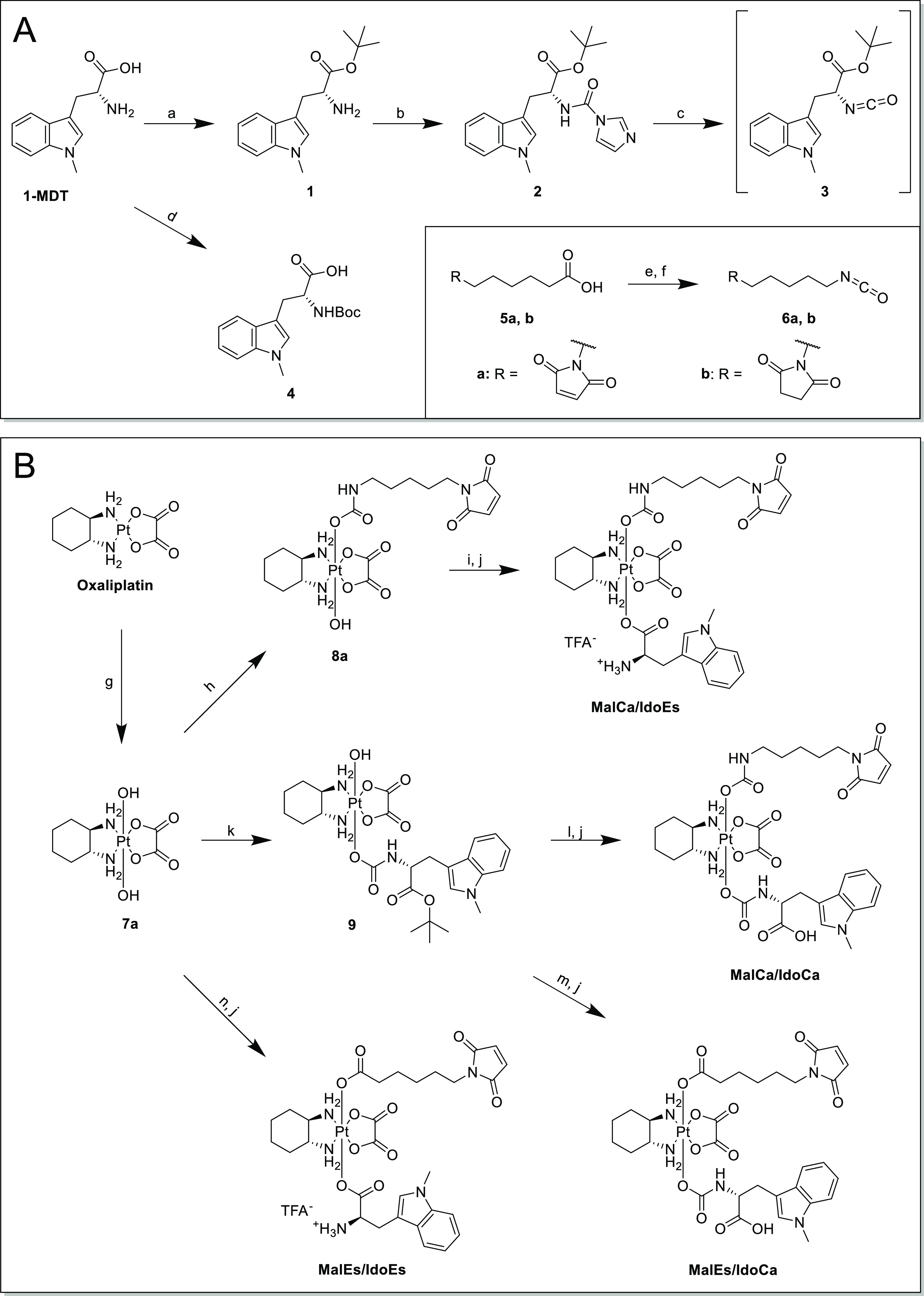
(A) Synthetic Routes for 1-MDT and Maleimide Building Blocks; (a) 60% HClO_4_, *t*-Butyl Acetate, 0 °C–Room Temperature (RT); (b) 1,1′-Carbonyldiimidazole, *N*,*N*-Diisopropylethylamine (DIPEA), Dichloromethane (DCM), RT; (c) Dimethyl Sulfoxide (DMSO), 80 °C; (d) Di-*t*-butyl Dicarbonate, NaOH, Dioxane/Water 2:1, RT; (e) Diphenylphosphoryl Azide (DPPA), Triethylamine (TEA), Toluene; and (f) Toluene, 100 °C; (B) Synthetic Routes for the Final Maleimide–Platinum(IV) 1-MDT Complexes; (g) H_2_O_2_ (50% w/w), H_2_O, RT; (h) **6a**, DMSO, RT; (i) **4**, TBTU, TEA, Dimethylformamide (DMF), RT; (j) 10% TFA in DCM, RT; (k) **3**, DMSO, RT; (l) **6a**, DMF, RT; (m) **5**, *N*-Ethyl Maleimide, TBTU, TEA, DMF, RT; and (n) **4**, **6a**, TBTU, TEA, DMF, RT

It is known that the exact ligand coordination and inner sphere of a platinum(IV) core are substantial for its reduction behavior.^36,37^ Therefore, we decided to elucidate the detailed structure–activity relationships for the platinum(IV) prodrugs by synthesizing four distinctly different complexes based on the axial ligand-binding motifs of the maleimide and IDO inhibitor. 1-MDT is an amino acid that possesses two functional groups, which are potentially suitable for coupling to the platinum core: the carboxylic acid to form a carboxylate (in this manuscript denoted as “ester”) and the primary amine with formation of a carbamate. Notably, so far only the “straightforward” coupling *via* the carboxylic acid has been reported.^16^ We successfully established the ester coupling to the oxaliplatin core using 2-(1*H*-benzotriazole-1-yl)-1,1,3,3-tetramethylaminium tetrafluoroborate (TBTU) and the carbamate formation *via* isocyanates (Scheme 1). To obtain the building blocks for further synthesis, on the one hand, the carboxylic acid moiety of 1-MDT was protected *via t*-butyl ester (**1**) before converting the amine to isocyanate (**3**) using 1,1′-carbonyldiimidazole (Scheme 1A). On the other hand, Boc-protection of the primary amine resulted in the ester-building block **4**. Maleimide moieties were similarly coupled either as a commercially available carboxylic acid (**5a**) or as an isocyanate (**6a**) (Scheme 1A). The oxaliplatin precursors were obtained by oxidation with H_2_O_2_ in water yielding the dihydroxido complex (**7a**) or in acetic acid with formation of the mono-acetato complex (**7b**).^38,39^ Subsequent reactions of **7a** with either isocyanate building block yielded intermediates **8a** or **9** (Scheme 1B). To generate the final complex **MalCa/IdoCa** [**mal**eimide *via***ca**rbamate, 1-MDT as an IDO inhibitor (**Ido**) *via***ca**rbamate], complex **9** reacted further with maleimido-isocyanate **6a**. For **MalEs/IdoCa** [**mal**eimide *via***es**ter, 1-MDT as an IDO inhibitor (**Ido**) *via***ca**rbamate] and **MalCa/IdoEs** [**mal**eimide *via***ca**rbamate, 1-MDT as an IDO inhibitor (**Ido**) *via***es**ter], the respective carbamato-intermediate **8a** or **9** reacted with the other respective carboxylic acids *via* TBTU-mediated coupling (in case of **8a**, *N*-ethyl maleimide was added to the reaction mixture in order to prevent adduct formation of the TBTU side product with the maleimide of **8a**). With regard to **MalEs/IdoEs** [**mal**eimide *via***es**ter, 1-MDT as an IDO inhibitor (**Ido**) *via***es**ter], the coupling of both ligands was performed using a one-pot procedure. In all cases, subsequent deprotection with trifluoroacetic acid (TFA) yielded the final products after preparative high-performance liquid chromatography (HPLC) purification.

**Figure 1:**
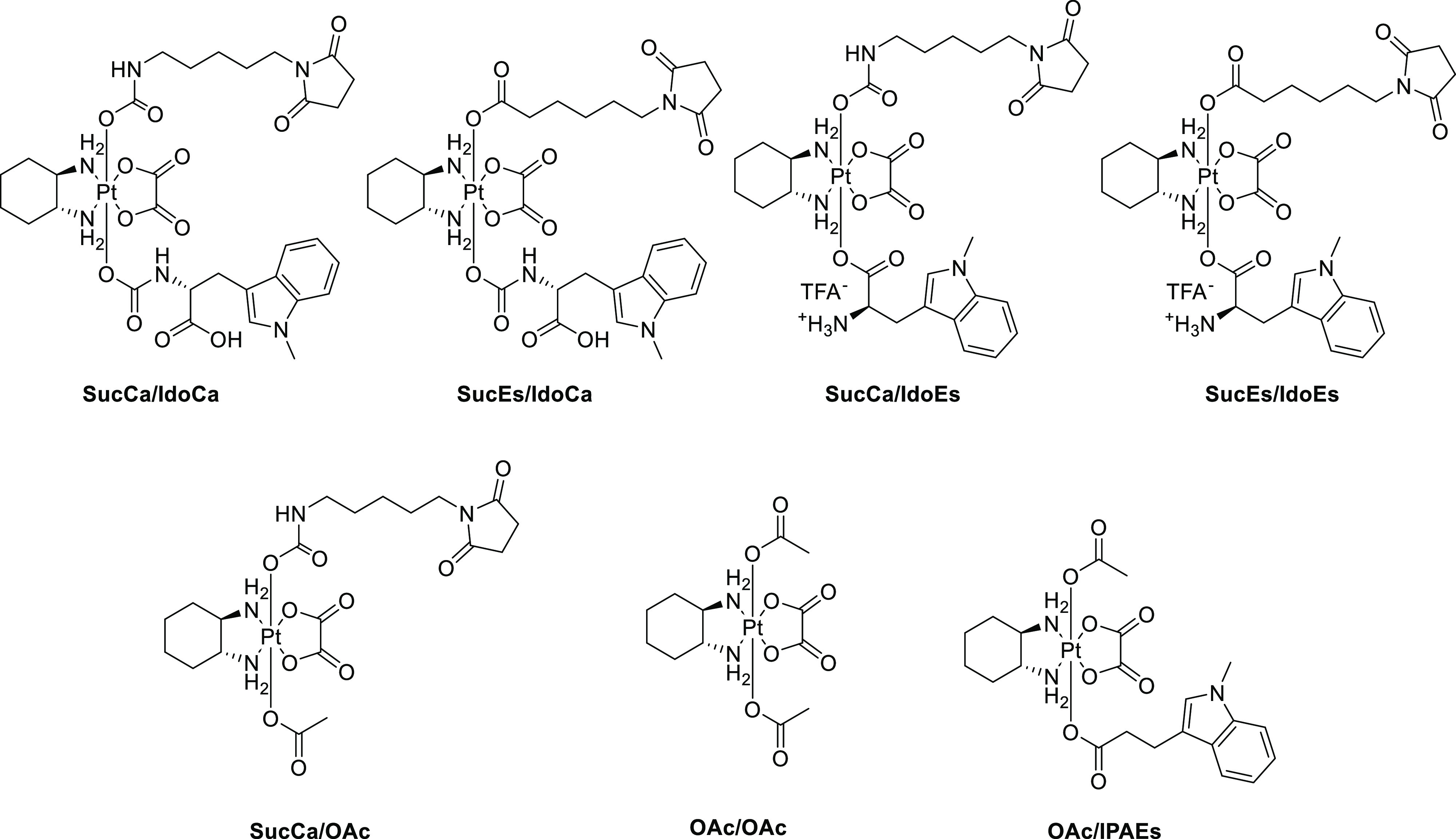
Structures of platinum(IV) reference compounds for cell culture investigations.

In parallel, the respective succinimides of all four complexes (**SucCa/IdoCa**, **SucCa/IdoEs**, **SucEs/IdoCa**, and **SucEs/IdoEs**, Figure 1) were synthesized in an identical manner using the succinimide precursors (**5b** and **6b**). In addition, the acetato-derivative **SucCa/OAc** was prepared from the hydroxido/acetato platinum(IV) precursor **7b** using isocyanate **6b** (see the experimental section part).

### Albumin Binding and Serum Stability

**Figure 2:**
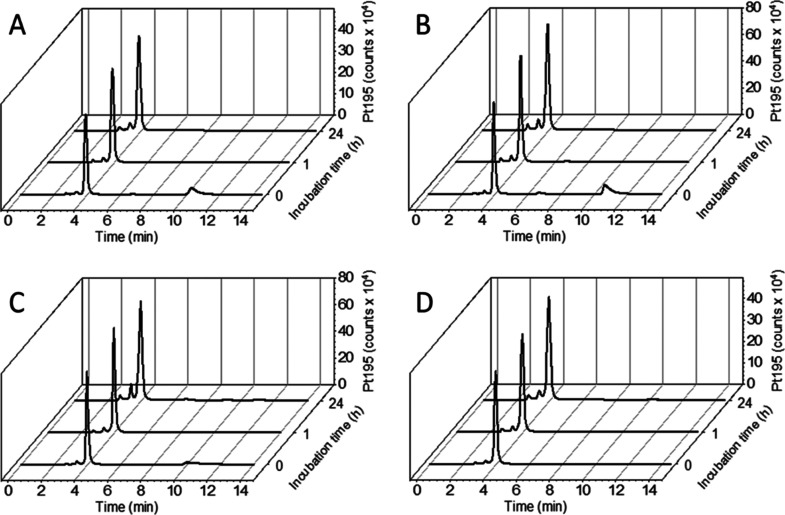
Platinum-SEC–ICP–MS traces of the complexes (A) **MalEs/IdoCa**, (B) **MalCa/IdoCa**, (C) **MalCa/IdoEs**, and (D) **MalEs/IdoEs** after incubation in FCS at 37 °C after 0, 1, and 24 h. The small peaks at ∼11 min retention time indicate the initial low-molecular-weight complexes; the peaks at ∼4 min indicate the albumin-bound platinum.

As a first step, we investigated the albumin-binding kinetics and serum stability of the novel maleimide-bearing drugs by size exclusion chromatography followed by inductively coupled plasma mass spectrometry (SEC–ICP–MS) after incubation in fetal calf serum (FCS, buffered with 150 mM phosphate buffer to ensure a stable pH over 24 h and 1% DMF for sufficient solubility) at 37 °C (Figure 2). Already at *t* = 0, most of the platinum was bound to the albumin fraction with a retention time of ∼4 min, and only small amounts were present in the low-molecular-weight region at around 10–12 min (a chromatogram of the sulfur trace of pure serum can be found in Figure S1). After 1 h, no low-molecular-weight platinum species could be detected in either of the samples (Figure 2). This confirms very fast albumin-binding properties of all four derivatives. Interestingly, derivatives with 1-MDT coupled *via* an ester to the platinum(IV) core conjugated slightly faster to albumin than the derivatives with an 1-MDT carbamate bond to the metal. Moreover, the results also suggest that Michael addition to the thiol proceeds substantially quicker than hydrolysis of the maleimide, which would generate a species in the low-molecular-weight region unable to bind to albumin. The data also clearly indicate high stability of all complexes in serum without significant degradation of the platinum(IV) albumin adduct within 24 h. As reference measurements, also the succinimide analogues were investigated by SEC–ICP–MS in serum (Figure S2). No significant binding to albumin or other proteins could be observed, proving that the maleimide is indeed the crucial moiety for coupling.

### Reduction Properties

**Figure 3:**
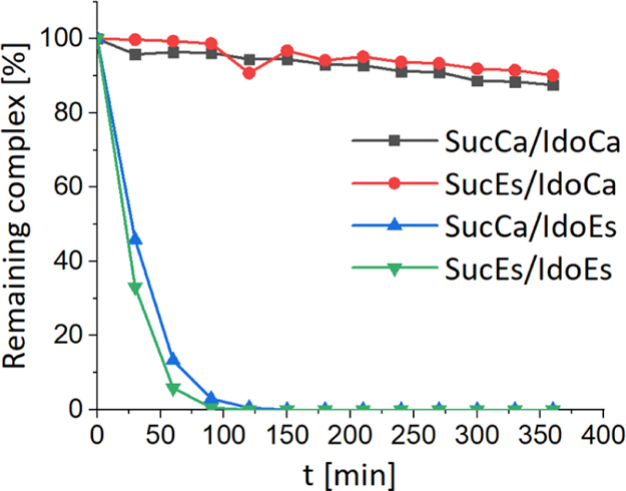
Reduction kinetics measured by UHPLC of succinimide complexes (1 mM) at 20 °C with a 10-fold excess of AA in phosphate buffer (500 mM, pH 7.4) containing 1% DMF.

As already mentioned, one of the most crucial parameters in the biological activity of platinum(IV) drugs is their ability to be reduced in tumor tissue. Recently, we have demonstrated that in case of maleimide-bearing complexes,^29^ slow-reducing platinum(IV) derivatives are characterized by a longer plasma half-life time, improved tumor accumulation, and superior anticancer activity *in vivo*. However, the compounds should not be too stable as the platinum(IV) prodrugs need to be activated *via* reduction in the specific microenvironment of the tumor in order to exert their antitumor potential.^36^ Due to the hydrolysis of the maleimide moiety, we analyzed the reduction properties of the respective succinimide complexes by ultra-HPLC (UHPLC) after incubation in phosphate buffer at pH 7.4 (including 1% DMF) with a 10-fold excess of l-ascorbic acid (AA) as a reducing agent. Notably, huge differences between the platinum complexes could be observed. Unexpectedly, both complexes with ester-like 1-MDT conjugations (**SucEs/IdoEs** and **SucCa/IdoEs**) were completely reduced within 2 h (Figure 3). In comparison, the carbamate analogues **SucEs/IdoCa** and **SucCa/IdoCa** displayed the expected very high stability in the reductive environment with 90% of the complex being intact after 6 h (Figure 3). This slow reduction behavior is in line with several literature reports on other oxaliplatin(IV) complexes.^29,40−42^ In contrast, the binding mode of the maleimide moiety did not substantially influence the reduction properties of these complexes. However, the question remained how this dramatic divergence in the reduction kinetics, just by changing the coordination mode of 1-MDT from an ester to a carbamate, can be explained. The main difference is that in case of **SucEs/IdoEs** and **SucCa/IdoEs** the amino group of 1-MDT is free, whereas in case of **SucEs/IdoCa** and **SucCa/IdoCa**, a free carboxylic acid is present. Therefore, it can be concluded that the very fast reduction is associated with the free amino group of the ester-bound complexes. To investigate this hypothesis, an analogue using 1-methyl-indole-3-propanoic acid lacking the amino group was synthesized (**OAc/IPAEs**; Figure 1). Subsequently, incubation studies with AA indeed revealed comparable reduction kinetics to the carbamate-linked complexes (Figure S3A). Therefore, it can be assumed that the electron transfer is facilitated by an interaction/stabilization of the reducing agent with the amine. This phenomenon is not specific for AA but was also visible when using dl-dithiothreitol as a reductant. Again, distinctly slower reduction kinetics were observed for **OAc/IPAEs** in comparison to **SucCa/IdoEs** (Figure S4).

### Impact of the Different Reduction Kinetics on the Anticancer Activity of the Succinimide Prodrugs

To evaluate whether the observed differences in the reduction kinetics also influence the biological activities of the compounds, the cytotoxicity of the new drugs was determined in cell culture after 48 and 72 h. Since oxaliplatin is clinically used against gastrointestinal cancers, two colon carcinoma cell lines, one of human (HCT116) and one of murine origin (CT26), were analyzed for this purpose (Table 1 and Table S1). In general, we observed a similar structure activity pattern in both models. In more detail, all platinum(IV) drugs exhibited higher IC_50_ values than free oxaliplatin. This was not unexpected as based on the prodrug nature of these compounds, they need additional time to be activated and to release their active platinum(II) species. Accordingly, the derivatives with ester-like 1-MDT conjugations (**SucEs/IdoEs** and **SucCa/IdoEs**), which were characterized by faster reduction, were more active than the 1-MDT carbamate-linked analogues (**SucEs/IdoCa** and **SucCa/IdoCa**) with higher reduction stability. In detail, after 48 h, the 1-MDT carbamate-linked complexes did not show any anticancer activity up to the highest concentration of 100 μM in both cell lines. In contrast, the 1-MDT ester-linked complexes had IC_50_ values of ∼60–90 μM in CT26 cells and ∼25 μM in HCT-116 cells after 48 h. The same trends were also visible after 72 h, although all compounds were more active than after 48 h (Table S1). The coordination of the succinimide moiety *via* ester or a carbamate only minimally influenced the antiproliferative activity. There was also a distinct difference between the two platinum(IV) reference drugs, with **OAc/OAc** being at least 3-fold more active than **SucCa/OAc**. Noteworthy, the ratio between the activity of oxaliplatin and the platinum(IV) complexes became distinctly smaller upon longer incubation times (*e.g.*, in case of **SucEs/IdoEs** in CT26 cells from a factor of 61 to a factor of 24). This again indicates that prolonged incubation provides sufficient time for more complete reduction and, thus, oxaliplatin release. To further investigate this hypothesis, the activity of the compounds was tested in the presence of 5-fold excess AA as an reducing agent (Figure S5). While the addition of AA did not have any effect on the cytotoxic properties of oxaliplatin, the anticancer activity of our new compounds was considerably enhanced after 48 h. This effect was reduced, when we increased the incubation time of the cytotoxicity assays from 48 to 72 h. Here, especially in case of HCT116 cells (Table S1), addition of AA had basically no additional effect on the two fast reducing compounds, indicating that in this time frame, full reduction of the platinum(IV) was already achieved without additional AA. With regard to the reference drugs, **SucCa/OAc**, lacking the free amino group, behaved as anticipated, similar to the carbamate-linked complexes. In contrast, **OAc/OAc** could not be further activated by addition of AA. This is unexpected as the reduction kinetics of these two compounds were comparable (Figure S3B). To evaluate whether reduction leads to the release of a functional platinum(II) species from our prodrugs, we investigated the induction of two main hallmarks of oxaliplatin activity upon addition of AA: DNA damage indicated by phosphorylation of histone H2A.X at position Ser139 and cell cycle arrest in G2/M due to activation of the DNA damage sensor P53.^43^ As shown in Figures S6 and S7, reduction by AA resulted in both, increased pH2A.X signals and an increased fraction of cells in the G2/M phase of the cell cycle similar to oxaliplatin, especially in case of the fast reducing agents **SucEs/IdoEs** and **SucCa/IdoEs**.

**Table 1:** Cytotoxicity Determined by MTT Assay in Murine CT26 and Human HCT116 Colon Cancer Cells after 48 h Incubation with and without 5 equiv AA^a^

		+AA	
	mean ± SD	mean ± SD	ratio
CT26—IC_50_ Values (μM)—48 h			
oxaliplatin	1.50 ± 0.39	1.53 ± 0.25	0.98
**OAc/OAc**	25.66 ± 1.40	22.60 ± 0.16	1.14
**SucCa/OAc**	75.21 ± 0.11	28.17 ± 1.81	2.67
**SucEs/IdoCa**	>100	26.65 ± 0.75	≥3
**SucCa/IdoCa**	>100	38.84 ± 3.84	≥2.6
**SucEs/IdoEs**	91.51 ± 3.84	11.15 ± 0.37	8.21
**SucCa/IdoEs**	60.09 ± 6.49	10.81 ± 1.04	5.56
HCT116—IC_50_ Values (μM)—48 h			
oxaliplatin	0.72 ± 0.01	0.67 ± 0.01	1.07
**OAc/OAc**	26.56 ± 2.41	20.10 ± 1.57	1.32
**SucCa/OAc**	>100	33.96 ± 1.58	≥2.9
**SucEs/IdoCa**	>100	35.39 ± 6.54	≥2.8
**SucCa/IdoCa**	>100	43.27 ± 2.10	≥2.3
**SucEs/IdoEs**	22.80 ± 1.15	6.94 ± 0.33	3.28
**SucCa/IdoEs**	26.00 ± 3.21	7.36 ± 1.14	3.53

a Values are given as mean ± standard deviation (SD).

Finally, the effects of our prodrugs on healthy tissue were tested in non-malignant cell lines (Table S2). As kidney and liver tissue are typically strongly affected by therapy with platinum compounds, we chose human embryonic kidney cells (HEK293) and human hepatic (WRL68) cells for these experiments. The experiments revealed that while both cell lines were very sensitive to oxaliplatin treatment in the low μM range, the prodrugs were up to 40-fold less toxic, suggesting a low side-effect profile of our compounds.

**Figure 4:**
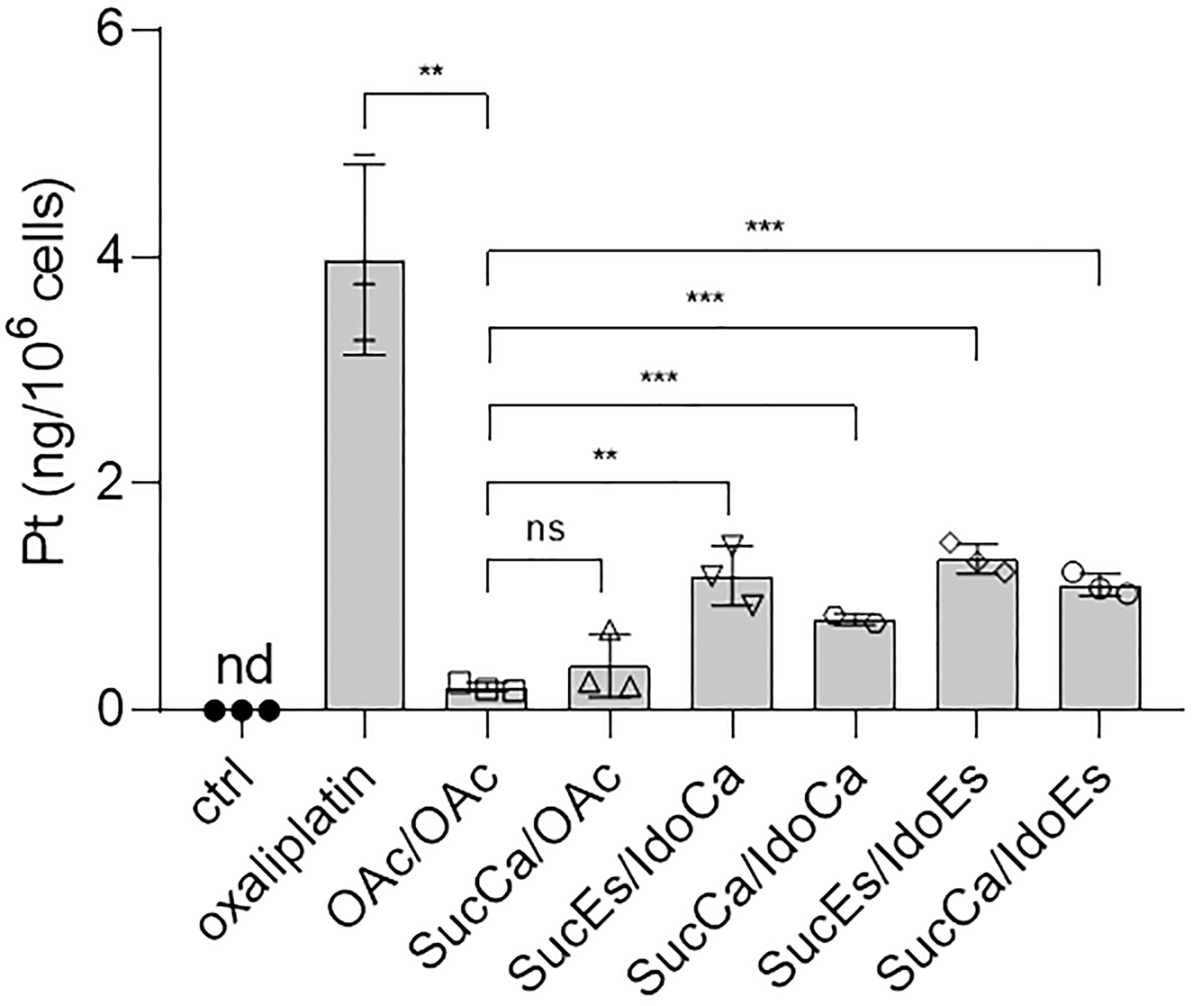
Cellular platinum levels in HCT116 cells after 3 h incubation with 20 μM of the compounds measured by ICP–MS. Blanks (treated wells without cells) were subtracted from each value. Bars indicate mean ± SD of triplicates. Significance was calculated compared to the **OAc/OAc** mean value using an unpaired *t*-test (two-tailed).

In order to rule out that the observed effects in the viability assays were based on differences in the uptake between fast- and slow-reducing prodrugs, we investigated the intracellular platinum levels after 3 h incubation in HCT116 cells. Several interesting observations were made (Figure 4): (1) The platinum(II) complex, oxaliplatin, was taken up by the cells much more efficiently than any of the platinum(IV) prodrugs. (2) Compounds that carry a 1-MDT ligand are taken up at higher levels than the platinum(IV) reference compounds **OAc/OAc** and **SucCa/OAc**. (3) There was no significant difference in the platinum levels of cells treated with different 1-MDT-bearing complexes. Lipophilicity is a parameter known to impact on the cellular drug accumulation and, thus, cytotoxicity. Hence, compounds with higher lipophilicity usually have higher cellular uptake and cytotoxicity. In order to assess this criterion, we determined the log *D*_7.4_ value for oxaliplatin, **OAc/OAc**, and the four 1-MDT-bearing prodrugs using the shake flask method, and platinum concentrations were measured by ICP–MS analysis (Table 2). Log *D*_7.4_ was chosen due to the fact that our platinum(IV) prodrugs possess charged moieties (NH_3_^+^ or COO^–^) at physiological pH, and consequently, a direct comparison is only meaningful in buffered solution. The log *D*_7.4_ obtained for oxaliplatin (−1.30) is in good agreement with the log *P*-value reported in literature (−1.39).^44^ The data for the 1-MDT-bearing prodrugs reveal that the free carboxylic acid (log *D*_7.4_: **SucEs/IdoCa** at −2.02; **SucCa/IdoCa** at −1.49) results in distinctly higher hydrophilicity compared to the two analogues with a free amino group (log *D*_7.4_: **SucEs/IdoEs** at −0.30; **SucCa/IdoEs** at −0.16). However, as the cellular uptake of the four 1-MDT-bearing complexes is quite similar (Figure 4), lipophilicity as an important parameter for the drug uptake can be widely excluded.

**Table 2:** log *D*_7.4_-Values for Selected Complexes

oxaliplatin	**OAc/OAc**	**SucEs/IdoCa**	**SucCa/IdoCa**	**SucEs/IdoEs**	**SucCa/IdoEs**
–1.30	–1.86	–2.02	–1.49	–0.30	–0.16

In general, the mode of uptake of the diverse platinum drugs is heavily discussed in the literature. In case of oxaliplatin, it is currently assumed that the drug enters the cancer cells mainly *via* active organic cation/carnitine transporter OCT1 and OCT2.^45^ Therefore, one explanation for the differences in the uptake of oxaliplatin and the novel platinum(IV) complexes could be a different recognition of the drugs by these uptake mechanisms. However, as all 1-MDT-bearing compounds enter the cells at similar efficiency, this indicates, on the one hand, that the difference in cytotoxicity between the 1-MDT-bearing drugs is not based on different drug uptake mechanisms. On the other hand, as oxaliplatin is accumulating with much higher potency, this suggests that the reduction (and thus oxaliplatin release) is an intracellular process.

### Selection of an IDO-Expressing Cancer Cell Model for IDO Inhibition Studies

**Figure 5:**
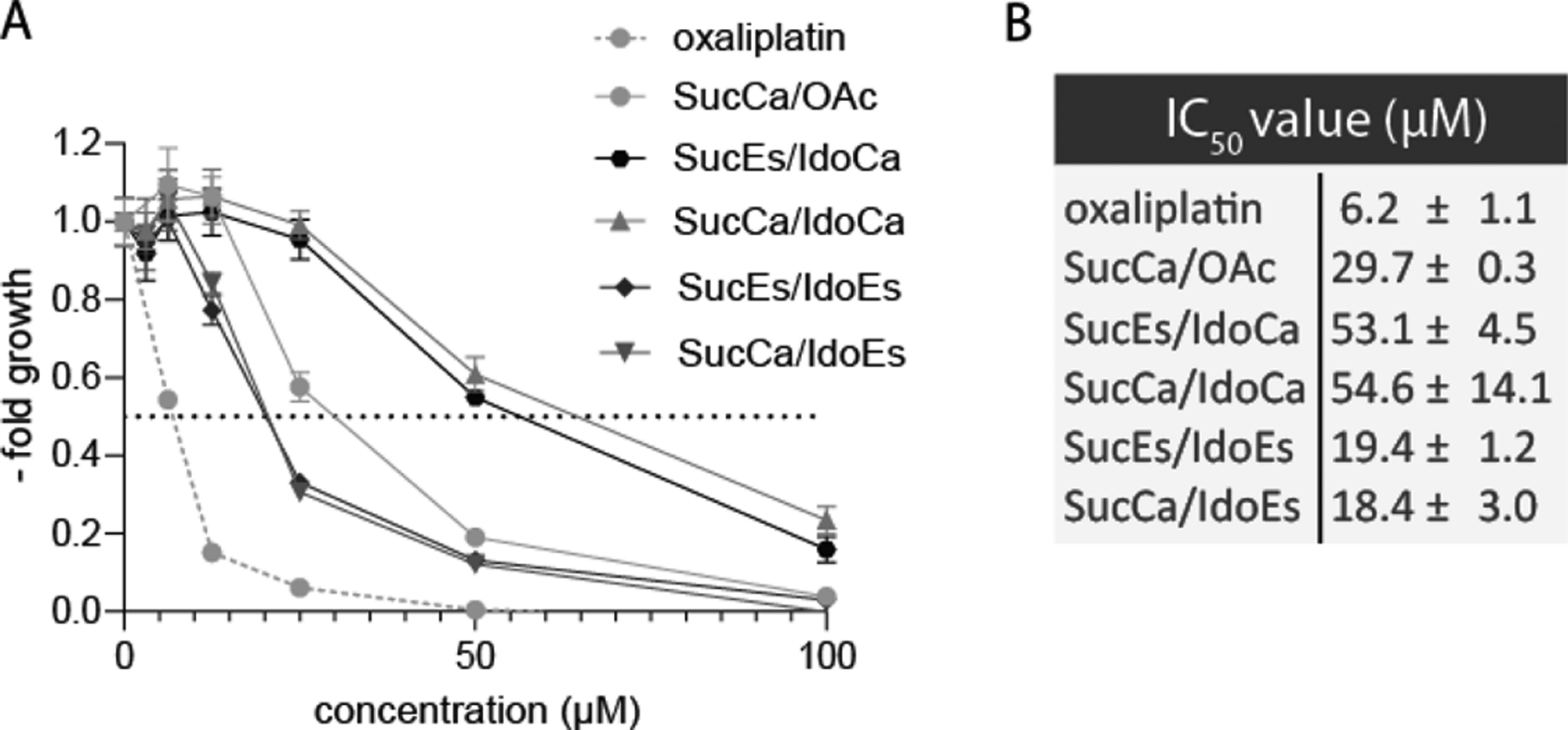
Long-term cytotoxicity assay in SKOV3 cancer cells. (A) Cells were treated with increasing concentrations of the indicated drugs for 168 h, fixed using ice-cold methanol, stained with crystal violet, and fluorescence intensity was measured. Fluorescence intensity was blotted as a full dose–response curve to calculate IC_50_ values. (B) Mean IC_50_ values given as mean ± SD.

In order to select an appropriate cell model for the analysis of the IDO-inhibitory potency of our new platinum(IV) prodrugs, as a first step we tested a panel of 13 cell lines from human and murine origin for their IDO messenger RNA (mRNA) levels by the real-time polymerase chain reaction (Figure S8). In agreement with the literature, SKOV3 ovarian carcinoma cells possessed very high IDO expression.^16,46^ Also, one patient-derived melanoma model VM7, which was established at our institute,^47^ was moderately positive for IDO expression. Unfortunately, of the tested murine colon cancer models, only CT-26 displayed some IDO mRNA levels, while all others were negative. Subsequently, we tested SKOV3 and VM7 for their sensitivity to our compound panel. Noteworthy, both cell models are characterized by a very low proliferation doubling time (Figure S9). Consequently, it is not surprising that both cell models were rather unresponsive to platinum treatment after 72 h (Table S3) as such anticancer drugs generally require a longer incubation time to show effects in slowly growing cancer cells.^48^ Thus, we extended the incubation time of our experiments with SKOV3 cells to 168 h and assessed the cell number using crystal violet stain. As expected, this effectively increased anticancer activity, especially for the platinum(IV) prodrugs (Figure 5). In line with the results in the colon cancer cell models, the less stable **SucCa/IdoEs** and **SucEs/IdoEs** derivatives showed higher (and more rapidly emerging) anticancer activity, resulting in lower IC_50_ values. All subsequent studies on the IDO inhibition of the new compounds were performed in SKOV3 cells.

### Kinetics of 1-MDT Release in SKOV3 Lysates

**Figure 6:**
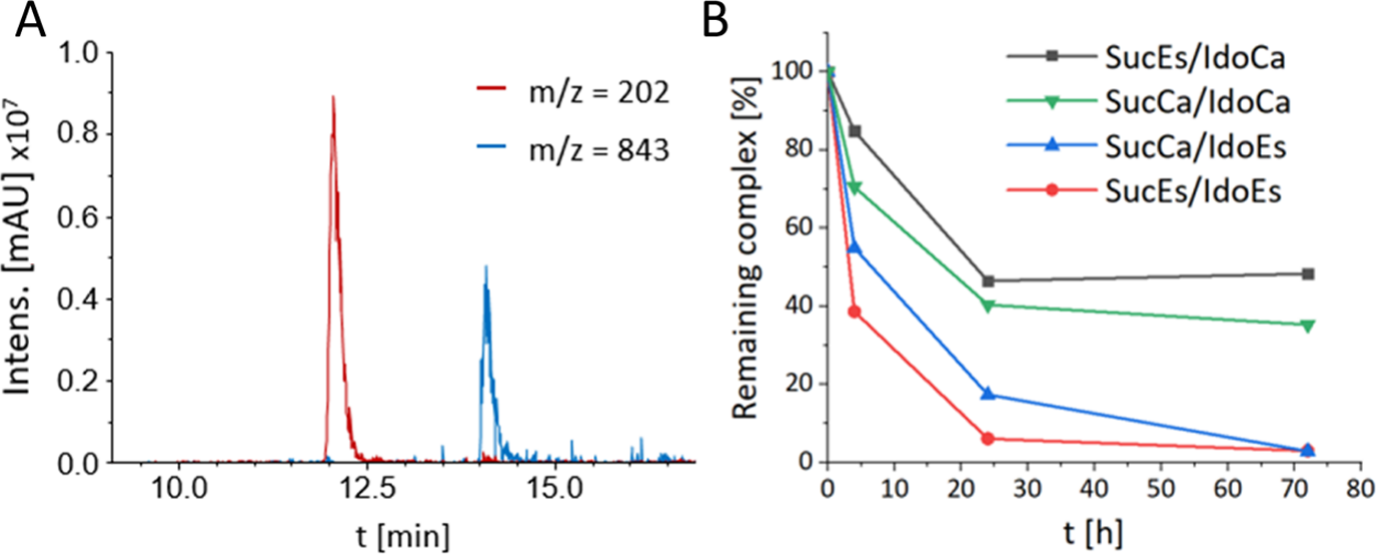
Reduction kinetics and release of 1-MDT in SKOV3 cell lysates. (A) Exemplary extracted ion chromatogram of 1-MDT release (*m*/*z* = 202) from **SucCa/IdoEs** (*m*/*z* = 843) after 24 h incubation. (B) Reduction kinetics of all succinimide complexes in SKOV3 cell lysates at 37 °C. At 0, 4, 24, and 72 h, methanol extracts were measured by HPLC with UV detection at 230 nm, as described in the experimental section. Data were normalized to the area under curve of the respective platinum(IV) complex at *t* = 0.

In order to determine whether our compounds are efficiently reduced and thereby release 1-MDT inside cancer cells, the succinimide prodrugs were incubated in SKOV3 lysates at 37 °C. Subsequently, at various time points up to 72 h, proteins were precipitated by addition of cold methanol (MeOH), and the supernatant was analyzed by liquid chromatography–mass spectrometry (LC–MS). We could nicely observe the released 1-MDT ligand (main fragment *m*/*z* = 202; [M – NH_2_]^+^; Figure 6A). However, we plotted the reduction of the peak intensities of the intact platinum(IV) complexes over time because of the limited solubility of 1-MDT in MeOH, leading to quantification problems. In line with the other results, the data revealed that the 1-MDT ester-linked complexes were reduced to a much higher extent than their 1-MDT-carbamato-counterparts. After 72 h, nearly 100% 1-MDT release was observed for **SucEs/IdoEs** and **SucCa/IdoEs**, whereas both, **SucEs/IdoCa** and **SucCa/IdoCa**, were still present as platinum(IV) species to ∼50% (Figure 6B). These results not only support the hypothesis that the reduction-mediated 1-MDT release is an intracellular event but also indicate that it is not dependent on intact cells.

### IDO Inhibition in Cell Culture

**Figure 7:**
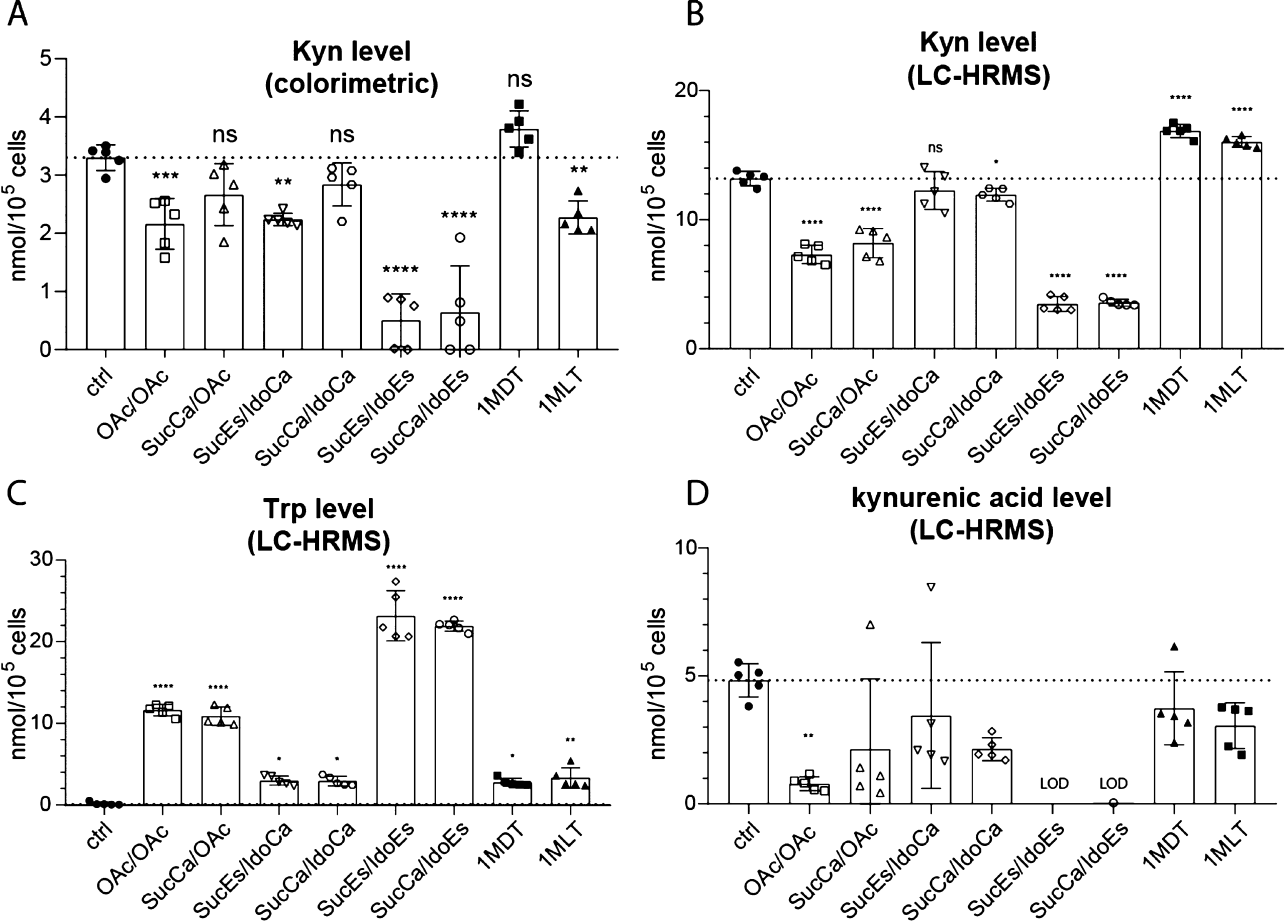
Inhibition of the enzymatic IDO activity of SKOV3 cells *in vitro*. (A) Colorimetric Kyn detection assay in serum-free supernatant of SKOV3 cells and LC–HRMS measurements of (B) Kyn, (C) Trp, and (D) kynurenic acid level normalized to cell number. Bars depict mean ± SD of five replicates normalized to cell number. LOD = limit of detection. Significance was calculated in comparison to control conditions by multiple comparison analysis [one-way analysis of variance (ANOVA)] and Dunnett post-hoc-test (*p < 0.05, **p < 0.01, ***p < 0.001, and ****p < 0.0001).

As already mentioned in the introduction section, the enzymatic activity of IDO leads to the catabolism of Trp to Kyn.^7^ To evaluate the activity of IDO after treatment with our platinum(IV) complexes, Kyn levels were analyzed in the supernatants of SKOV3 cells using a colorimetric method and liquid chromatography–high resolution mass spectrometry (LC–HRMS).^49^ As a first step, the assays were performed with the supernatants collected after 72 h treatment with both 1-MT enantiomers (1-MDT and 1-MLT). Noteworthy, due to their very poor cell permeability, for both drugs, very high concentrations are usually applied (2 mM).^50,51^ Moreover, although 1-MDT has better immunomodulatory properties *in vivo*, 1-MLT is the stronger IDO inhibitor *in vitro*.^10^ In good agreement with the literature, strong reductions in Kyn levels were observed with 1-MLT, while 1-MDT had borderline activity (Figure S10). As the LC–HRMS data indicated that serum-containing medium has already rather high Trp levels *per se* (data not shown), we subsequently used a modified setting for the investigation of our platinum(IV) drugs. In more detail, the cells were incubated with the drugs for 72 h under normal cell culture conditions. Then, the medium containing the test drugs and 10% FCS was replaced by serum-free medium for additional 24 h, which was then used for the measurements. Drug treatment had no impact on cell viability at the used concentrations of 50 μM (data not shown). Corresponding to the accelerated 1-MDT release, both fast-reducing compounds (**SucEs/IdoEs** and **SucCa/IdoEs**) showed stronger inhibition of IDO and therefore lower Kyn levels than the platinum(IV) reference complexes or the slow 1-MDT-releasing derivatives (**SucEs/IdoCa** and **SucCa/IdoCa**) (Figure 7A). The results were confirmed by LC–HRMS measurements of Kyn, Trp, and kynurenic acid, a downstream metabolite of the IDO pathway (Figure 7B–D).^52^ In addition, these data nicely show the metabolic connection between Kyn, Trp, and kynurenic acid: the stronger the IDO inhibition, the lower the Kyn and kynurenic acid levels and the higher the accumulation of Trp.

Noteworthy, the IDO inhibition of the two fast-reducing compounds was superior even to 1-MLT, which is especially interesting considering that the free IDO inhibitors were applied at a dose of 2 mM, compared to the release of max. 50 μM inhibitor in case of the platinum(IV) prodrugs. Consequently, these experiments not only confirm the release of functional 1-MDT from our new multi-modal complexes but also show that coupling to a platinum(IV) center is a very efficient way to enhance the transport of 1-MDT into cancer cells, where it is released after intracellular reduction.

### Tissue and Organ Distribution of the Maleimide-Conjugated Drugs *in Vivo*

**Figure 8:**
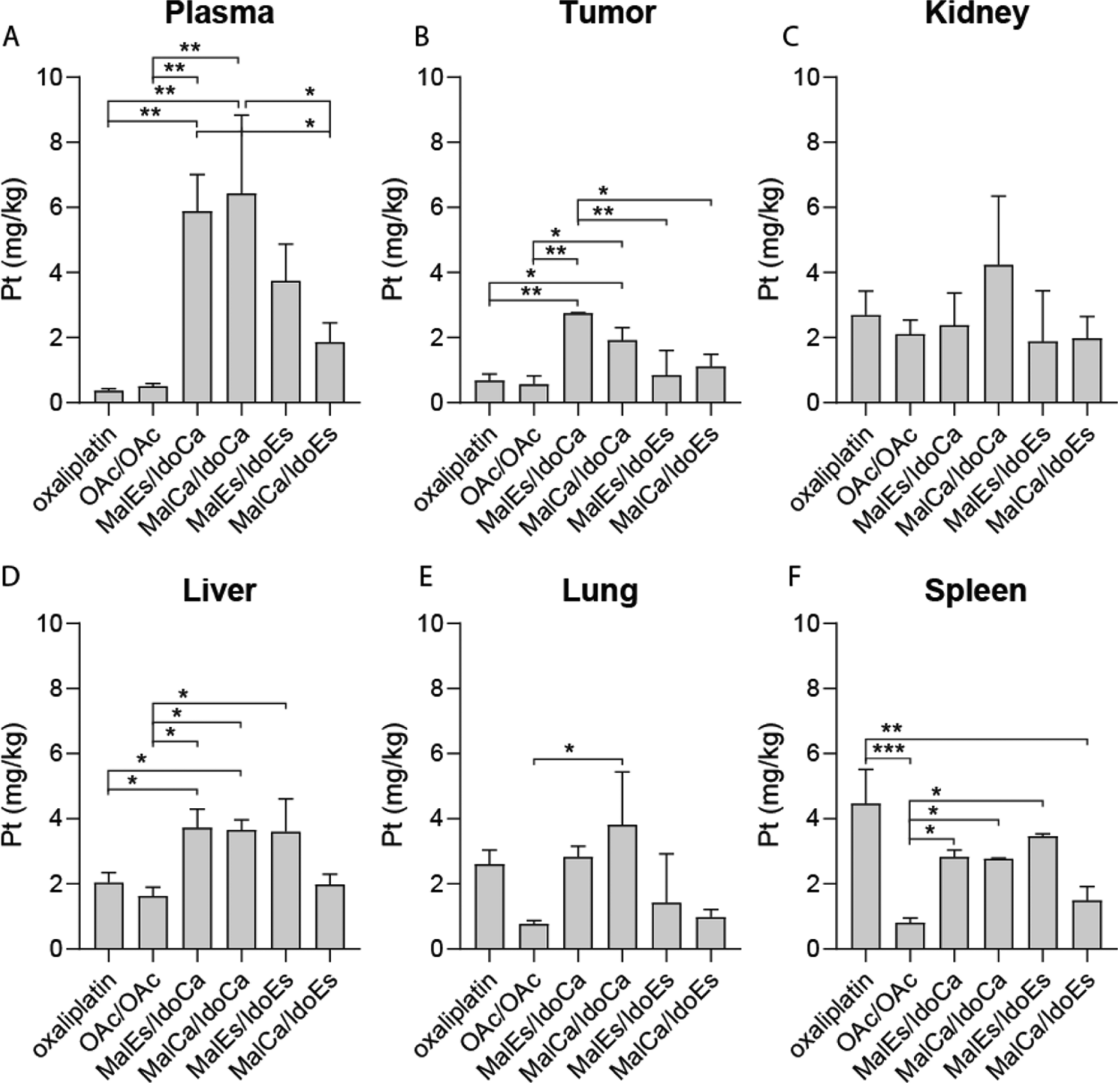
Plasma levels and drug distribution *in vivo*. SKOV3-bearing male SCID mice were treated once *via* the tail vein with the indicated drugs at doses equimolar to 9 mg/kg oxaliplatin. After 24 h, animals were sacrificed, and plasma (A), tumor tissue (B), and diverse organs (C–F) were collected. Platinum levels in isolated tissues were detected by ICP–MS and normalized to tissue weight. Significance was calculated by ordinary one-way ANOVA and Tukey’s multiple comparisons test (*p < 0.05, **p < 0.01, and ***p < 0.001).

In order to investigate the impact of the reduction kinetics on the pharmacological behavior of the drugs in the living organism, as a next step, SKOV3-bearing SCID mice were treated with our test compound panel at concentrations equimolar to 9 mg/kg oxaliplatin. After 24 h, the animals were sacrificed, and plasma, tumor, and organ samples were collected and snap-frozen in liquid nitrogen. Subsequently, platinum levels were analyzed by ICP–MS after microwave digestion. With regard to blood plasma, treatment with all maleimide derivatives led to distinctly higher platinum concentrations than oxaliplatin or **OAc/OAc** (∼12-fold in case of **MalEs/IdoCa** and **MalCa/IdoCa**). Noteworthy, there were also distinct differences between the fast- and the slow-reducing derivatives. Thus, the animals treated with the two fast-reducing derivatives had distinctly lower platinum levels (1.9 and 3.7 mg/kg for **MalCa/IdoEs** and **MalEs/IdoEs**, respectively) than the two slow-reducing drugs (5.9 and 6.4 mg/kg for **MalEs/IdoCa** and **MalCa/IdoCa**, respectively) (Figure 8A). Subsequently, especially the slow-reducing drugs showed pronounced tumor accumulation in comparison to both of the reference platinum complexes (Figure 8B–F). Noteworthy, as can be seen in the oxaliplatin- and **OAc/OAc**-treated animals, the malignant tissue is usually characterized by rather low drug levels resulting, for example, in tumor to organ ratios of ∼0.3 in case of the liver and kidney, respectively (Table S4). This ratio was distinctly improved by the albumin-targeted prodrug concept. Thus, especially in case of the slow-reducing agents, tumor to organ ratios of 0.7 and 1.2 for the liver and kidney, respectively, were observed. Overall, the impact of the albumin targeting on drug distribution is congruent with previous observations, as it results in a distinctly prolonged plasma half-life together with preferential uptake by the tumor tissue.^29,53^ However, the plasma levels detected for our new drugs were lower, when compared to data on oxaliplatin-releasing platinum(IV) maleimide derivatives without the 1-MDT ligand.^29^ This suggests that the additional 1-MDT moiety might lead to enhanced clearance of the albumin conjugate or faster reduction/oxaliplatin release. Whether this is specific to IDO-containing compounds or a general phenomenon for multi-modal maleimide derivatives needs to be addressed in subsequent studies.

### Anticancer and Immunomodulatory Activity against CT26 Colon Cancer Tumors *in Vivo*

As a first step, to gain more insights into the release of 1-MDT and subsequent IDO inhibition *in vivo*, the slow-reducing **MalEs/IdoCa** and the fast-reducing **MalCa/IdoEs** derivative were compared 24 h after treatment. To this end, the tumors were harvested and spiked with ^13^C-labeled metabolites. Tumor sample aliquots were 5-fold concentrated and the metabolites (Figure S11A) quantified by LC–HRMS. The relative quantification of 1-MDT in the SKOV3 tumor tissue revealed a significant 1-MDT release with both drugs. In good agreement with the ICP–MS data, the 1-MDT levels were ∼2-fold higher in the tumors of **MalEs/IdoCa**-treated animals than upon **MalCa/IdoEs** treatment (Figure S11B). Accordingly, the application of **MalEs/IdoCa** resulted in inhibition of IDO activity indicated by an improved Trp to Kyn ratio compared to solvent- or **MalCa/IdoEs**-treated animals (Figure S11C). Interestingly, when looking at the IDO downstream catabolite kynurenic acid (Figure S11D), enhanced signals were detected in the **MalCa/IdoEs**-treated tumors. This supports the hypothesis that slow-reducing maleimide derivatives are characterized by an enhanced plasma-half-life, which results in a prolonged IDO-inhibitory potential *in vivo*.

**Figure 9:**
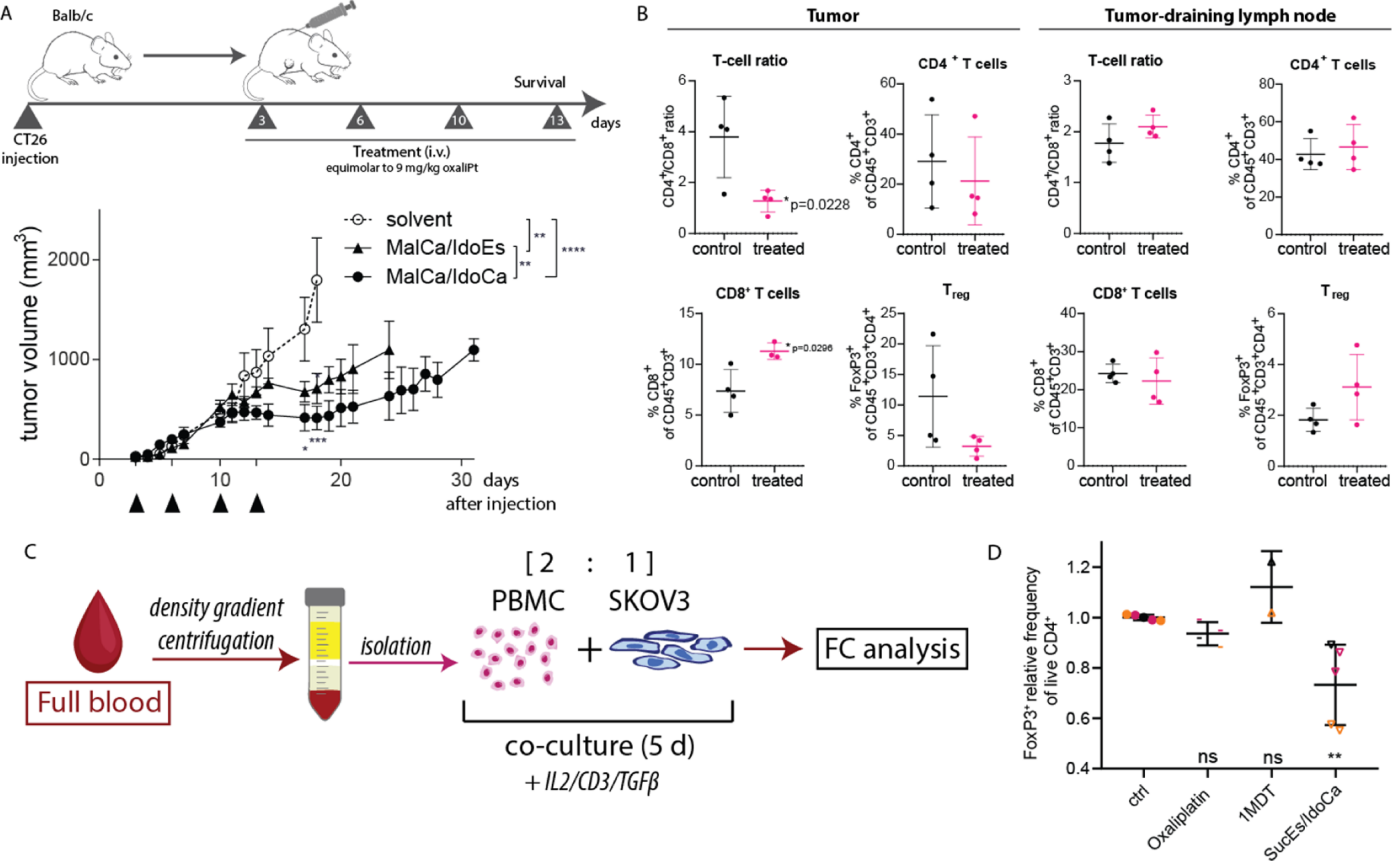
Anticancer activity and impact on tumor-infiltrating T-cell populations of our new 1-MDT-releasing drugs against CT26 allografts in immune-competent Balb/c mice. (A) Schematic timeline of the performed therapy experiment in CT26-bearing Balb/c mice. Tumor growth was measured daily by caliper after cell injection. Black arrows indicate treatments of solvent, **MalCa/IdoEs** or **MalCa/IdoCa** (iv) equimolar to 9 mg/kg oxaliplatin. Significance was calculated in comparison to the solvent group by two-way ANOVA, multiple comparison analysis, and Bonferroni post-hoc-test. (B) CT26-bearing Balb/c mice were treated once with drug doses of **MalEs/IdoCa** equimolar to 9 mg/kg oxaliplatin *via* the tail vein. After 24 h, animals were sacrificed, and tumors and tumor-draining lymph nodes were collected and analyzed for their T-cell populations by multicolor flow cytometry (FC). Significance was calculated in comparison to the solvent group and within each group by unpaired *t*-test (two-tailed) (*p < 0.05, **p < 0.01, ***p < 0.001, and ****p < 0.0001). (C) Schematic representation of the T_reg_ differentiation assay using a human PBMC/SKOV3 tumor cell co-culture model. (D) Relative frequency of T_reg_ in live CD4^+^ T cells (CD45^+^ CD3^+^ CD4^+^) from a co-culture with PBMC and SKOV3 tumor cells for oxaliplatin (10 μM), 1-MDT (50 μM), and **SucEs/IdoCa** (50 μM). Each value is normalized to control, and values from the same donor are presented in the same color (black, orange, or pink). Significance was calculated using one-way ANOVA with Dunnett’s multiple comparison tests.

Next, we were interested in the *in vivo* anticancer activity of our maleimide-containing compound panel. However, as both IDO inhibition and oxaliplatin need an active immune system for their activity against tumor cells, for these experiments, a switch to an immune-competent model was necessary. Therefore, we chose CT26 colon cancer allografts which expressed the “highest” IDO levels among the tested murine cell models (Figure S8B). The applied drug doses were equimolar to the maximal tolerated dose of oxaliplatin (9 mg/kg). In the first experiment, we compared the slow-reducing **MalCa/IdoCa** with the fast-reducing **MalCa/IdoEs** (Figure 9A). Both compounds had significant anticancer activity, which resulted in distinctly reduced tumor burden. However, **MalCa/IdoCa** was superior in its activity against the CT26 tumors compared to **MalCa/IdoEs** and led to a prolonged period of disease stabilization in all mice (tumor volumes of the individual animals and Kaplan–Meier curves for overall survival of the animals are shown in Figure S12). Subsequently, we also tested **MalEs/IdoEs** (Figure S13A), which was inactive, and **MalEs/IdoCa** (Figure S13B), which had activity comparable to **MalCa/IdoCa**. Finally, **MalCa/IdoCa** activity was directly compared to oxaliplatin (Figure S14). This experiment clearly indicates that **MalCa/IdoCa** was superior to oxaliplatin with respect to both, impact on tumor growth and overall survival. Taken together, these initial experiments indicate that slow platinum(IV) reduction kinetics (which in cell culture experiments results in low activity) are very beneficial in the *in vivo* setting, resulting in prolonged plasma half-life, enhanced tumor accumulation, and superior anticancer activity.

Finally, we were interested whether treatment with an 1-MDT-releasing platinum(IV) drug leads to a changed immune infiltration into CT26 tumors. To this end, flow cytometry of immune cells isolated from tumor tissue and from tumor-draining lymph nodes was performed 24 h after single treatment with **MalEs/IdoCa** (Figure 9B). Indeed, therapy led to a significant shift in the ratio of CD4^+^ (immunosuppressive) to CD8^+^ (immunostimulatory) T cells in the malignant tissue. This was based on a significant increase in the population of cytotoxic T cells, while there was a trend toward a reduced number of FoxP3^+^ (immunosuppressive) regulatory T cells (*T*_reg_). In contrast, drug treatment had no effect on the immune cell population in the respective tumor-draining lymph nodes. This indicates that the new drug indeed has the potential to reactivate the immune system inside the malignant tissue in a tumor-specific manner.

In order to investigate whether our prodrugs are also able to provoke a comparable immune response in human cells, we performed a series of co-culture experiments using isolated peripheral blood mononuclear cells (PBMCs) from three different healthy donors. T_reg_ differentiation and expansion were induced by co-culturing PBMCs and SKOV3 tumor cells for 5 days in the presence of T_reg_ differentiation medium [interleukin 2 (IL-2), CD3, and transforming growth factor β (TGFβ)] and nontoxic concentrations of oxaliplatin (10 μM), 1-MDT (50 μM), or **SucEs/IdoCa** (50 μM). Subsequently, the frequency of T_reg_ was assessed using multicolor flow cytometry (Figure 9C). Of note, in all three individuals, **SucEs/IdoCa** was superior to oxaliplatin or 1-MDT treatment and significantly reduced T_reg_ differentiation (Figure 9D). This suggests that through introduction of 1-MDT as a bioactive ligand, our prodrugs effectively reduced the amounts of tumor-suppressive T_reg_ also in a human co-culture setting.

## Conclusions

During the last few decades, there is increased understanding that the malignant tissue differs from healthy organs in multiple aspects.^54,55^ Consequently, it is not only of interest to develop new drugs with better tumor-targeting properties but also to consider the changed tumor microenvironment for tumor-specific therapy (*e.g.*, the tumor-promoting state of the immune cell population). This study reports on the first triple-action prodrugs of oxaliplatin, which is the current state of the art therapy against colon cancer.^2^ To this end, we used well-established platinum(IV) prodrugs (which themselves have often insufficient targeting properties due to premature activation in e.g. red blood cells) and improved their tumor delivery by attaching an albumin-targeting maleimide moiety as one axial ligand. Albumin is efficiently taken up und degraded by cancer cells due to their enhanced needs for nutrients.^25^ In the second axial position, the clinically investigated IDO inhibitor 1-MDT was attached.^8^ This approach results in the tumor-specific release of the immunomodulator together with oxaliplatin after activation by reduction inside the cancer cell.

In order to allow the selection of the best lead candidate for further (pre)clinical development, we prepared several derivatives, where 1-MDT has been attached to the molecules *via* different strategies. Noteworthy, these derivatives distinctly differed in their reduction behavior (fast reducing **SucEs/IdoEs** and **SucCa/IdoEs**; slow-reducing **SucEs/IdoCa** and **SucCa/IdoCa**). Overall, the low reductive stabilities observed for the **SucEs/IdoEs** and **SucCa/IdoEs** complexes were highly unexpected as similar oxaliplatin-based platinum(IV) complexes exhibit high reductive inertness.^29,40−42^ Subsequent analysis indeed confirmed that the complexes not only release (as expected) unmodified 1-MDT but also confirmed that this process has to be intracellular. This indicates that the coupling to a platinum(IV) center is also an efficient approach to deliver 1-MDT (which has rather low cell permeability) into cancer cells. Noteworthy, cell culture is rather limited when it comes to prediction of *in vivo* anticancer activity. This is especially true in case of maleimide-targeted drugs, as the moiety is not only prone to hydrolysis but also reacts with components of the cell culture medium (*e.g.* free cysteine). Moreover, cancer cells are usually “overfed” in the standard cell culture conditions, which can reduce their albumin uptake and catabolism. Consequently, *in vivo* experiments in tumor-bearing mice were performed to gain preliminary information on pharmacokinetic, immunomodulatory potential, and anticancer activity. In general, there are two basic screening approaches: on the one hand, human cancer cells can be tested in immune-deficient mice. On the other hand, immune-competent mice carrying murine allograft tumors can be used. For oxaliplatin prodrugs, immune-competent models are important, as it is well-known that the activity is dependent on the immune system.^3^ Consequently, anticancer activity and impact on tumor selectivity of the immune-inhibitory potential were tested in CT26 allografts. These investigations impressively confirm that there is no direct correlation between cell culture data and *in vivo* activity. The **IdoEs** complexes show higher activity in cell culture (due to faster reduction), however, lower activity in mice. In turn, the **IdoCa** compounds (with slower reduction) are widely inactive in common 48 h or 72 h viability assays but reveal higher tumor-inhibiting potential *in vivo*. The animal experiments also show that these complexes are able to inhibit IDO in the malignant tissue, which leads to tumor-specific changes in the T-cell population. However, our data also indicate that for the final selection of a lead candidate, further studies are required as our preliminary data reveal that the four 1-MDT-releasing derivatives differ in their pharmacological behavior (*e.g.*, plasma half-life). The exact reasons for these effects need to be further dissected and more in-depth studies are necessary to establish the best therapeutic scheme (*e.g.*, maximal tolerable dose and frequency of the treatment) for most favorable IDO inhibition.

In summary, in this study, we demonstrate that the design of albumin-targeted multi-modal prodrugs on platinum(IV) basis is a promising strategy to enhance the intracellular uptake of compounds with low cell permeability and additionally to improve their selective delivery into the malignant tissue. This should allow tumor-specific anticancer therapy supported by a favorable immune microenvironment.

## Experimental Section

### Synthesis

#### Materials and Methods

Potassium tetrachloridoplatinate was purchased from Johnson Matthey (Switzerland). Water for synthesis was taken from a reverse osmosis system and distilled twice before use. For HPLC measurements, Milli-Q water (18.2 MΩ·cm, Merck Milli-Q Advantage, Darmstadt, Germany) was used. Compound **5a**, other chemicals, and solvents were purchased from commercial suppliers (Sigma-Aldrich, Merck, Acros, Fluka, and Fisher Scientific). Oxaliplatin, complexes **7a**, **7b**, **OAc/OAc**, and 3-(1-methyl-1*H*-indol-3-yl)propanoic acid (**IPA**) were synthesized similar to methods described in the literature.^38,39,56,57^ Electrospray ionization (ESI) mass spectra were recorded on a Bruker amaZon SL ion trap mass spectrometer in the positive and/or negative mode by direct infusion at the Mass Spectrometry Centre of the University of Vienna. One- and two-dimensional ^1^H and ^13^C spectra were recorded on a Bruker AV Neo 500 or AV III 600 spectrometer at 298 K. For ^1^H and ^13^C NMR spectra, the solvent residual peak was taken as an internal reference. The ^1^H and ^13^C NMR spectra of the final compounds are depicted in Figures S17–S24. Purification by preparative reverse phase (RP) HPLC was performed on an Agilent 1200 series system using a Waters XBridge C18 column (19 × 250 mm). Elemental analysis measurements were carried out on a PerkinElmer 2400 CHN elemental analyzer at the Microanalytical Laboratory of the University of Vienna and are within ±0.4%, confirming >95% purity. Of course, the content of TFA and water can vary between different batches of the same compound. In addition, UHPLC chromatograms of the final compounds can be seen in Figures S25–S32. For NMR numbering of the final compounds, see Scheme S1.

#### General Procedure A for Isocyanate Formation from Carboxylic Acids

The carboxylic acid was dissolved in anhydrous toluene, and 1.2 equiv of TEA was added. DPPA (1 equiv) was slowly added, and the mixture was stirred under Ar at RT for 5.5 h. A saturated NaHCO_3_ solution was added to the mixture, the phases were separated, and the organic layer was washed with brine, dried over Na_2_SO_4_, filtered, and subsequently refluxed under Ar for 17 h. The solvents were removed *in vacuo* to obtain the crude isocyanate which was used without further purification.

##### *tert*-Butyl 1-Methyl-d-tryptophanate (**1**)

1-MDT (500 mg, 2.29 mmol) was suspended in *tert*-butyl acetate (10 mL) and cooled to 0 °C. Perchloric acid (60%) (0.38 mL, 3.43 mmol, 1.5 equiv) was added slowly, and the reaction mixture was stirred at RT for 19 h. 0.5 M HCl (4 × 30 mL) was added to the reaction mixture, and the phases were separated. The combined aqueous layers were basified to pH 9–10 by addition of 10% K_2_CO_3_ solution, extracted with DCM (4 × 50 mL), dried over MgSO_4_, and evaporated to dryness to obtain **1** (244 mg, 38%) as a colorless oil. ^1^H NMR (500 MHz, DMSO-*d*_6_): δ 7.53 (d, *J* = 7.9 Hz, 1H), 7.36 (d, *J* = 8.2 Hz, 1H), 7.15–7.07 (m, 2H), 7.02–6.98 (m, 1H), 3.72 (s, 3H), 3.47 (t, *J* = 6.4 Hz, 1H), 2.95 (dd, *J* = 14.2, 6.3 Hz, 1H), 2.85 (dd, *J* = 14.2, 6.7 Hz, 1H), 1.74 (br s, 2H), 1.29 (s, 9H). MS (*m*/*z*): calcd for C_16_H_23_N_2_O_2_ (M + H)^+^, 275.18; found, 275.15.

##### *tert*-Butyl *N*^α^-(1*H*-Imidazole-1-carbonyl)-1-methyl-d-tryptophanate (**2**)

1,1′-Carbonyldiimidazole (249 mg, 1.54 mmol, 2 equiv) was dissolved in DCM (10 mL), and DIPEA (0.34 mL, 1.92 mmol, 2.5 equiv) was added. **1** (211 mg, 0.77 mmol, 1 equiv) was slowly added to the solution and stirred at RT for 4 h. The reaction mixture was washed with half-saturated aqueous NH_4_Cl solution, and the organic phase was dried over MgSO_4_ and evaporated to dryness. The crude product was purified by flash column chromatography (40% hexane in EtOAc) to obtain title compound **2** (150 mg, 52%) as a white solid. ^1^H NMR (500 MHz, DMSO-*d*_6_): δ 8.86 (d, *J* = 7.6 Hz, 1H), 8.24 (m, 1H), 7.69 (t, *J* = 1.4 Hz, 1H), 7.58 (d, *J* = 7.9 Hz, 1H), 7.38 (d, *J* = 8.2 Hz, 1H), 7.19 (s, 1H), 7.15–7.11 (m, 1H), 7.05–7.00 (m, 2H), 4.47 (ddd, *J* = 9.5, 7.6, 5.6 Hz, 1H), 3.72 (s, 3H), 3.27 (dd, *J* = 14.6, 5.5 Hz, 1H), 3.17 (dd, *J* = 14.6, 9.4 Hz, 1H), 1.34 (s, 9H). MS (*m*/*z*): calcd for C_20_H_24_N_4_O_3_Na (M + Na)^+^, 391.17; found, 391.19.

##### *N*^α^-(*tert*-Butoxycarbonyl)-1-methyl-d-tryptophan (**4**)

1-MDT (500 mg, 2.29 mmol) was suspended in H_2_O/dioxane 1:1 (12 mL). NaOH (229 mg, 5.73 mmol, 2.5 equiv) was added. Di-*t*-butyl dicarbonate was dissolved in H_2_O/dioxane 1:2 (2 mL) and added to the aforementioned solution. The reaction mixture was stirred at RT for 4 h, poured into H_2_O (25 mL), adjusted to pH 2 with 1 M HCl, and extracted with EtOAc (3 × 25 mL). The organic layers were combined, washed with brine, dried over NaSO_4_, and evaporated to dryness to give **4** (524 mg, 71%) as a white powder. ^1^H NMR (500 MHz, CDCl_3_): δ 7.59 (d, *J* = 7.9 Hz, 1H), 7.29 (d, *J* = 8.2 Hz, 1H), 7.23 (t, *J* = 7.6 Hz, 1H), 7.11 (t, *J* = 7.5 Hz, 1H), 6.92 (s, 1H), 5.08–4.96 (m, 1H), 4.70–4.44 (m, 1H), 3.75 (s, 3H), 3.37–3.26 (m, 2H), 1.43 (s, 9H). MS (*m*/*z*): calcd for C_17_H_21_N_2_O_4_ (M – H)^−^, 317.15; found, 316.93.

##### 6-(2,5-Dioxopyrrolidine-1-yl)hexanoic Acid (**5b**)

Succinic anhydride (2.0 g, 20.0 mmol) was suspended in acetic acid (30 mL), and 6-aminohexanoic acid (2.62 g, 20.0 mmol, 1 equiv) was added. The reaction mixture was refluxed for 3 h, and the solvent was removed *in vacuo*. The crude product was purified by flash column chromatography (20% hexane in EtOAc) to obtain **5b** (2.46 g, 57%) as a white solid. ^1^H NMR (500 MHz, CDCl_3_): δ 3.54–3.47 (m, 2H), 2.70 (s, 4H), 2.35 (t, *J* = 7.4 Hz, 2H), 1.70–1.63 (m, 2H), 1.63–1.55 (m, 2H), 1.40–1.31 (m, 2H). MS (*m*/*z*): calcd for C_10_H_15_NO_4_Na (M + Na)^+^, 236.09; found, 236.07.

##### 1-(5-Isocyanatopentyl)-1*H*-pyrrole-2,5-dione (**6a**)

Compound **6a** was synthesized according to general procedure A using 6-maleimidohexanoic acid (1.0 g, 4.73 mmol), toluene (25 mL), TEA (0.79 mL, 5.68 mmol), and DPPA (1.07 mL, 4.97 mmol). Yield: 1.10 g of crude colorless oil. ^1^H NMR (500 MHz, CDCl_3_): δ 6.69 (s, 2H), 3.53 (t, *J* = 7.2 Hz, 2H), 3.30 (t, *J* = 6.6 Hz, 2H), 1.67–1.59 (m, 4H), 1.42–1.33 (m, 2H).

##### 1-(5-Isocyanatopentyl)pyrrolidine-2,5-dione (**6b**)

Compound **6a** was synthesized according to general procedure A using **5b** (1.0 g, 4.69 mmol), anh. toluene (40 mL), TEA (0.78 mL, 5.63 mmol, 1.2 equiv), and DPPA (1.01 mL, 4.69 mmol, 1 equiv). Yield: 0.92 g of crude colorless oil. ^1^H NMR (500 MHz, DMSO-*d*_6_): δ 3.36–3.31 (m, 4H), 2.61 (s, 4H), 1.58–1.51 (m, 2H), 1.51–1.43 (m, 2H), 1.30–1.22 (m, 2H).

##### (*OC*-6-34)-[(1*R*,2*R*)-1,2-Cyclohexanediamino][5-(2,5-dioxo-2,5-dihydro-1*H*-pyrrol-1-yl)pentylcarbamato]hydroxidooxalatoplatinum(IV) (**8a**)

**7** (555 mg, 1.29 mmol) was suspended in anh. DMSO (50 mL). A solution of **6a** (268 mg, 1.29 mmol, 1 equiv) in anh. DMSO (1 mL) was added to the mixture over a period of 16 h with the help of a syringe pump. The reaction mixture was stirred further at RT for 3 h, and the solvent was removed *in vacuo* at 50 °C. MeOH was added, and the crude product precipitated by addition of methyl *tert*-butyl ether. The resulting white powder (680 mg) was used without further purification. For characterization purpose, a small fraction was purified *via* preparative RP-HPLC [16% acetonitrile (MeCN) (+0.1% HCOOH) in H_2_O (+0.1% HCOOH); isocratic]. ^1^H NMR (500 MHz, DMSO-*d*_6_): δ 10.00–9.57 (m, 1H), 8.28 (br s, 1H), 7.64 (br s, 1H), 7.07 (br s, 1H), 7.00 (s, 2H), 6.41–6.01 (m, 1H), 2.94–2.73 (m, 2H), 2.59–2.51 (m, 2H), 2.26 (br s, 1H), 2.13–2.00 (m, 2H), 1.55–1.22 (m, 8H), 1.20–1.03 (m, 4H). MS (*m*/*z*): calcd for C_18_H_28_N_4_O_9_NaPt (M + Na)^+^, 662.14; found, 662.04.

##### (*OC*-6-34)-[(1*R*,2*R*)-1,2-Cyclohexanediamino][5-(2,5-dioxo-2,5-dihydro-1*H*-pyrrolidin-1-yl)pentylcarbamato]hydroxidooxalatoplatinum(IV) (**8b**)

**7** (200 mg, 0.46 mmol) was suspended in anh. DMSO (2.5 mL). A solution of **6b** (97 mg, 0.46 mmol, 1 equiv) in anh. DMSO (1 mL) was added to the mixture over a period of 17 h with the help of a syringe pump. The reaction mixture was stirred further at RT for 3 h, the solvent was removed *in vacuo* at 50 °C, and the crude product was purified by preparative RP-HPLC [12% MeCN (+0.1% TFA) in H_2_O (+0.1% TFA); isocratic] to obtain title compound **8b** (117 mg, 36%) as a white powder. ^1^H NMR (500 MHz, DMSO-*d*_6_): δ 10.04–9.40 (m, 1H), 8.80–8.11 (m, 1H), 7.89–7.54 (m, 1H), 7.17–6.93 (m, 1H), 6.47–5.90 (m, 1H), 3.31 (t, *J* = 7.1 Hz, 2H), 2.92–2.74 (m, 2H), 2.61 (s, 4H), 2.57–2.52 (m, 2H), 2.17–1.99 (m, 2H), 1.56–1.23 (m, 8H), 1.21–1.06 (m, 4H). MS (*m*/*z*): calcd C_18_H_30_N_4_O_9_NaPt (M + Na)^+^, 664.16; found, 664.15.

##### (*OC*-6-34)-[(*R*)-(1-(*tert*-Butoxy)-3-(1-methyl-1*H*-indol-3-yl)-1-oxopropan-2-yl)carbamato][(1*R*,2*R*)-1,2-cyclohexanediamino]hydroxidooxalatoplatinum(IV) (**9**)

In a dry flask, **2** (140 mg, 0.38 mmol) was dissolved in anh. DMSO (1.5 mL) and stirred under Ar at 85 °C for 5 h in order to obtain isocyanate **3**. The reaction mixture was cooled to RT and added to a suspension of **7** (164 mg, 0.38 mmol, 1 equiv) in anh. DMSO (1.5 mL) over a period of 17 h with the help of a syringe pump. The solvent was removed *in vacuo* at 50 °C, and the crude product was purified by preparative RP-HPLC [38% MeCN (+0.1% HCOOH) in H_2_O (+0.1% HCOOH); isocratic] to obtain **9** (70 mg, 25%) as a yellow solid. ^1^H NMR (500 MHz, DMSO-*d*_6_): δ 9.79–9.40 (m, 1H), 8.21 (br s, 1H), 7.66 (br s, 1H), 7.48 (d, *J* = 7.9 Hz, 1H), 7.37 (d, *J* = 8.2 Hz, 1H), 7.16–6.98 (m, 4H), 6.36–6.01 (m, 1H), 4.28–4.04 (m, 1H), 3.73 (s, 3H), 2.99 (d, *J* = 6.8 Hz, 2H), 2.55–2.52 (m, 2H), 2.36 (s, 1H), 2.13–1.99 (m, 2H), 1.54–1.39 (m, 3H), 1.32–1.19 (m, 10H), 1.17–0.99 (m, 2H). MS (*m*/*z*): calcd C_25_H_36_N_4_O_9_NaPt (M + Na)^+^, 754.20; found, 754.21.

#### General Procedure B for *t*-Butyl- and Boc-Deprotection

The protected complex was dissolved in DCM (∼40 mM), and 10% TFA (v/v) was added. The reaction mixture was stirred at RT for 1 h, the solvents were evaporated, and the crude product was purified by preparative RP-HPLC.

##### (*OC*-6-34)-[(*R*)-2-Ammonio-3-(1-methyl-1*H*-indol-3-yl)propanoato][(1*R*,2*R*)-1,2-cyclohexanediamino][5-(2,5-dioxo-2,5-dihydro-1*H*-pyrrol-1-yl)pentylcarbamato]oxalatoplatinum(IV) Trifluoroacetate (**MalCa/IdoEs**)

**4** (169 mg, 0.53 mmol) was dissolved in DMF (6 mL), and TEA (96 μL, 0.69 mmol, 1.3 equiv) and TBTU (188 mg, 0.58 mmol, 1.1 equiv) were added. After 10 min at RT, crude **8a** (340 mg) was added and stirred further at RT for 17 h. The solvent was evaporated under reduced pressure, and the crude product was purified by preparative RP-HPLC [42% MeCN (+0.1% HCOOH) in H_2_O (+0.1% HCOOH); isocratic] to obtain ***Boc*-MalCa/IdoEs** (52 mg, 8% over two steps) as a yellow solid. Deprotection was performed according to general procedure B starting from ***Boc*-MalCa/IdoEs** (51 mg, 0.05 mmol). Preparative RP-HPLC conditions: 28% MeCN (+0.1% TFA) in H_2_O (+0.1% TFA); isocratic. Yield: 32 mg (58%) of yellow solid. ^1^H NMR (600 MHz, DMSO-*d*_6_; for NMR numbering, see Scheme S1): δ 9.93–9.43 (m, 1H, DACH-1), 8.83–8.59 (m, 1H, DACH-1), 8.13–8.05 (br s, 1H, DACH-1), 8.05–7.96 (m, 3H, IDO-14), 7.64–7.54 (m, 2H, 1× DACH-1, IDO-5), 7.43 (d, *J* = 8.3 Hz, 1H, IDO-8), 7.21–7.15 (m, 1H, IDO-7), 7.13 (s, 1H, IDO-2), 7.07 (t, *J* = 7.4 Hz, 1H, IDO-6), 7.00 (s, 2H, MAL-3), 6.87 (t, *J* = 5.5 Hz, 1H, MAL-9), 4.16–4.08 (m, 1H, IDO-12), 3.75 (s, 3H, IDO-10), 3.35–3.28 (m, 3H, MAL-4, 1× IDO-11), 3.03 (dd, *J* = 15.2, 8.2 Hz, 1H, IDO-11), 2.89 (qd, *J* = 13.3, 6.4 Hz, 2H, MAL-8), 2.68–2.58 (m, 2H, DACH-2), 2.18 (d, *J* = 11.2 Hz, 1H, DACH-3), 2.09 (d, *J* = 9.7 Hz, 1H, DACH-3), 1.53 (d, *J* = 11.6 Hz, 2H, 2× DACH-4), 1.50–1.39 (m, 4H, MAL-5, 2× DACH-3), 1.36 (dt, *J* = 14.5, 7.1 Hz, 2H, MAL-7), 1.22–1.00 (m, 4H, 2× DACH-4, MAL-6). ^13^C NMR (151 MHz, DMSO-*d*_6_): δ 174.1 (IDO-13), 171.1 (2× MAL-2), 164.2 (MAL-10), 163.7, 163.7 (2× oxalate), 136.8 (IDO-9), 134.5 (2× MAL-3), 129.0 (IDO-2), 127.2 (IDO-4), 121.4 (IDO-7), 118.7 (IDO-6), 118.4 (IDO-5), 109.9 (IDO-8), 106.7 (IDO-3), 61.5, 60.3 (2× DACH-2), 53.1 (IDO-12), 40.7 (MAL-8), 37.0 (MAL-4), 32.4 (IDO-10), 31.00, 30.8 (2× DACH-3), 29.0 (MAL-7), 27.7 (MAL-5), 26.8 (IDO-11), 23.6, 23.6 (2× DACH-4), 23.4 (MAL-6). MS (*m*/*z*): calcd C_30_H_40_N_6_O_10_NaPt (M + Na)^+^, 840.25; found, 840.24. EA calcd C_30_H_40_N_6_O_10_Pt·1.5TFA: C, 39.21; H, 4.14; N, 8.31. Found: C, 39.28; H, 4.01; N, 8.50.

##### (*OC*-6-24)-[(*R*)-(1-Carboxy-2-(1-methyl-1*H*-indol-3-yl)ethyl)carbamato][(1*R*,2*R*)-1,2-cyclohexanediamino][5-(2,5-dioxo-2,5-dihydro-1*H*-pyrrol-1-yl)pentylcarbamato]oxalatoplatinum(IV) (**MalCa/IdoCa**)

In a dry flask, **9** (35 mg, 0.05 mmol) was dissolved in anh. DMF (1 mL), and **6a** (60 mg, 0.29 mmol, 6 equiv) was added. The reaction mixture was stirred under Ar at RT for 18 h. The solvent was evaporated *in vacuo*, and the crude product was purified by preparative RP-HPLC [51% MeCN (+0.1% HCOOH) in H_2_O (+0.1% HCOOH); isocratic] to obtain ***t*-butyl MalCa/IdoCa** (33 mg, 73%) as a yellow solid. Deprotection was performed according to general procedure B starting from ***t*-butyl MalCa/IdoCa** (35 mg, 0.04 mmol). Preparative RP-HPLC conditions: 30% MeCN (+0.1% TFA) in H_2_O (+0.1% TFA); isocratic. Yield: 19 mg (56%) of yellow solid. ^1^H NMR (600 MHz, DMSO-*d*_6_; for NMR numbering, see Scheme S1): δ 9.80–9.44 (m, 1H, DACH-1), 9.25 (br s, 1H, DACH-1), 8.55 (br s, 1H, DACH-1), 8.31 (s, 1H, DACH-1), 7.48 (d, *J* = 7.9 Hz, 1H, IDO-5), 7.36 (d, *J* = 8.2 Hz, 1H, IDO-8), 7.12 (t, *J* = 7.6 Hz, 1H, IDO-7), 7.06 (s, 1H, IDO-2), 7.00 (t, *J* = 7.4 Hz, 1H, IDO-6), 6.97 (s, 2H, 2× MAL-3), 6.80–6.59 (m, 1H, MAL-9), 6.58–6.05 (m, 1H, IDO-14), 4.31–4.12 (m, 1H, IDO-12), 3.73 (s, 3H, IDO-10), 3.36–3.34 (m, 2H, MAL-4), 3.08 (dd, *J* = 14.8, 4.9 Hz, 1H, IDO-11), 3.02 (dd, *J* = 14.6, 7.8 Hz, 1H, IDO-11), 2.93–2.81 (m, 2H, MAL-8), 2.59–2.52 (m, 2H, 2× DACH-2), 2.18–2.09 (m, 2H, 2× DACH-3), 1.54–1.47 (m, 2H, 2× DACH-4), 1.47–1.41 (m, 2H, MAL-N-5), 1.41–1.26 (m, 4H, 2× DACH-3, MAL-7), 1.20–1.03 (m, 4H, 2× DACH-4, MAL-6). ^13^C NMR (151 MHz, DMSO-*d*_6_): δ 174.0 (IDO-13), 171.2 (MAL-2), 164.5 (MAL-10), 163.9 (IDO-15), 163.4 (oxalate), 136.6 (IDO-9), 134.5 (2× MAL-3), 128.1 (IDO-2), 127.6 (IDO-4), 121.2 (IDO-7), 118.7 (IDO-6), 118.5 (IDO-5), 109.7 (IDO-8), 109.2 (IDO-3), 61.1, 61.0 (2× DACH-2), 55.3 (IDO-12), 40.7 (MAL-8), 37.1 (MAL-4), 32.4 (IDO-10), 31.1 (DACH-3), 29.1 (MAL-7), 27.8 (MAL-5), 26.7 (IDO-11), 23.7 (MAL-6), 23.5, 23.5 (2× DACH-4). MS (*m*/*z*): calcd C_31_H_40_N_6_O_12_NaPt (M + Na)^+^, 906.22; found, 906.22. EA calcd C_31_H_40_N_6_O_12_Pt·1.5H_2_O: C, 40.88; H, 4.76; N, 9.23. Found: C, 40.65; H, 4.54; N, 9.17.

##### (*OC*-6-34)-[(*R*)-(1-Carboxy-2-(1-methyl-1*H*-indol-3-yl)ethyl)carbamato][(1*R*,2*R*)-1,2-cyclohexanediamino][6-(2,5-dioxo-2,5-dihydro-1*H*-pyrrol-1-yl)hexanoato]oxalatoplatinum(IV) (**MalEs/IdoCa**)

**5a** (11 mg, 0.05 mmol, 1.2 equiv) was dissolved in DMF (1 mL). Subsequently, *N*-ethylmaleimide (28 mg, 0.23 mmol, 5 equiv), TEA (12.5 μL, 0.09 mmol, 2 equiv), and TBTU (22 mg, 0.07 mmol, 1.5 equiv) were added. After 15 min at RT, **9** (33 mg, 0.05 mmol, 1 equiv) was added and stirred further at RT for 17 h. The solvent was evaporated under reduced pressure, and the crude product was purified by preparative RP-HPLC [50% MeCN (+0.1% HCOOH) in H_2_O (+0.1% HCOOH); isocratic] to yield ***t*-butyl MalEs/IdoCa** (25 mg, 60%) as a yellow solid. Deprotection was performed according to general procedure B starting from ***t*-butyl MalEs/IdoCa** (25 mg, 0.03 mmol). Preparative RP-HPLC conditions: 35% MeCN (+0.1% TFA) in H_2_O (+0.1% TFA); isocratic. Yield: 19 mg (74%) of yellow solid. ^1^H NMR (600 MHz, DMSO-*d*_6_; for NMR numbering, see Scheme S1): δ 12.57 (br s, 1H, IDO–COOH), 9.27 (t, *J* = 9.4 Hz, 1H, DACH-1), 8.48 (br s, 1H, DACH-1), 8.33 (br s, 1H, DACH-1), 8.25 (t, *J* = 9.7 Hz, 1H, DACH-1), 7.49 (d, *J* = 7.9 Hz, 1H, IDO-5), 7.37 (d, *J* = 8.2 Hz, 1H, IDO-8), 7.15–7.10 (m, 1H, IDO-7), 7.07 (s, 1H, IDO-2), 7.04–6.99 (m, 1H, IDO-6), 6.99 (s, 2H, MAL-3), 6.63–6.09 (m, 1H, IDO-14), 4.31–4.14 (m, 1H, IDO-12), 3.74 (s, 3H, IDO-10), 3.39–3.35 (m, 2H, MAL-4), 3.08 (dd, *J* = 14.6, 5.0 Hz, 1H, IDO-11), 3.03 (dd, *J* = 14.8, 7.8 Hz, 1H, IDO-11), 2.58–2.51 (m, 2H, 2× DACH-2), 2.29–2.18 (m, 2H, MAL-8), 2.15–2.06 (m, 2H, 2× DACH-3), 1.53–1.46 (m, 2H, 2× DACH-4), 1.46–1.36 (m, 5H, MAL-5, MAL-7, 1× DACH-3), 1.36–1.27 (m, 1H, DACH-3), 1.20–1.07 (m, 4H, 2× DACH-4, MAL-6). ^13^C NMR (151 MHz, DMSO-*d*_6_): δ 180.8 (MAL-10), 173.8 (IDO-13), 171.1 (2× MAL-2), 163.8 (IDO-15), 163.4, 163.3 (2× oxalate), 136.4 (IDO-9), 134.5 (2× MAL-3), 128.0 (IDO-2), 127.5 (IDO-4), 121.0 (IDO-7), 118.5 (IDO-6), 118.4 (IDO-5), 109.6 (IDO-8), 109.1 (IDO-3), 61.1, 60.9 (2× DACH-2), 55.2 (IDO-12), 36.9 (MAL-4), 35.5 (MAL-8), 32.3 (IDO-10), 30.9, 30.9 (2× DACH-3), 27.7 (MAL-5), 26.6 (IDO-11), 25.6 (MAL-6), 24.8 (MAL-7), 23.5, 23.5 (2× DACH-4). MS (*m*/*z*): calcd C_31_H_39_N_5_O_12_NaPt (M + Na)^+^, 891.21; found, 891.20. EA calcd C_31_H_39_N_5_O_12_Pt·0.5TFA·1H_2_O: C, 40.72; H, 4.43; N, 7.42. Found: C, 40.82; H, 4.34; N, 7.43.

##### (*OC*-6-44)-[(*R*)-2-Ammonio-3-(1-methyl-1*H*-indol-3-yl)propanoato][(1*R*,2*R*)-1,2-yclohexanediamino][6-(2,5-dioxo-2,5-dihydro-1*H*-pyrrol-1-yl)hexanoato]oxalatoplatinum(IV) Trifluoroacetate (**MalEs/IdoEs**)

**4** (203 mg, 0.64 mmol, 1.1 equiv) and **5a** (135 mg, 0.64 mmol, 1.1 equiv) were dissolved in DMF (6 mL), and TEA (242 μL, 1.73 mmol, 3 equiv) and TBTU (465 mg, 1.45 mmol, 2.5 equiv) were added. After 10 min at RT, **9** (250 mg, 0.58 mmol, 1 equiv) was added and stirred further at RT for 16 h. The solvent was evaporated under reduced pressure, and the crude product was purified by preparative RP-HPLC [gradient: 35–50% MeCN (+0.1% HCOOH) in H_2_O (+0.1% HCOOH) over 20 min] to obtain ***Boc*-MalEs/IdoEs** (140 mg, 52%) as a yellow solid. Deprotection was performed according to general procedure B starting from ***Boc*-MalEs/IdoEs** (140 mg, 0.15 mmol). Preparative RP-HPLC conditions: 30% MeCN (+0.1% TFA) in H_2_O (+0.1% TFA); isocratic. Yield: 104 mg (69%) of yellow solid. ^1^H NMR (600 MHz, DMSO-*d*_6_; for NMR numbering, see Scheme S1): δ 8.57–8.40 (m, 1H, DACH-1), 8.36–8.24 (m, 1H, DACH-1), 8.24–8.13 (m, 1H, DACH-1), 8.02 (br s, 3H, IDO-14), 7.68–7.51 (m, 2H, 1× DACH-1, IDO-5), 7.43 (d, *J* = 8.3 Hz, 1H, IDO-8), 7.21–7.15 (m, 1H, IDO-7), 7.12 (s, 1H, IDO-2), 7.09–7.05 (m, 1H, IDO-6), 7.00 (s, 2H, MAL-3), 4.14–4.04 (m, 1H, IDO-12), 3.75 (s, 3H, IDO-10), 3.38–3.36 (m, 2H, MAL-4), 3.33–3.32 (m, 1H, IDO-11), 3.03 (dd, *J* = 15.3, 8.3 Hz, 1H, IDO-11), 2.65–2.56 (m, 2H, 2× DACH-2), 2.32–2.22 (m, 2H, MAL-8), 2.21–2.13 (m, 1H, DACH-3), 2.10–2.02 (m, 1H, DACH-3), 1.56–1.50 (m, 2H, 2× DACH-4), 1.50–1.39 (m, 6H, MAL-5, MAL-7-, 2× DACH-3), 1.24–1.12 (m, 3H, MAL-6, 1× DACH-4), 1.10–1.00 (m, 1H, DACH-4). ^13^C NMR (151 MHz, DMSO-*d*_6_): δ 180.8 (MAL-10), 174.1 (IDO-13), 171.1 (2× MAL-2), 163.7, 163.7 (2× oxalate), 157.9, 157.7 (2× TFA), 136.8 (IDO-9), 134.5 (MAL-3), 129.0 (IDO-2), 127.2 (IDO-4), 121.4 (IDO-7), 118.7 (IDO-6), 118.4 (IDO-5), 109.9 (IDO-8), 106.7 (IDO-3), 61.5, 60.4 (2× DACH-2), 53.2 (IDO-12), 36.9 (MAL-4), 35.4 (MAL-8), 32.4 (IDO-10), 31.0, 30.8 (2× DACH-3), 27.7 (MAL-5), 26.7 (IDO-11), 25.6 (MAL-6), 24.8 (MAL-7), 23.6, 23.5 (2× DACH-4). MS (*m*/*z*): calcd C_30_H_40_N_5_O_10_Pt (M + H)^+^, 825.24; found, 825.16. EA calcd C_30_H_39_N_5_O_10_Pt·1.5TFA·0.5H_2_O: C, 39.45; H, 4.16; N, 6.97%. Found: C, 39.63; H, 4.11; N, 7.11.

##### (*OC*-6-34)-[(*R*)-2-Ammonio-3-(1-methyl-1*H*-indol-3-yl)propanoato][(1*R*,2*R*)-1,2-cyclohexanediamino][5-(2,5-dioxo-2,5-dihydro-1*H*-pyrrolidin-1-yl)pentylcarbamato]oxalatoplatinum(IV) Trifluoroacetate (**SucCa/IdoEs**)

**4** (30 mg, 0.09 mmol) was dissolved in DMF (2 mL), and TEA (20 μL, 0.14 mmol, 1.5 equiv) and TBTU (33 mg, 0.10 mmol, 1.1 equiv) were added. After 10 min at RT, **8b** (60 mg, 0.09 mmol, 1 equiv) was added and stirred further at RT for 24 h. The solvent was evaporated under reduced pressure, and the crude product was purified by preparative RP-HPLC [43% MeCN (+0.1% HCOOH) in H_2_O (+0.1% HCOOH); isocratic] to obtain ***Boc*-SucCa/IdoEs** (38 mg, 43%) as a yellow solid. Deprotection was performed according to general procedure B starting from ***Boc*-SucCa/IdoEs** (31 mg, 0.03 mmol). Preparative RP-HPLC conditions: 30% MeCN (+0.1% TFA) in H_2_O (+0.1% TFA); isocratic. Yield: 17 mg (51%) of yellow solid. ^1^H NMR (600 MHz, DMSO-*d*_6_; for NMR numbering, see Scheme S1): δ 9.93–9.47 (m, 1H, DACH-1), 8.82–8.62 (m, 1H, DACH-1), 8.09 (br s, 1H, DACH-1), 8.02 (br s, 3H, IDO-14), 7.67–7.56 (m, 2H, 1× DACH-1, IDO-5), 7.44 (d, *J* = 8.3 Hz, 1H, IDO-8), 7.21–7.17 (m, 1H, IDO-7), 7.14 (s, 1H, IDO-2), 7.08 (t, *J* = 7.4 Hz, 1H, IDO-6), 6.87 (t, *J* = 5.5 Hz, 1H, SUC-9), 4.18–4.07 (m, 1H, IDO-12), 3.76 (s, 3H, IDO-10), 3.34–3.31 (m, 3H, SUC-4, 1× IDO-11), 3.03 (dd, *J* = 15.2, 8.2 Hz, 1H, IDO-11), 2.96–2.84 (m, 2H, SUC-8), 2.68–2.58 (m, 6H, SUC-3, 2× DACH-2), 2.19 (d, *J* = 11.2 Hz, 1H, DACH-3), 2.10 (d, *J* = 9.8 Hz, 1H, DACH-3), 1.54 (d, *J* = 11.3 Hz, 2H, 2× DACH-4), 1.49–1.27 (m, 6H, SUC-5, 2× DACH-3, SUC-7), 1.23–1.02 (m, 4H, 2× DACH-4, SUC-6). ^13^C NMR (151 MHz, DMSO-*d*_6_): δ 177.8 (SUC-2), 174.1 (IDO-13), 164.2 (SUC-10), 163.7, 163.7 (2× oxalate), 136.8 (IDO-9), 129.0 (IDO-2), 127.2 (IDO-4), 121.4 (IDO-7), 118.7 (IDO-6), 118.4 (IDO-5), 109.8 (IDO-8), 106.7 (IDO-3), 61.4, 60.3 (2× DACH-2), 53.1 (IDO-12), 40.7 (SUC-8), 37.8 (SUC-4), 32.4 (IDO-10), 31.0, 30.8 (2× DACH-3), 29.1 (SUC-7), 28.0 (SUC-3), 26.9 (SUC-5), 26.8 (IDO-11), 23.6, 23.6 (2× DACH-4), 23.5 (SUC-6). MS (*m*/*z*): calcd C_30_H_42_N_6_O_10_Pt (M + H)^+^, 842.27; found, 842.28. EA calcd C_30_H_42_N_6_O_10_Pt·1.5TFA·0.5H_2_O: C, 38.87; H, 4.20; N, 8.24. Found: C, 38.66; H, 4.45; N, 8.25.

##### (*OC*-6-24)-[(*R*)-(1-Carboxy-2-(1-methyl-1*H*-indol-3-yl)ethyl)carbamato][(1*R*,2*R*)-1,2-cyclohexanediamino][(5-(2,5-dioxopyrrolidin-1-yl)pentyl)carbamato]oxalatoplatinum(IV) (**SucCa/IdoCa**)

In a dry flask, **9** (27 mg; 0.04 mmol) was dissolved in anh. DMF (1 mL), and **6b** (12 mg; 0.04 mmol; 1.5 equiv) was added. The reaction mixture was stirred under Ar at RT for 18 h. The solvent was evaporated *in vacuo* to obtain crude ***t*-butyl SucCa/IdoCa** (39 mg) which was used without further purification. Deprotection was performed according to general procedure B. Preparative RP-HPLC conditions: 30% MeCN (+0.1% TFA) in H_2_O (+0.1% TFA); isocratic. Yield: 15 mg (41% over two steps) of yellow solid. ^1^H NMR (600 MHz, DMSO-*d*_6_; for NMR numbering, see Scheme S1): δ 12.59 (br s, 1H, IDO–COOH), 9.88–9.49 (m, 1H, DACH-1), 9.29 (br s, 1H, DACH-1), 8.56 (br s, 1H, DACH-1), 8.32 (br s, 1H, DACH-1), 7.49 (d, *J* = 7.9 Hz, 1H, IDO-5), 7.36 (d, *J* = 8.2 Hz, 1H, IDO-8), 7.15–7.10 (m, 1H, IDO-7), 7.07 (s, 1H, IDO-2), 7.01 (t, *J* = 7.3 Hz, 1H, IDO-6), 6.84–6.60 (m, 1H, SUC-9), 6.60–6.07 (m, 1H, IDO-14), 4.32–4.12 (m, 1H, IDO-12), 3.73 (s, 3H, IDO-10), 3.32–3.29 (m, 2H, SUC-4), 3.08 (dd, *J* = 14.6, 5.1 Hz, 1H, IDO-11), 3.03 (dd, *J* = 14.6, 7.7 Hz, 1H, IDO-11), 2.95–2.78 (m, 2H, SUC-8), 2.61 (s, 4H, SUC-3), 2.58–2.53 (m, 2H, DACH-2), 2.18–2.08 (m, 2H, 2× DACH-3), 1.54–1.46 (m, 2H, 2× DACH-2), 1.44–1.25 (m, 6H, SUC-5, SUC-7, 2× DACH-3), 1.19–1.06 (m, 4H, 2× DACH-4, SUC-6). ^13^C NMR (151 MHz, DMSO-*d*_6_): δ 177.8 (SUC-2), 173.8 (IDO-13), 164.4 (SUC-10), 163.8 (IDO-15), 163.3 (oxalate), 136.4 (IDO-9), 128.0 (IDO-2), 127.5 (IDO-4), 121.0 (IDO-7), 118.5 (IDO-6), 118.4 (IDO-5), 109.6 (IDO-8), 109.1 (IDO-3), 61.0, 60.9 (2× DACH-2), 55.2 (IDO-12), 40.7 (SUC-8), 37.8 (SUC-4), 32.3 (IDO-10), 30.9 (2× DACH-3), 29.1 (SUC-7), 28.0 (SUC-3), 26.9 (SUC-5), 26.5 (IDO-11), 23.6 (SUC-6), 23.5 (2× DACH-4). MS (*m*/*z*): calcd C_31_H_41_N_6_O_12_Pt (M – H)^−^, 884.24; found, 884.40. EA calcd C_31_H_42_N_6_O_12_Pt·0.5TFA·0.5H_2_O: C, 40.38; H, 4.61; N, 8.83. Found: C, 40.14; H, 4.36; N, 8.69.

##### (*OC*-6-34)-[(*R*)-(1-Carboxy-2-(1-methyl-1*H*-indol-3-yl)ethyl)carbamato][(1*R*,2*R*)-1,2-cyclohexanediamino][6-(2,5-dioxopyrrolidin-1-yl)hexanoato]]oxalatoplatinum(IV) (**SucEs/IdoCa**)

**5b** (9 mg, 0.04 mmol, 1.6 equiv) was dissolved in DMF (0.75 mL). Subsequently, DIPEA (12 μL, 0.07 mmol, 2.5 equiv) and TBTU (17 mg, 0.04 mmol, 1.7 equiv) were added. After 15 min of stirring at RT, **9** (20 mg, 0.03 mmol, 1 equiv) was added and stirred further at RT for 17 h. The solvent was evaporated under reduced pressure, and the crude product was purified by preparative RP-HPLC [44% MeCN (+0.1% HCOOH) in H_2_O (+0.1% HCOOH); isocratic] to obtain ***t*-butyl SucEs/IdoCa** (15 mg, 56%) as a yellow solid. Deprotection was performed according to general procedure B starting from ***t*-butyl SucEs/IdoCa** (75 mg, 0.08 mmol). Preparative RP-HPLC conditions: 28% MeCN (+0.1% HCOOH) in H_2_O (+0.1% HCOOH); isocratic. Yield: 40 mg (55%) of yellow solid. ^1^H NMR (600 MHz, DMSO-*d*_6_; for NMR numbering, see Scheme S1): δ 12.60 (s, 1H, IDO–COOH), 9.26 (t, *J* = 9.3 Hz, 1H, DACH-1), 8.47 (br s, *J* = 4.9 Hz, 1H, DACH-1), 8.32 (br s, 1H, DACH-1), 8.24 (t, *J* = 9.6 Hz, 1H, DACH-1), 7.49 (d, *J* = 7.9 Hz, 1H, IDO-5), 7.36 (d, *J* = 8.2 Hz, 1H, IDO-8), 7.15–7.10 (m, 1H, IDO-7), 7.07 (s, 1H, IDO-2), 7.01 (t, *J* = 7.4 Hz, 1H, IDO-6), 6.58 (d, *J* = 7.9 Hz, 1H, IDO-14), 4.32–4.14 (m, 1H, IDO-12), 3.73 (s, 3H, IDO-10), 3.32–3.29 (m, 2H, ido-N-4), 3.08 (dd, *J* = 14.7, 5.0 Hz, 1H, IDO-11), 3.02 (dd, *J* = 14.8, 7.8 Hz, 1H, IDO-11), 2.60 (s, 4H, SUC-3), 2.57–2.52 (m, 2H, DACH-2), 2.28–2.18 (m, 2H, SUC-8), 2.16–2.05 (m, 2H, 2× DACH-3), 1.54–1.46 (m, 2H, 2× DACH-4), 1.46–1.36 (m, 5H, SUC-5, SUC-7, 1× DACH-3), 1.36–1.26 (m, 1H, 1× DACH-3), 1.20–1.05 (m, 4H, 2× DACH-4, SUC-6). ^13^C NMR (151 MHz, DMSO-*d*_6_): δ 180.9 (SUC-10), 177.8 (2× SUC-1), 173.9 (IDO-13), 163.8 (IDO-15), 163.4, 163.4 (2× oxalate), 136.5 (IDO-9), 128.1 (IDO-2), 127.6 (IDO-4), 121.1 (IDO-7), 118.6 (IDO-6), 118.4 (IDO-5), 109.6 (IDO-8), 109.1 (IDO-3), 61.2, 60.9 (2× DACH-2), 55.2 (IDO-12), 37.7 (SUC-4), 35.5 (SUC-8), 32.3 (IDO-10), 31.0, 30.9 (2× DACH-3), 28.0 (SUC-3), 26.9 (SUC-7), 26.6 (IDO-11), 25.7 (SUC-6), 24.9 (SUC-7), 23.6, 23.5 (2× DACH-4). MS (*m*/*z*): calcd C_31_H_41_N_5_O_12_NaPt (M + Na)^+^, 893.23; found, 893.22. EA calcd C_31_H_39_N_5_O_12_Pt·1.5H_2_O: C, 41.47; H, 4.94; N, 7.80. Found: C, 41.53; H, 4.74; N, 7.69.

##### (*OC*-6-44)-[(*R*)-2-Ammonio-3-(1-methyl-1*H*-indol-3-yl)propanoato][(1*R*,2*R*)-1,2-cyclohexanediamino][6-(2,5-dioxopyrrolidin-1-yl)hexanoato]oxalatoplatinum(IV) Trifluoroacetate (**SucEs/IdoEs**)

**4** (81 mg, 0.26 mmol, 1.1 equiv) and **5b** (54 mg, 0.26 mmol, 1.1 equiv) were dissolved in DMF (5 mL). TEA (97 μL, 0.70 mmol, 3 equiv) and TBTU (186 mg, 0.58 mmol, 2.5 equiv) were added subsequently. After 15 min of stirring at RT, **9** (100 mg, 0.23 mmol, 1 equiv) was added and stirred further at RT for 17 h. The solvent was evaporated under reduced pressure, and the crude product was purified by preparative RP-HPLC [45% MeCN (+0.1% HCOOH) in H_2_O (+0.1% HCOOH); isocratic] to obtain ***Boc*-SucEs/IdoEs** (37 mg, 34%) as a yellow solid. Deprotection was performed according to general procedure B starting from ***Boc*-SucEs/IdoEs** (35 mg, 0.04 mmol). Preparative RP-HPLC conditions: 25% MeCN (+0.1% TFA) in H_2_O (+0.1% TFA); isocratic. Yield: 25 mg (65%) of yellow solid. ^1^H NMR (600 MHz, DMSO-*d*_6_; for NMR numbering, see Scheme S1): δ 8.53–8.41 (m, 1H, DACH-1), 8.27 (t, *J* = 9.4 Hz, 1H, DACH-1), 8.21 (br s, 1H, DACH-1), 8.09–7.94 (m, 3H, IDO-14), 7.67–7.54 (m, 2H, DACH-1, IDO-5), 7.43 (d, *J* = 8.3 Hz, 1H, IDO-8), 7.21–7.16 (m, 1H, IDO-7), 7.12 (s, 1H, IDO-2), 7.09–7.04 (m, 1H, IDO-6), 4.18–4.08 (m, 1H, IDO-12), 3.75 (s, 3H, IDO-10), 3.36–3.29 (m, 3H, SUC-4, 1× IDO-11), 3.03 (dd, *J* = 15.3, 8.3 Hz, 1H, IDO-11), 2.66–2.54 (m, 6H, SUC-3, DACH-2), 2.32–2.22 (m, 2H, SUC-8), 2.17 (d, *J* = 10.8 Hz, 1H, DACH-3), 2.11–2.01 (m, 1H, DACH-3), 1.58–1.50 (m, 2H, 2× DACH-4), 1.50–1.39 (m, 6H, SUC-5, SUC-7, 2× DACH-3), 1.23–1.12 (m, 3H, SUC-6, 1× DACH-4), 1.10–1.00 (m, 1H, DACH-4). ^13^C NMR (151 MHz, DMSO-*d*_6_): δ 180.8 (SUC-10), 177.8 (SUC-2), 174.1 (IDO-13), 163.7, 163.7 (2× oxalate), 158.1, 157.9, 157.7, 157.5 (4× TFA), 136.8 (IDO-9), 129.0 (IDO-2), 127.2 (IDO-4), 121.4 (IDO-7), 118.7 (IDO-6), 118.4 (IDO-5), 109.9 (IDO-8), 106.7 (IDO-3), 61.5, 60.4 (2× DACH-2), 53.2 (IDO-12), 37.6 (SUC-4), 35.4 (SUC-8), 32.4 (IDO-10), 31.0, 30.8 (2× DACH-3), 28.0 (SUC-3), 26.9 (SUC-5), 26.7 (IDO-11), 25.6 (SUC-6), 24.9 (SUC-7), 23.6, 23.5 (2× DACH-4). MS (*m*/*z*): calcd C_30_H_42_N_5_O_10_Pt (M + H)^+^, 827.26; found, 827.23. EA calcd C_30_H_41_N_5_O_10_Pt·1.5TFA·0.5H_2_O: C, 39.37; H, 4.35; N, 6.96. Found: C, 39.37; H, 4.38; N, 7.19.

##### (*OC*-6-34)-Acetato[(1*R*,2*R*)-1,2-cyclohexanediamine]oxalato[(5-(2,5-dioxopyrrolidin-1-yl)pentyl)carbamato]platinum(IV) (**SucCa/OAc**)

In a dry flask, **6b** (67 mg, 0.32 mmol, 1.5 equiv) was dissolved in anh. DMF (3 mL), and **7b** (97 mg, 0.21 mmol, 1 equiv) was added. The reaction mixture was stirred under Ar at RT for 20 h. The solvent was removed under reduced pressure, and the residue was taken up in MeOH and precipitated with Et_2_O. The crude product was purified *via* preparative RP-HPLC [15% MeCN (+0.1% HCOOH) in H_2_O (+0.1% HCOOH); isocratic] to obtain **SucCa/OAc** (30 mg; 21%) as a white solid. ^1^H NMR (500 MHz, DMSO-*d*_6_; for NMR numbering, see Scheme S1): δ 10.02–9.43 (m, 1H, DACH-1), 8.81–7.95 (m, 3H, DACH-1), 6.87–6.21 (m, 1H, SUC-9), 3.31–3.28 (m, 2H, SUC-4), 2.95–2.77 (m, 2H, SUC-8), 2.61 (s, 4H, SUC-3), 2.60–2.53 (m, 2H, DACH-2), 2.13 (d, *J* = 8.8 Hz, 2H, DACH-3), 1.95 (s, 3H, acetate), 1.51 (d, *J* = 8.9 Hz, 2H, DACH-4), 1.47–1.25 (m, 6H, SUC-5, 2× DACH-3, SUC-7), 1.21–1.04 (m, 4H, 2× DACH-4, SUC-6). ^13^C NMR (126 MHz, DMSO-*d*_6_): δ 178.4 (acetate–C=O), 177.8 (SUC-2), 164.4 (SUC-10), 163.4, 163.3 (2× oxalate), 61.2, 60.9 (2× DACH-2), 40.6 (SUC-8), 37.8 (SUC-4), 31.0, 30.8 (2× DACH-3), 29.1 (SUC-7), 28.0 (SUC-3), 26.9 (SUC-5), 23.6 (SUC-6), 23.5 (DACH-4), 22.9 (acetate–CH_3_). MS (*m*/*z*): calcd C_20_H_30_N_4_O_10_Pt (M + H)^+^, 684.18; found, 684.18. EA calcd C_20_H_32_N_4_O_10_Pt·H_2_O: C, 34.24; H, 4.88; N, 7.99. Found: C, 34.47; H, 4.80; N, 8.28.

##### (*OC*-6-44)-Acetato[(1*R*,2*R*)-1,2-cyclohexanediamino][3-(1-methyl-1*H*-indol-3-yl)propanoato]oxalatoplatinum(IV) (**OAc/IPAEs**)

**IPA** (11 mg, 0.06 mmol, 1.1 equiv) was dissolved in DMF (1 mL), and TEA (10.4 μL, 0.06 mmol, 1.5 equiv) and TBTU (20 mg; 0.06 mmol; 1.3 equiv) were added. After 10 min at RT, **6b** (24 mg, 0.05 mmol, 1 equiv) was added and stirred further at RT for 16 h. The solvent was evaporated under reduced pressure, and the crude product was purified by preparative RP-HPLC [31% MeCN (+0.1% HCOOH) in H_2_O (+0.1% HCOOH) isocratic] to obtain **OAc/IPAEs** (7 mg; 20%) as a white solid. ^1^H NMR (500 MHz, DMSO-*d*_6_; for NMR numbering, see Scheme S1): δ 8.31 (br s, 4H, DACH-1), 7.48 (d, *J* = 7.9 Hz, 1H, IDO-5), 7.35 (d, *J* = 8.2 Hz, 1H, IDO-8), 7.12 (t, *J* = 7.6 Hz, 1H, IDO-7), 7.03 (s, 1H, IDO-2), 7.00 (t, *J* = 7.4 Hz, 1H, IDO-6), 3.70 (s, 3H, IDO-10), 2.87 (t, *J* = 7.5 Hz, 2H, IDO-11), 2.64–2.60 (m, 2H, IDO-12), 2.57–2.53 (m, 1H, DACH-2), 2.44–2.38 (m, 1H, DACH-2), 2.13–2.01 (m, 2H, DACH-3), 1.96 (s, 3H, acetate), 1.49–1.24 (m, 4H, 2× DACH-4, 2× DACH-3), 1.18–1.07 (m, 1H, DACH-4), 1.03–0.93 (m, 1H, DACH-4); ^13^C NMR (126 MHz, DMSO-*d*_6_): δ 180.6 (acetate–C=O), 178.5 (IDO-13), 163.5 (oxalate), 136.6 (IDO-9), 127.2 (IDO-4), 126.6 (IDO-2), 121.1 (IDO-7), 118.4 (IDO-5), 118.3 (IDO-6), 112.8 (IDO-3), 109.5 (IDO-8), 61.2, 61.0 (2× DACH-2), 36.4 (IDO-12), 32.2 (IDO-10), 30.9, 30.8 (2× DACH-3), 23.5, 23.4 (2× DACH-4), 23.0 (acetate–CH_3_), 20.9 (IDO-11). MS (*m*/*z*): calcd C_22_H_28_N_3_O_8_Pt (M – H)^−^, 657.15; found, 657.16.

### UHPLC-Reduction Experiments

The platinum(IV)–succinimide complexes were dissolved in 2% DMF in phosphate buffer (500 mM, pH 7.4) to a final concentration of 2 mM. The solutions were then mixed 1:1 with a 20 mM stock of l-ascorbic acid in phosphate buffer (500 mM, pH 7.4) to obtain final concentrations of 1 mM complex, 10 mM l-ascorbic acid, and 1% DMF. The samples were incubated at 20 °C and measured on a Dionex UltiMate 3000 RS UPLC system with a Waters Acquity UPLC BEH C18 column (3 × 50 mm, pore size 1.7 μm) and absorption detection at 220 nm. Data points were taken at *t* = 0 and every 30 min for 6 h.

### SEC–ICP–MS Studies

FCS was purchased from Sigma-Aldrich and buffered with 150 mM phosphate buffer (pH 7.4) to guarantee a stable pH. The maleimide-bearing platinum(IV) complexes were dissolved in 10% DMF in 150 mM phosphate buffer (pH 7.4) to 1 mM and diluted 1:10 in serum to obtain a final concentration of 100 μM. The samples were then incubated in the autosampler at 37 °C for 24 h and analyzed every 1 h. Between each sample, a pure water blank was measured. For SEC–ICP–MS measurements, an Agilent 1260 Infinity system coupled to an Agilent 7800 ICP–MS equipped with a dynamic reaction cell was used. Oxygen (purity 5.5, Messer Austria GmbH, Gumpoldskirchen, Austria) was used as reaction gas. HPLC parameters are given in Table S4, and ICP–MS operation parameters are given in Table S5.

### Cell Culture

The human SKOV3 (ATCC HTB-77, ovarian adenocarcinoma) and HCT116 (ATCC CCL-247, colorectal carcinoma) cells were maintained in McCoy’s 5A (Sigma-Aldrich, MO, USA). Murine CT26 (ATCC CRL-2638, colon carcinoma) and murine GL261 (glioblastoma, kindly provided by T. Felzmann, Vienna, Austria) were kept in Dulbecco’s modified Eagle’s medium (DMEM)/F12 (1:1). Human OVCAR (ATCC HTB-161, ovarian adenocarcinoma), murine AB12 (mesothelioma), murine AE17 (mesothelioma), and the human melanoma (VM1, VM7, and VM15, established at our institute^47^) cancer cell lines were maintained in RPMI-1640. ID8 (ovarian adenocarcinoma, kindly provided by F. Roby, Kansas, USA) was kept in DMEM (supplemented with 5 μg/mL insulin, 5 μg/mL transferrin, and 5 ng/mL Na_2_SeO_3_), and murine K7M2 (ATCC CRL-2836, osteosarcoma) was kept in DMEM medium. All media were supplemented with 10% FBS (PAA, Linz, Austria) in a humidified atmosphere at 37 °C and 5% CO_2_.

### Cytotoxicity Assay

The cells were seeded at 3500–6000 cells/well in 96-well plates depending on the proliferation rate of the respective cell line and allowed to recover for 24 h. Subsequently, the cells were treated at indicated concentrations for 48 or 72 h. Cells were additionally incubated with 5-fold equimolar concentrations of ascorbic acid to increase the reduction rate of the compounds. Cell viability was measured by the MTT-based vitality assay (EZ4U; Biomedica, Vienna, Austria), following the manufacturer’s recommendations. Full dose–response curves were generated using GraphPad Prism software to calculate IC_50_ values (drug concentrations at 50% reduced cell viability compared to control).

### pH2AX Signal Detection and Quantification

HCT116 cells were seeded on polytetrafluoroethylene-printed spot slides (E63424-06, Science Services GmbH, Munich, Germany) at a density of 4000 cells/spot. On the next day, cells were exposed to the compounds for 24 h, at 50 μM and fixed in a 4% formaldehyde solution in phosphate-buffered saline (PBS) with a pH adjusted to 7.4 (158127, Merck, Darmstadt, Germany) for 15 min at RT. Spots were washed thrice with PBS each for 5 min, and samples were then incubated in a blocking buffer [PBS, 5% albumin (Carl Roth, Karlsruhe, Germany), 0.3% Triton X-100 (T8787, Merck, Darmstadt, Germany)] for 1 h at RT. Next, samples were incubated with a primary antibody solution of phospho-histone H2A.X (Ser139) (clone 20E3, #9718, Cell Signaling, Danvers, USA) at a dilution of 1:200 in an antibody dilution buffer [PBS with 1% albumin (Carl Roth, Karlsruhe, Germany) and 0.3% Triton X-100 (T8787, Merck, Darmstadt, Germany)] for 1 h at RT. Subsequently, samples were washed twice with PBS (5 min each), incubated with goat anti-rabbit antibody linked to Alexa Fluor 488 (A-11008, Thermo Fisher Scientific, Massachusetts, USA), and diluted 1:500 in antibody dilution buffer for 1 h at RT. Next, samples were incubated with rhodamine-labeled wheat germ agglutinin (RL-1022, Vector Laboratories, California, USA) at a concentration for 5 μg/mL for 15 min at RT, and spots were washed twice in PBS, embedded in vectashield mounting medium with DAPI (VECH-1200, Szabo-Scandic, Vienna, Austria), and analyzed using a LSM700 confocal microscope (Zeiss Axio Observer.Z1, inverse, 63× plan-apochromat NA 1.4 Oil DIC objective). Per condition, five representative images were taken (16-bit, 101.61 × 101.61 μm). Images were analyzed using ImageJ software. Nuclear area was selected by applying default threshold to the DAPI channel of each image. Then, Alexa Fluor 488 signal (integrated density) was measured and normalized to nuclear area.

### Cell Cycle Analysis

The cells were seeded at 600,000 cells/well in six-well plates and allowed to recover for 24 h. On the next day, cells were exposed to the compounds for 24 h, then trypsinized, and collected. Subsequently, cells were washed with PBS and centrifuged (400*g*, 5 min), and the cell pellet was resuspended in 100 μL of 0.9% NaCl solution. The cell suspension was then added dropwise to 1.8 mL of ice-cold 80% ethanol and incubated for 48 h at −20 °C. The fixated cells were centrifuged (6200*g*, 2 min), and the cell pellet was diluted in 0.5 mL of PBS with 0.1 mg/mL RNAse A (R5503, Merck, Darmstadt, Germany), which was previously heat-activated (10 min at 96 °C), and samples were incubated for 30 min at 37 °C to remove RNA content. Next, to stain DNA, propidium iodide (81845, Merck, Darmstadt, Germany) at a concentration of 5 μg/mL was added, samples were incubated for 30 min on ice and subsequently measured by flow cytometry.

### ICP–MS Measurements of HCT116 Cells and Tumor Tissues

HCT116 cells were seeded at 6 × 10^5^ cells/well in a six-well plate and allowed to settle for 24 h. Cells were exposed to drugs at 20 μM for 3 h under normal cell culture conditions. Cells were washed twice with PBS and lysed at RT in 500 μL HNO_3_ (≥69%, Rotipuran Supra, Carl Roth, Karlsruhe, Germany) for 1 h. 400 μL of the lysate was transferred to 7.6 mL of ultrapure water (18.2 MΩ cm, Milli-Q Advantage, Darmstadt, Germany).

For tissue digestion, approx. 25–50 mg of tissue was weighed into perfluoralkoxy tubes, and 2 mL of HNO_3_ and 100 μL H_2_O_2_ (30%, Suprapur, Merck, Darmstadt, Germany) were added. The solutions were placed on a hot plate and heated up for 7 h using a temperature program with a maximum of 200 °C. The liquids were transferred to 15 mL tubes with the remaining solution being removed by washing twice with 4 mL of ultrapure water.

Platinum concentrations were measured using an Agilent 7800 ICP-QMS instrument (Agilent Technologies, Tokyo, Japan) equipped with an Agilent SPS 4 autosampler (Agilent Technologies, Tokyo, Japan) and a MicroMist nebulizer at a sample uptake rate of approx. 0.2 mL/min. The Agilent MassHunter software package (Workstation Software, Version C.01.04, 2018) was used for data evaluation. All of the measured samples were blank corrected (for the cell culture data, wells containing no cells were used). The instrumental parameters for the ICP–MS are summarized in Table S6. Elemental standard solutions were purchased from Labkings (Hilversum, The Netherlands). The instrument was tuned on a daily basis.

### log *D*_7.4_ Determination by ICP–MS

50 mM stock solutions of the succinimide complexes in DMF were diluted in phosphate buffer (20 mM, pH 7.4, pre-saturated with *n*-octanol) to a final concentration of 50 μM drug and 0.1% DMF. The platinum content of these aqueous solutions was measured by ICP–MS (see above). The solutions were mixed 1:1 with *n*-octanol and shaken on a 360° rotatable rack for 3 h. The solutions were centrifuged for 5 min (860*g*, RT), the aqueous layers were carefully removed, and their Pt content was measured by ICP–MS. Experiments were performed and measured in duplicates, and averages were used for further calculations. log *D*_7.4_ values were calculated by the following equation
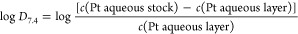


### RNA Isolation and Detection of IDO Levels by Real-Time PCR

Total RNA was isolated from cells using the TRIzol reagent (15596-026, Thermo Fisher Scientific, Massachusetts, USA), according to the manufacturer’s instructions. RNA was reverse transcribed to complementary DNA (cDNA) using RevertAid Reverse Transcriptase (EP0441, Thermo Fisher Scientific, Massachusetts, USA). The real-time PCR method is described elsewhere.^58^ In short, cDNA samples were diluted 1:25 and analyzed using Maxima SYBR Green/ROX qPCR Master Mix (2×) (K0221, Thermo Fisher Scientific, Massachusetts, USA) in a CFX96 Touch real-time PCR detection system (Bio-Rad Laboratories, California, USA) for their specific mRNA content using the following primer sequences purchased from Eurofins Genomics: human β-actin: fwd (5′-GGA TGC AGA AGG AGA TCA CTG-3′), rev (5′-CGA TCC ACA CGG AGT ACT TG-3′); human IDO: fwd (5′-GTC ATG GAG ATG TCC GTA AG-3′), rev (5′-CTT GGA GAG TTG GCA GTA AG-3′); murine β-actin: fwd (5′-ATG GAG GGG AAT ACA GCC-3′), rev (5′-TTC TTT GCA GCT CCT TCG TT-3′); murine IDO transcript 1: fwd (5′-ATG TTG GCT TTG CTC TAC CA-3′), rev (5′-GAC CAC CTC AGG GTT TAG CG-3′).

### Long-Term Cytotoxicity Assay

SKOV3 cells were seeded in duplicates at 5000 cells/well in 24-well plates and allowed to recover for 24 h. Subsequently, the cells were treated at the indicated concentrations for 168 h. Cells were fixed with ice-cold methanol (20 min at 4 °C), washed with PBS, and stained with crystal violet. Subsequently, colony formation was inspected, and the fluorescence signal was detected using Typhoon Trio imager (GE Healthcare, Little Chalfont, UK) at 610/30 nm BP and analyzed by ImageJ software. Fluorescence values of empty wells were subtracted from each value, and full dose–response curves were generated to calculate IC_50_ values (drug concentrations at 50% reduced fluorescence intensity compared to control).

### Reduction and 1-MDT Release in SKOV3 Lysates

SKOV3 cells were grown in four T225 mm^2^ cell culture flasks to reach a high cell number. On the day of cell lysate isolation, cells were washed with PBS, scraped from the surface, and centrifuged. Each cell pellet was lysed in ∼160 μL lysis buffer (15 mM NaCl 0.5% Triton X, ultrapure) and incubated on ice for 1 h. The samples were then centrifuged for 15 min at 14,000 rpm (4 °C), and the cleared lysate was pooled and kept at −80 °C until analysis. Cell lysates were treated with 2 mM stock solutions of respective complexes in 4% DMF and phosphate buffer (100 mM, pH 7.4) to obtain a final compound concentration of 100 μM and incubated at 37 °C. After 0, 4, 24, and 72 h, three volume equivalents of cold MeOH were added to sample aliquots. Samples were stored at 4 °C for 10 min and centrifuged (15,080*g*, 4 °C, 10 min). LC–MS analysis of the supernatant was performed on an Agilent 1260 Infinity system using a Waters Atlantis T3 column (150 mm × 4.6 mm) and absorption detection at 230 nm, coupled to a Bruker amaZon SL ESI-IT mass spectrometer. Milli-Q water, containing 0.1% formic acid, and acetonitrile, containing 0.1% formic acid, were used as eluents. A gradient of 1–99% acetonitrile in 20 min was used.

### Colorimetric Kyn Assay

SKOV3 cells were seeded at 5000 cells/well in 96-well plates and allowed to recover for 24 h. Cells were treated for 72 h. Supernatants (200 μL/well) and blank medium were transferred to microcentrifuge tubes, and cell viability was measured by MTT assay. To precipitate proteins, the supernatants were mixed with 100 μL 30% (w/v) trichloroacetic acid. *N*-Formyl-Kyn was hydrolyzed to Kyn by incubating the supernatants for 30 min in a thermoblock at 50 °C and 300 rpm. The supernatants were cleared by centrifugation (10 min at 10,000*g*), and 100 μL of the clear supernatants was transferred to a fresh 96-well plate in duplicates and incubated with 100 μL Ehrlich’s reagent [2% 4-(dimethylamino)benzaldehyde (Sigma-Aldrich, D2004, MO, USA) in acetic acid (Merck, Darmstadt, Germany)] for 10 min at RT. For the washout experiments, cells were seeded in quintuplicates. 72 h after treatment, the cell supernatant was exchanged with serum-free medium. After 24 h incubation, the supernatant was analyzed, as described above. The absorption of Kyn was measured at 490 nm (reference 620 nm), and the absorbance of blank medium was subtracted. The Kyn concentrations were calculated using a standard curve [generated from l-Kyn (Sigma-Aldrich, K8625, MO, USA) dilution series in blank medium] and normalized to cell viability.

### Animals

8–12 week old Balb/c and SCID mice (Harlan Laboratories, San Pietro Al Natisone, Italy) were kept in a pathogen-free environment. Every procedure was carried out under sterile conditions and according to the regulations of the Ethics Committee for the Care and Use of Laboratory Animals at the Medical University Vienna.

### Organ Distribution in SKOV3-Bearing SCID Mice

SKOV3 cells (1.5 × 10^6^ cells in 50 μL serum-free medium) were injected subcutaneously into the right flank of male SCID mice. When the tumors reached a volume of at least ∼340 mm^3^, the mice (*n* = 2 per group) were treated once with the compounds iv at concentrations equimolar to 9 mg/kg oxaliplatin (**MalEs/IdoCa** 19.7 mg/kg, **MalCa/IdoCa** 20.5 mg/kg, **MalEs/IdoEs** 21.4 mg/kg, and **MalCa/IdoEs** 21.5 mg/kg) in 20% propylene glycol (PG). **OAc/OAc** (12.1 mg/kg) was applied in 0.9% NaCl and oxaliplatin (9.0 mg/kg) in 5% glucose. After 24 h, the animals were anesthetized and blood drawn by heart puncture. Then, the animals were sacrificed by cervical dislocation, and tumors and organs were collected for the measurement with ICP–MS. For plasma isolation, blood was collected in ethylenediaminetetraacetic acid (EDTA)-coated tubes and centrifuged for 10 min at 900*g* at 4 °C. Plasma was transferred to a new tube and centrifuged once more to remove residual red blood cells.

### Detection of Trp and Its Metabolites in Cell Culture and in Tumor Tissue by LC–HRMS

#### Sample Preparation

The cell supernatant was analyzed using adapted protocols from Simón-Manso *et al.*(59) SKOV3 cells were seeded at 5000 cells/well in a 96-well plate. 24 h after seeding, cells were treated for 72 h. The medium was exchanged to serum-free medium, and cells were incubated for 24 h. To 50 μL of cell supernatant or blank medium, 50 μL of fully ^13^C internal standard (labeled yeast extract prepared in 2 mL of H_2_O; ISOtopic solutions e.U., Vienna, Austria) and 400 μL of MeOH (LC–MS grade, Sigma-Aldrich, Vienna, Austria) were added for protein precipitation (−20 °C overnight) and centrifugation (14,000*g*, 4 °C, 15 min). 100 μL of aliquots was dried for direct LC–HRMS analysis.

To 50 mg of tumor tissue, 50 μL of fully ^13^C internal standard (labeled yeast extract prepared in 2 mL of H_2_O; ISOtopic solutions e.U., Vienna, Austria) and 950 μL of 80% MeOH (LC–MS grade, Sigma-Aldrich, Vienna, Austria) were added and homogenized in a Dounce-type glass tissue grinder. The homogenate was collected, the tissue homogenizer was washed twice with 400 μL 80% MeOH (LC–MS grade, Sigma-Aldrich, Vienna, Austria), and the washing solution was added to the homogenate. The samples were vortexed, centrifuged (14,000*g*, 4 °C, 20 min), and kept on dry ice until further processing. 500 μL of tumor sample aliquots was dried for direct LC–HRMS analysis.

#### LC–HRMS Analysis

All samples were resuspended in 100 μL of water (supernatant sample: same volume; tumor samples 5-fold concentration) and measured using a 12 min method based on reversed-phase chromatography (column: HSS T3, 1.8 μm, 2.1 × 150 mm, Waters) and high-resolution mass spectrometry (Orbitrap HF, Thermo). For quantification, multi-point calibration with external standard mixes (Kyn, Trp, and kynurenic acid) using co-eluting ^13^C internal standards was performed and analyzed by Skyline 20.2.0.286. For 1-MDT, relative quantification based on ^13^C Trp normalization was performed.

### Anticancer Activity Experiment

CT26 cells (5 × 10^5^ cells in 50 μL of serum-free medium) were injected subcutaneously into the right flank of Balb/c mice. On day 3, when the tumors were palpable, the mice (*n* = 4 per group) were treated with the compounds iv at concentrations equimolar to 9 mg/kg oxaliplatin (**MalEs/IdoEs** 23 mg/kg in 10% PG, **MalCa/IdoEs** 22.2 mg/kg in 15% PG, **MalCa/IdoCa** 20.6 mg/kg in 15% PG, and **MalEs/IdoCa** 21 mg/kg in 15% PG/PBS) twice a week (Mon/Thu or Tue/Fri) for 2 weeks. Every day, the animals were monitored for body weight and the enhanced stress level, and the tumor size was measured using a caliper according to the formula length × width^2^/2.

### Sample Preparation of Murine Tissue for Flow Cytometry

CT26 cells (5 × 10^5^ cells in 50 μL of serum-free RPMI medium) were injected into the right flank of female Balb/c mice (*n* = 4 per group). On day 7, when the tumors reached a mean tumor volume of ∼170 mm^3^, mice were treated once iv with **MalEs/IdoCa** at a concentration equimolar to 9 mg/kg (dissolved in 20% PG in PBS) or solvent only. After 24 h, the animals were sacrificed, and tumor tissues and the tumor-draining lymph nodes were isolated. Tissues were collected in a tube with 750 μL of ice-cold PBS containing 5% FBS and cut into small pieces. Samples were digested using PBS containing 50 mg/mL collagenase (C2139, Merck, Darmstadt, Germany) and 50 mg/mL DNase I (DN25, Darmstadt, Germany) and filtered several times through a 70 μm cell strainer (734-2761, VWR, Pennsylvania, USA). The samples were centrifuged, and cell pellets were treated with ammonium–chloride–potassium (ACK)-buffer (150 mM NH_4_Cl, 10 mM KHCO_3_, 127 μM EDTA, pH 7.2–7.4) to lyse red blood cells and centrifuged in a Würzburg buffer (PBS containing 5% FBS, 5 mM EDTA, 20 μg/mL DNase I).

### Human PBMC Isolation

Blood was drawn from healthy donors and collected in EDTA-coated tubes. Blood was diluted in PBS–2 mM EDTA (Titriplex III, Merck, Darmstadt, Germany) 1:2 and carefully layered on top of Ficoll-Paque solution (Merck, Darmstadt, Germany) in a 50 mL falcon tube. Subsequently, blood was centrifuged for 40 min, at 400*g*, at 20 °C without active deceleration to obtain a buffy coat layer containing PBMC. Upper layer, containing serum, was aspirated, and the buffy coat layer was collected and washed in PBS containing 2 mM EDTA. PBMCs were diluted in RPMI-1640 supplemented with 10% FBS. For later analysis, PBMCs were frozen in RPMI-1640–40% FBS–15% DMSO and stored in liquid nitrogen.

### Immune Co-culture and Sample Preparation for Flow Cytometry

PBMCs were thawed and slowly dropped into warm medium at a rate of 1 drop/5 s. PBMCs were then centrifuged (10 min, 400*g*) and washed with warm AIM V medium (12055091, Thermo Fisher Scientific, Massachusetts, USA). PBMCs were co-cultured with SKOV3 at a ratio of 2:1 (PBMC/SKOV3) in 12-well CytoOne plates (CC7682-7512, Starlab, Hamburg, Germany). In the presence of IL-2 (SRP3085, Merck, Darmstadt, Germany) at a concentration of 80 ng per 10^6^, PBMCs were added. To induce T_reg_ differentiation, OKT3 monoclonal CD3 antibody (317302, BioLegend, California, USA) and TGF-β1 CHO (AF-100-21C-10 μg, Eubio, Vienna, Austria) were added at concentrations of 4 μg and 10 ng per 10^6^ PBMC, respectively. Cells were incubated with drugs at nontoxic concentrations of 10 μM for oxaliplatin and 50 μM for prodrugs. After 3 days, 250 μL of fresh AIM V medium was added, and cells were incubated for another two days. On the day of measurement, supernatant, containing PBMC, was collected.

### Flow Cytometry

Cells were washed with PBS and stained using the Aqua Zombie Fixable Viability Kit (423101, BioLegend, California, USA), according to the manufacturer’s instructions. Cells were then stained with fluorescent dye-labeled antibodies, according to the manufacturer’s instructions (Table S7). For nuclear FOXP3 staining, cells were fixated using a True-Nuclear Transcription Factor Buffer Set (424401, BioLegend, California, USA), in accordance with manufacturer’s instructions. Single stains for each antibody using the AbC Total Antibody Compensation Bead Kit (A10497, Thermo Fisher) were performed to generate compensation matrices. Sample acquisition was performed using an LSRFortessa X-20 cell analyzer (BD Biosciences, New Jersey, USA). Data analysis was performed using FlowJo software (FlowJo LLC, Oregon, USA) after processing with the flowAI plugin to remove unwanted events resulting from changes in the flow rate.^60^ The cleaned up data were analyzed using the gating strategy, as shown in Figure S15 for murine tissue and Figure S16 for human co-culture experiment.

### Statistical Analysis

All data are presented as mean ± SD. Comparison between groups was analyzed by unpaired two-tailed Student’s *t*-test or multi comparison (ANOVA) with Dunnett’s or Bonferroni post-hoc-test. Statistical analysis was performed using Prism 8 (GraphPad) (*p < 0.05, **p < 0.01, ***p < 0.001, and ****p < 0.0001).

## Supporting Information Available

^1^H and ^13^C NMR spectra and HPLC traces of final compounds, stability data, reduction kinetics, additional cytotoxicity data, DNA damage induction, cell cycle analysis, Kyn levels in supernatant, 1-MDT levels in tumor tissue, and additional *in vivo* data (PDF)
Molecular formula strings (CSV)

## Notes

^¶^ P.H. and C.R.K. have shared last authorship.

## Abbreviations

1-MDT: 1-methyl-d-tryptophan
1-MLT: 1-methyl-l-tryptophan
1-MT: 1-methyltryptophan
DIPEA: *N*,*N*-diisopropylethylamine
EPR: enhanced permeability and retention
FCS: fetal calf serum
ICP-MS: inductively coupled plasma mass spectrometry
IDO: indoleamine 2,3-dioxygenase
**IPA**: 3-(1-methyl-1*H*-indol-3-yl)propanoic acid
Kyn: kynurenine
LC–HRMS: liquid chromatography–high resolution mass spectrometry
MeCN: acetonitrile
MeOH: methanol
MTT: 3-(4,5-dimethylthiazol-2-yl)-2,5-diphenyltetrazolium bromide
OAc: acetate
PBMCs: peripheral blood mononuclear cells
PBS: phosphate-buffered saline
PG: propylene glycol
PTFE: polytetrafluoroethylene
SEC–ICP–MS: size exclusion chromatography–inductively coupled plasma mass spectrometry
TBTU: 2-(1*H*-benzotriazole-1-yl)-1,1,3,3-tetramethylaminium tetrafluoroborate
TEA: triethylamine
T_reg_: regulatory T cell
