# Supplementary material for: Structure–Activity Relationships of Triple-Action Platinum(IV) Prodrugs with Albumin-Binding Properties and Immunomodulating Ligands
Source: J Med Chem. 2021 Aug 17;64(16):12132–51. doi: 10.1021/acs.jmedchem.1c00770 (PMC8404199; doi:10.1021/acs.jmedchem.1c00770)
Supplement: Supplementary file 1 — jm1c00770_si_001.pdf [file jm1c00770_si_001.pdf]

Supporting Information

Structure-activity relationships of triple-action platinum(IV) prodrugs with albumin-binding properties and immunomodulating ligands

Philipp Fronik[a#], Isabella Poetsch[abc#], Alexander Kastner[a], Theresa Mendrina[b], Sonja Hager[b], Katharina Hohenwallner[d], Hemma Schueffl[b], Dietmar Herndler-Brandstetter[b], Gunda Koellensperger[d], Evelyn Rampler[d], Joanna Kopecka[e], Chiara Riganti[e], Walter Berger[bc], Bernhard K. Keppler[ac], Petra Heffeter[bc*], and Christian R. Kowol[ac*]

[a] University of Vienna, Faculty of Chemistry, Institute of Inorganic Chemistry, Waehringer Strasse 42, 1090 Vienna, Austria.

[b] Institute of Cancer Research and Comprehensive Cancer Center, Medical University of Vienna, Borschkegasse 8a, 1090 Vienna, Austria.

[c] Research Cluster "Translational Cancer Therapy Research", 1090 Vienna, Austria

[d] University of Vienna, Faculty of Chemistry, Institute of Analytical Chemistry, Waehringer Strasse 38, 1090 Vienna, Austria

[e] University of Torino, Department of Oncology, University of Torino, via Santena 5/bis, 10126, Torino, Italy

[#] These authors have contributed equally

Table of contents

Figures S1-S2:	SEC-ICP-MS supporting information	Page S3-S4
Figures S3-S4:	Stability data of compounds	Page S5
Figures S5-S16:	Biological data	Page S6-S16
Figures S17-S24:	NMR characterization	Page S17-S24
Figures S25-S32:	UHPLC chromatograms	Page S25-S27
Tables S1-S3	Additional IC50 values	Page S28-S29
Table S4	Pt ratio in tissue samples	Page S30
Tables S5-S8:	Materials and methods	Page S31-S33
Scheme S1:	NMR numbering scheme	Page S34

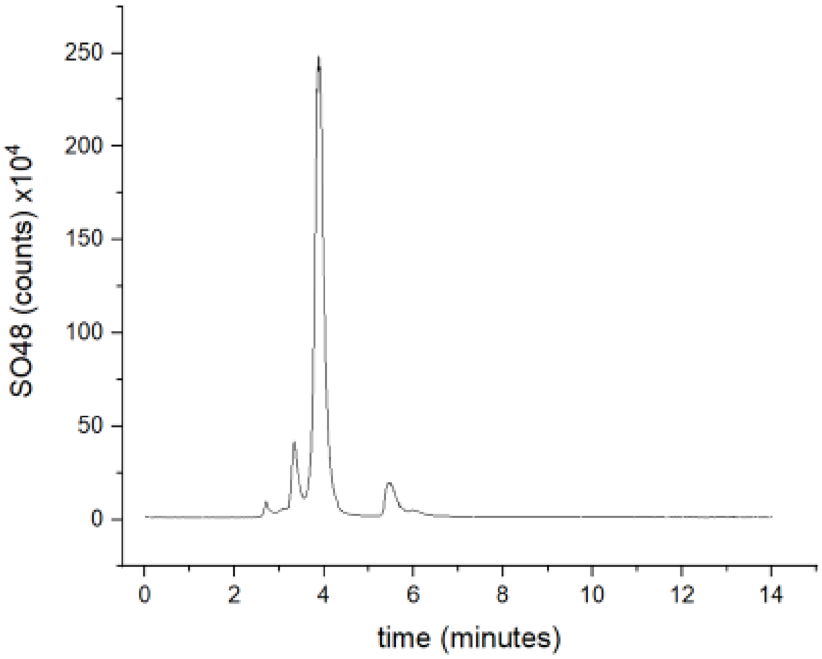

Figure S1: *Sulfur trace of fetal calf serum (+150 mM phosphate buffer, pH 7.4), measured by ICP-MS.*

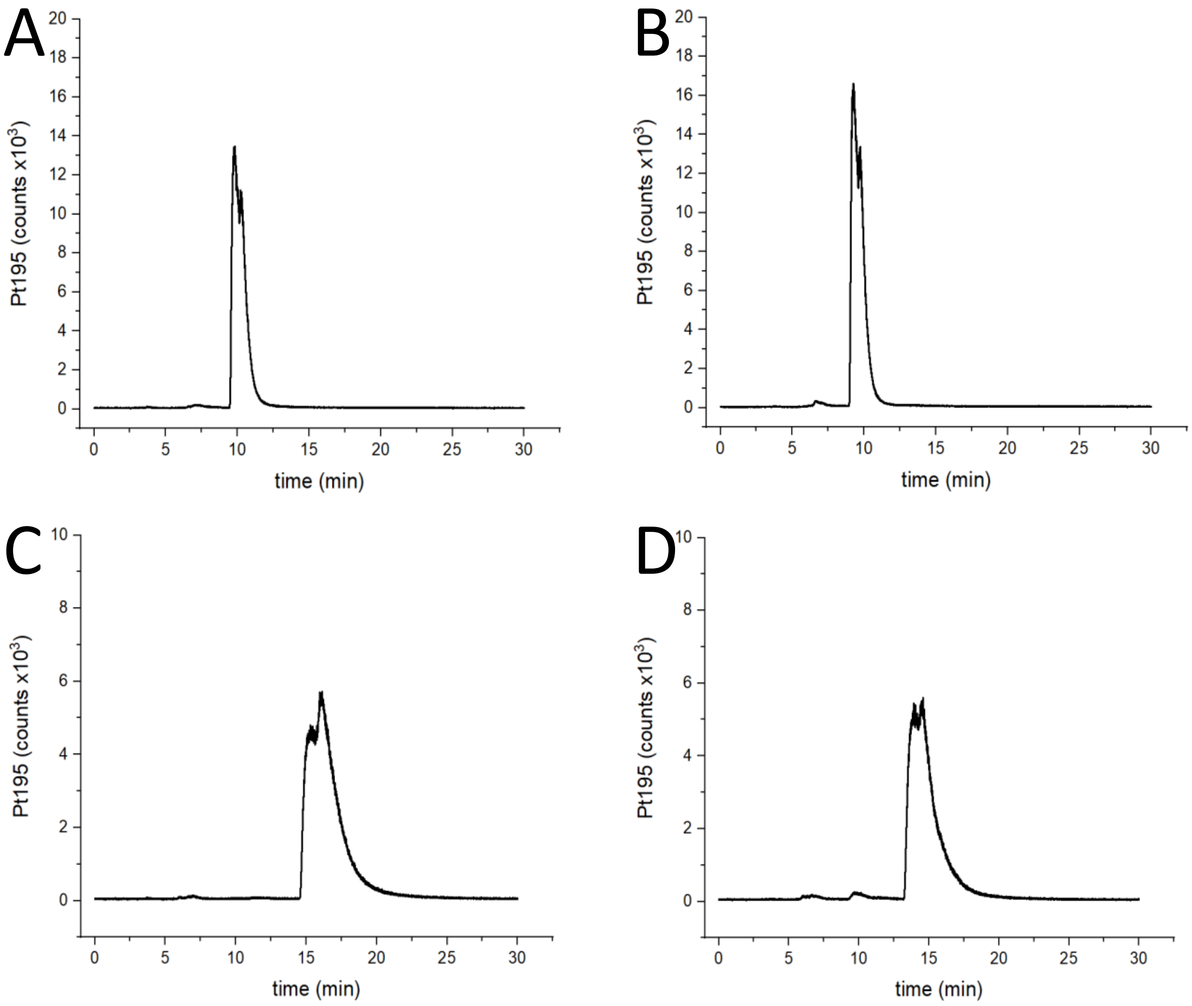

Figure S2: *SEC-ICP-MS traces of complexes* ***(A) SucEs/IdoCa****,* ***(B) SucCa/IdoCa****,* ***(C) SucCa/IdoEs*** *and* ***(D) SucEs/IdoEs*** *after incubation in fetal calf serum (+150 mM phosphate buffer, pH 7.4) at 37°C. No significant binding to albumin (t_R ~4 min) could be observed for any of the compounds.*

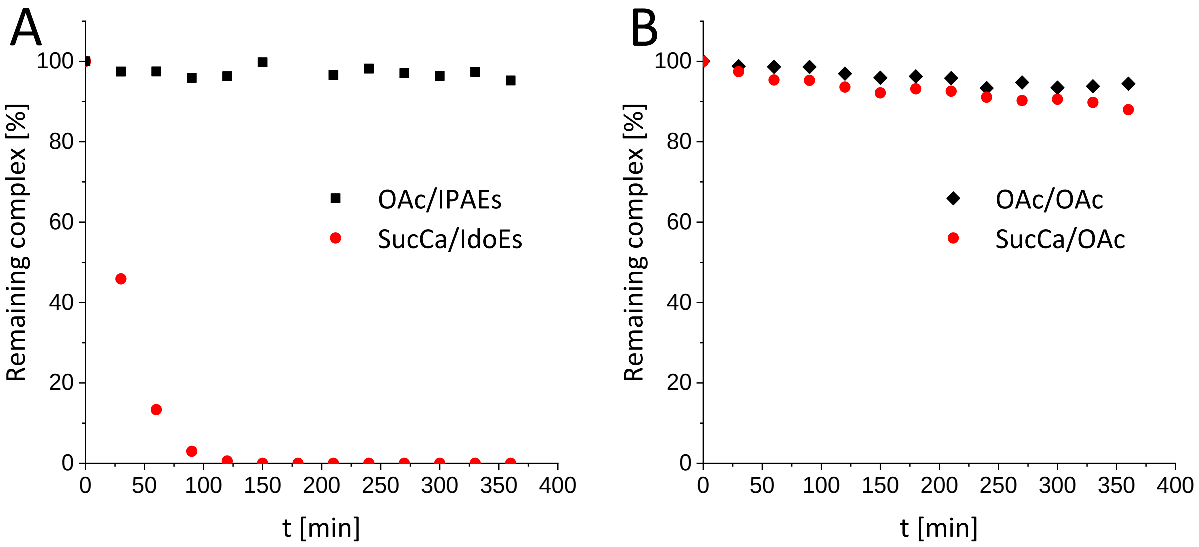

Figure S3: *Reduction kinetics of reference compounds with AA at 20°C, measured by UHPLC and UV absorption at 220 nm. A: Comparison of* ***OAc/IPAEs*** *with amino-group containing* ***SucCa/IdoEs****; B: Reductive stability of reference compounds with no 1MDT-like ligand. AUC-values were normalized to t = 0. Experimental conditions: 1 mM complex, 10 mM AA in 1% DMF and 500 mM phosphate buffer (pH 7.4).*

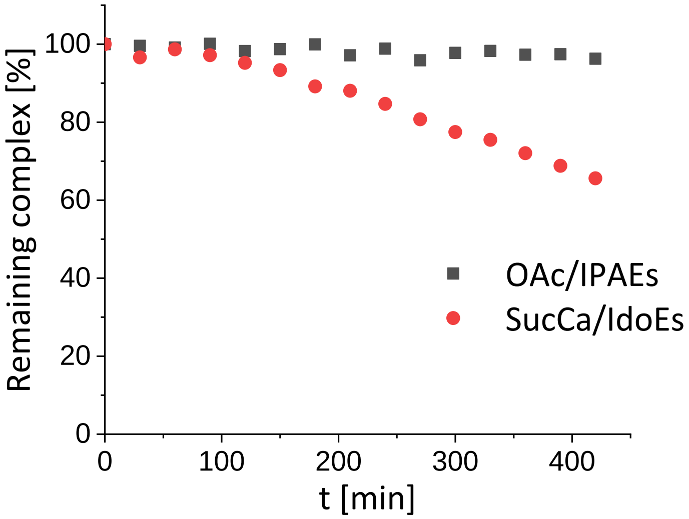

Figure S4: *Reduction kinetics of* ***OAc/IPAEs*** *and* ***SucCa/IdoEs*** *in the presence of dithiothreitol (DTT) at 20°C, measured by UHPLC. The complex* ***OAc/IPAEs*** *does not have a free amino group on its 1-MDT-like ligand and is stable under these conditions, whereas* ***SucCa/IdoEs*** *is readily reduced. Experimental conditions: 1 mM complex, 10 mM DTT in 1% DMF and 500 mM phosphate buffer (pH 7.4).*

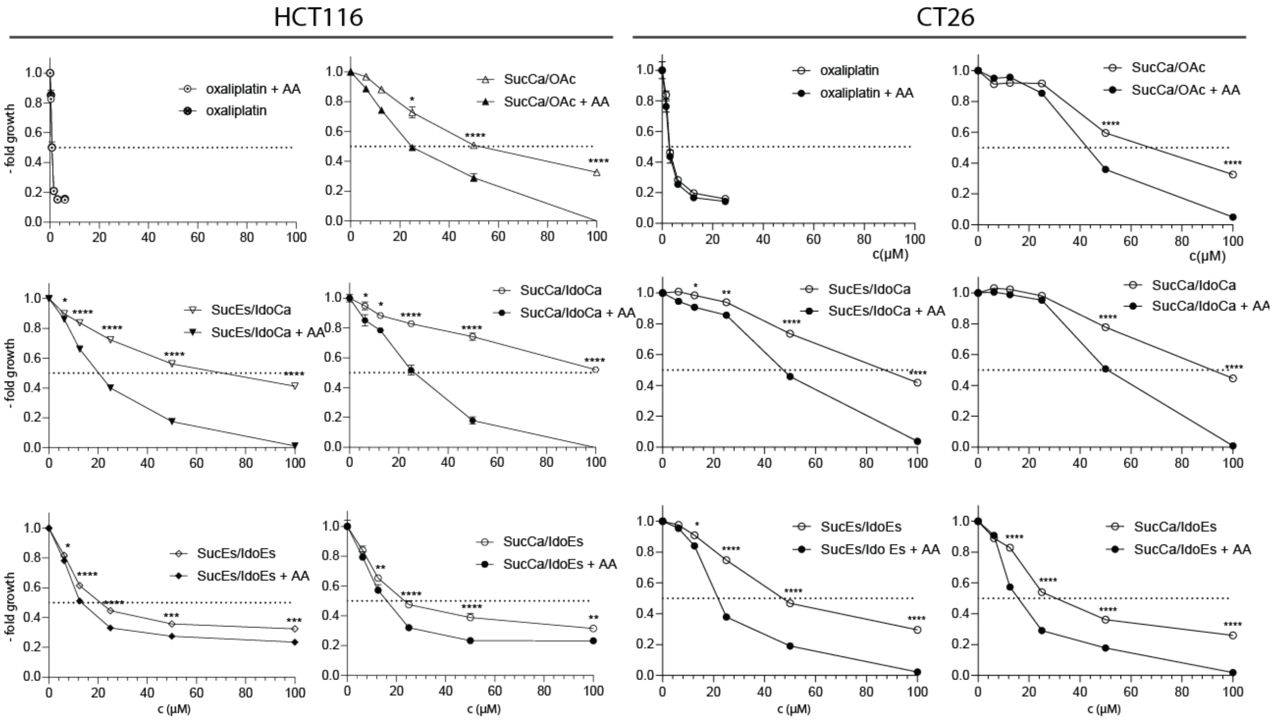

Figure S5: *Cytotoxicity determination. HCT116 or CT26 cancer cells were treated for 72 h with increasing drug concentrations in the presence or absence of 5-fold equimolar amount of AA. The viability of the cells was analyzed by MTT assay. Data shown are mean +/- standard deviation of triplicates from one exemplary experiment. Significance was calculated using multiple comparisons (two-way ANOVA) * p<0.05, ** p<0.01, *** p<0.001, **** p<0.0001.*

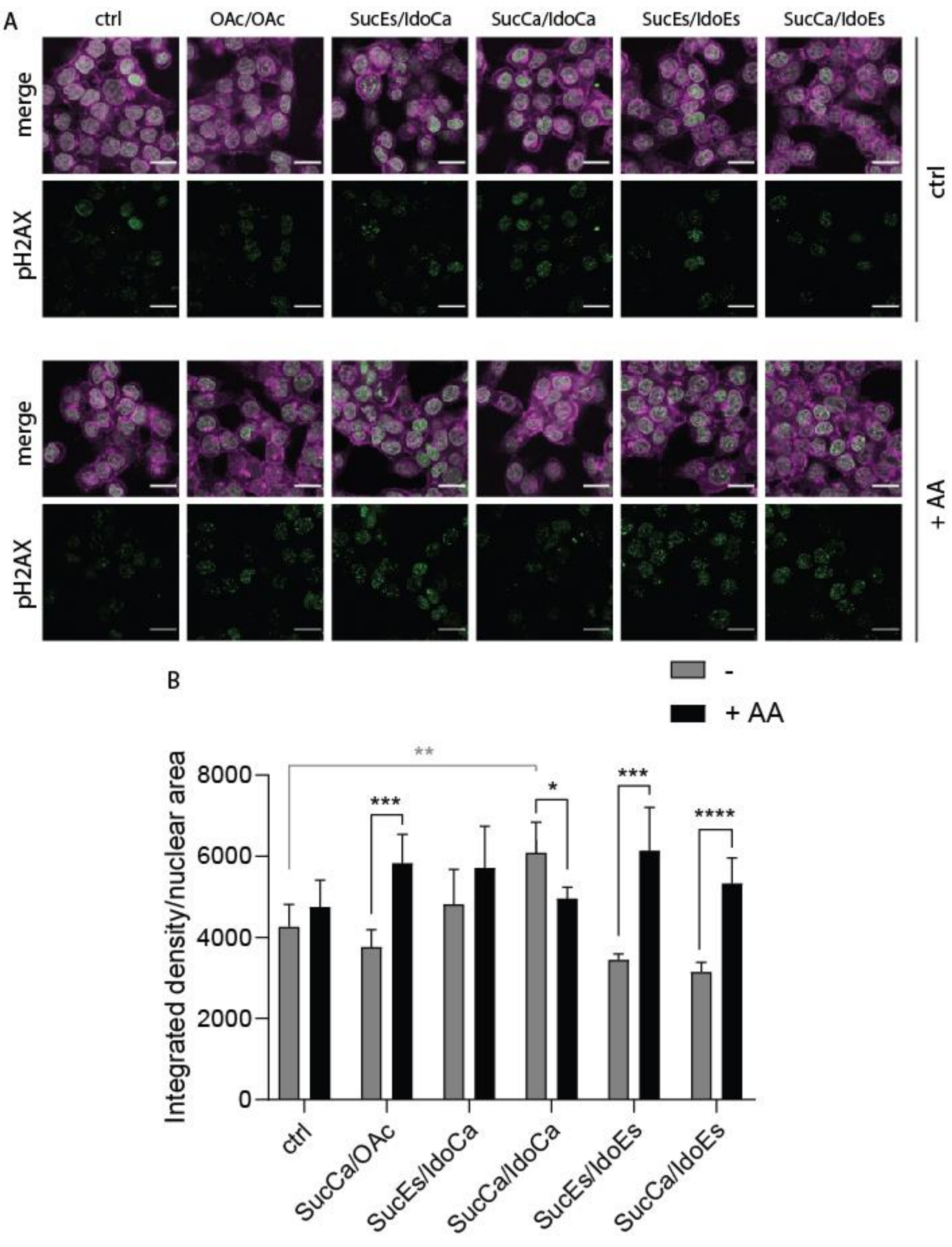

Figure S6: *DNA damage levels. HCT116 cells were treated with the respective drugs at 50 µM for 24 h in the absence or presence of 5-fold excess AA. (A) Cells were fixed and stained with TRITC-phalloidin (magenta), DAPI (white) and pH2AX antibody (green). Shown are representative fluorescence images (scale bar 20 µm). (B) pH2AX fluorescence intensity signals were quantified and are depicted as mean ± SD from five images. Significance compared to control (ctrl) was calculated by mixed-effects analysis and Sidak's multiple comparisons test. Significance comparing conditions with and without AA was calculated by multiple t tests. * p<0.05, ** p<0.01, *** p<0.001, **** p<0.0001*

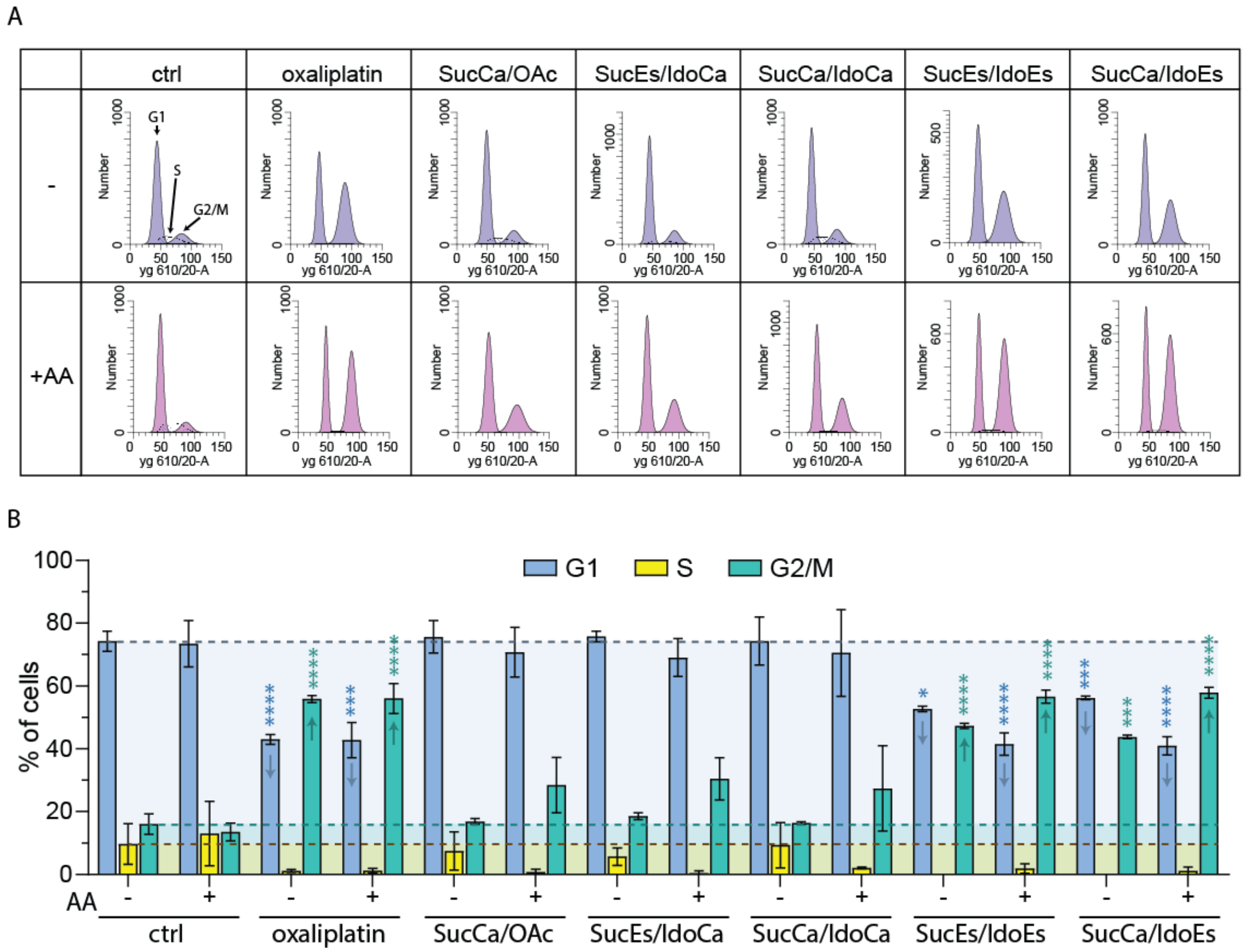

Figure S7: *Cell cycle analysis. HCT116 cells were treated with the respective drugs at 50 µM (10 µM in case of oxaliplatin) for 24 h in the absence or presence of 5-fold excess AA. Cells were analyzed through flow cytometry and cell cycle phases were allocated according to the DNA content. (A) Exemplary histogram of cell cycle distribution. (B) Graph shows percentage of cell cycle distribution. Bars depict mean ± SD from two independent experiments. Statistical significance compared to respective control was calculated by 2way ANOVA and Tukey's multiple comparisons test. * p < 0.05, *** p < 0.001, **** p < 0.0001.*

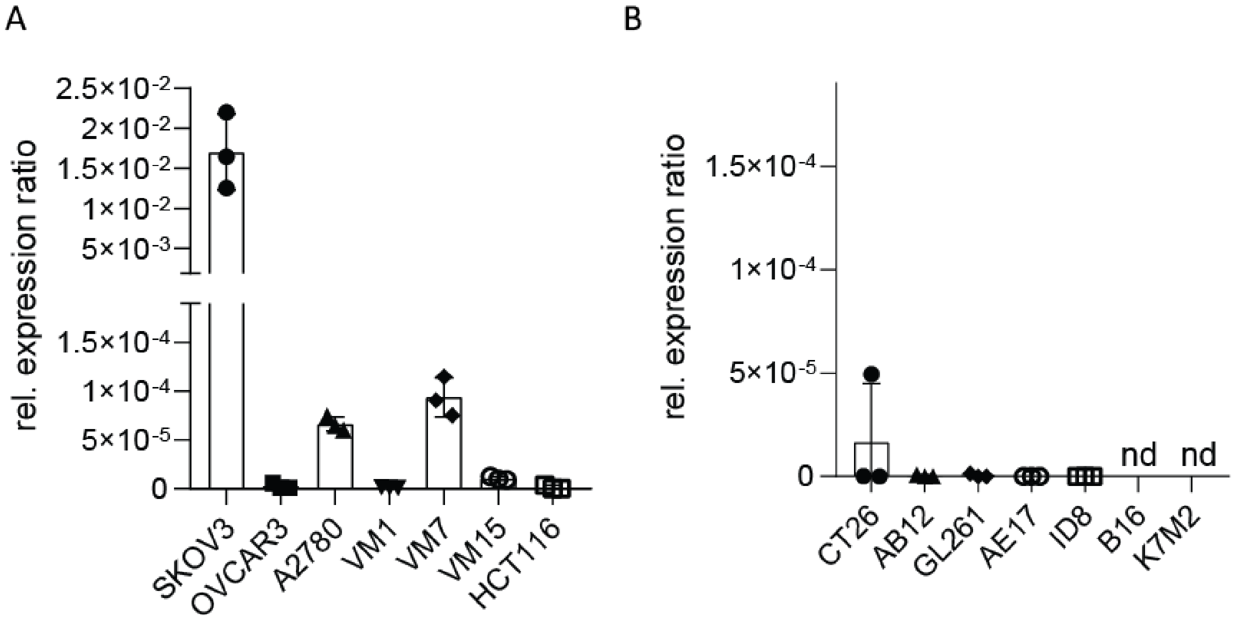

Figure S8: *mRNA levels of IDO transcript in (A) human cell lines or (B) murine cell lines normalized to the mRNA level of the housekeeping gene β-actin.*

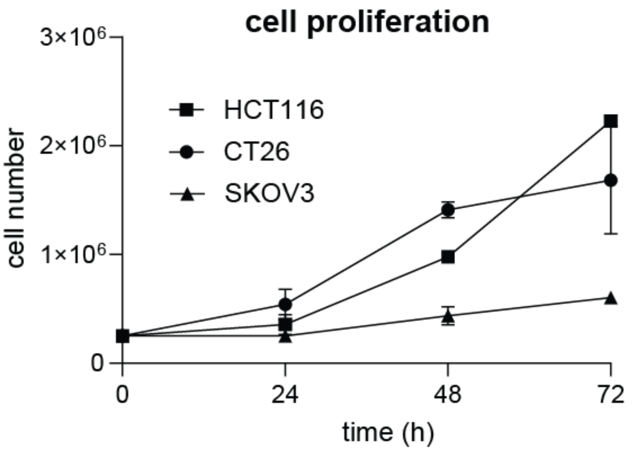

Figure S9: *Proliferation rate of different cancer cell models: 250 000 cells were seeded and counted every 24 h to determine cell proliferation. Data is presented as mean +/- SD from 2 individual experiments.*

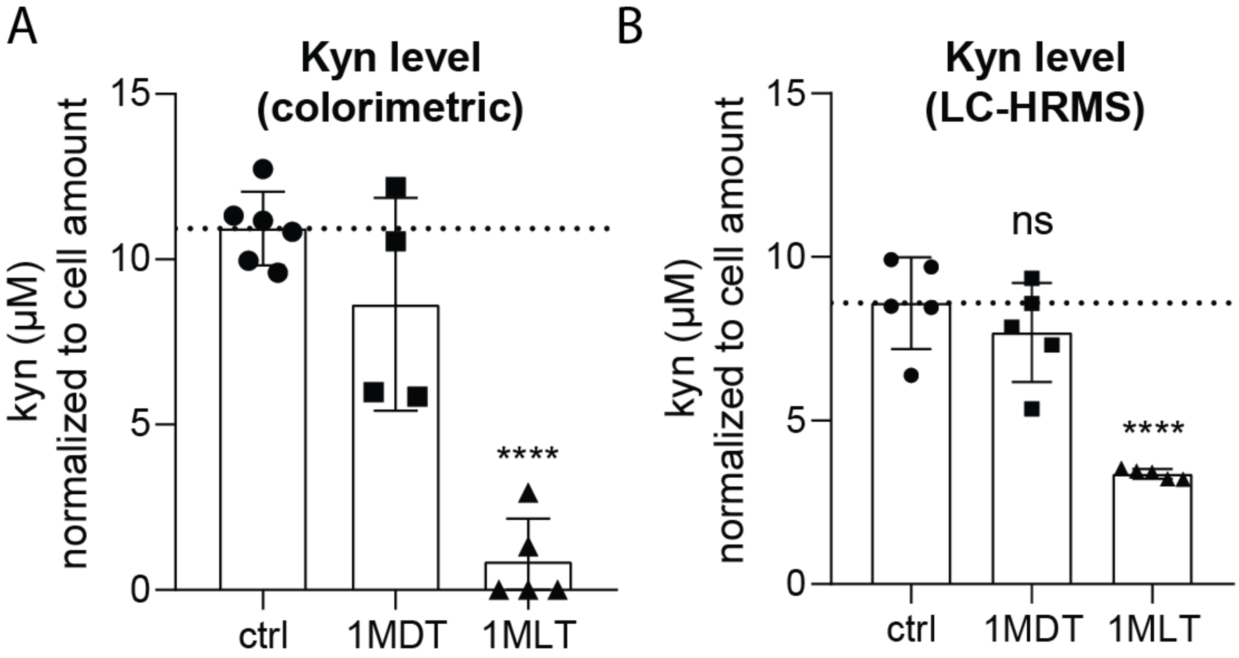

Figure S10: *(A) Colorimetric measurement of Kyn in cell supernatants from SKOV3 cells after 72 h treatment at sub-toxic concentrations (2 mM). Values were normalized to cell amount based on their metabolic capacity (B) LC-HRMS measurement of the supernatants from (A) to detect Kyn levels. Values were normalized to cell amount based on their metabolic capacity. Data is presented as mean +/- SD from 5 replicates. Significance was calculated by multiple comparison analysis (one-way ANOVA) and Dunnett Post-Hoc-test * p<0.05, ** p<0.01, *** p<0.001, **** p<0.0001.*

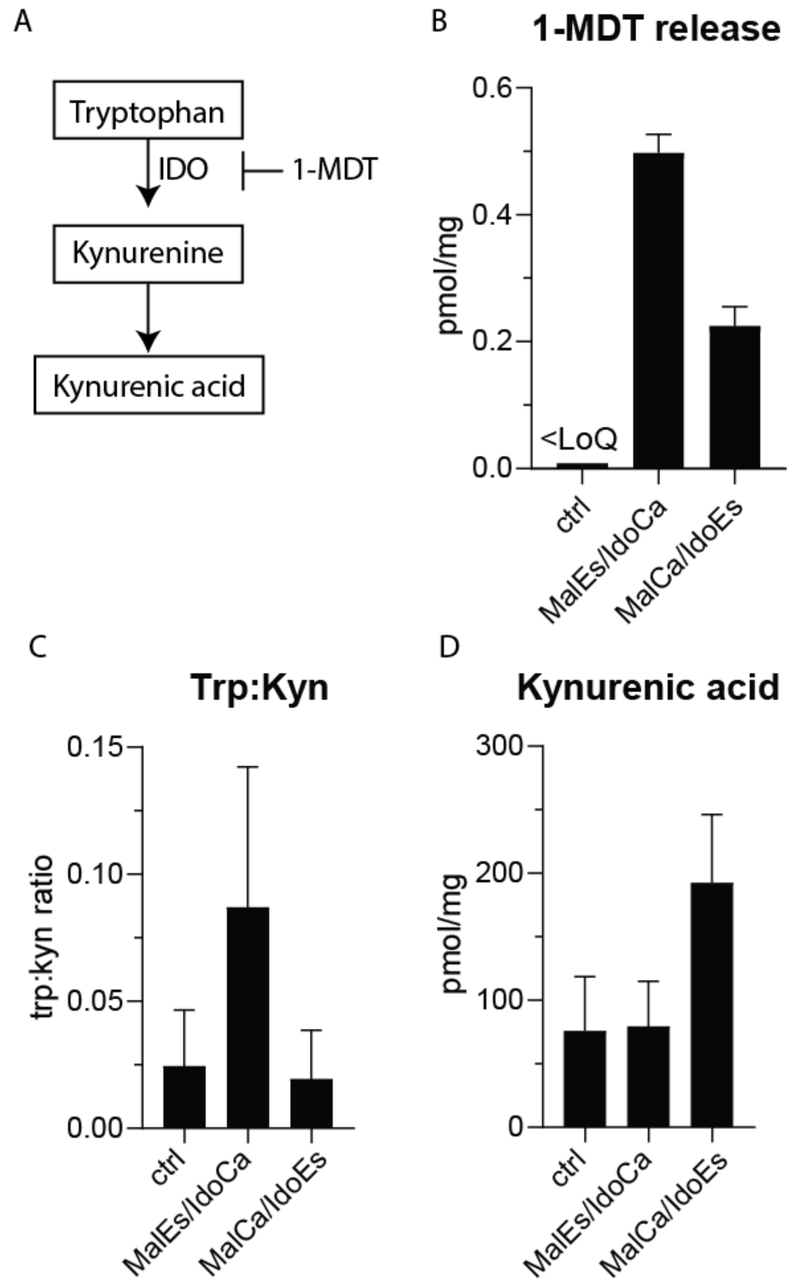

Figure S11: *Inhibition of kynurenine pathway in tumor tissue. (A) Scheme shows downstream catabolites of IDO activity. (B) Relative quantification by LC-HRMS of 1-MDT levels from tumor tissue. (C) Ratio of tryptophan to kynurenine level in tumor tissue. Significance of the quantified metabolites by LC-HRMS was calculated by ordinary one-way ANOVA and Tukey's multiple comparisons test. ** p<0.01, *** p<0.001 (D) Downstream catabolite of IDO activity. Bars indicate mean ± SD from 2 biological replicates.*

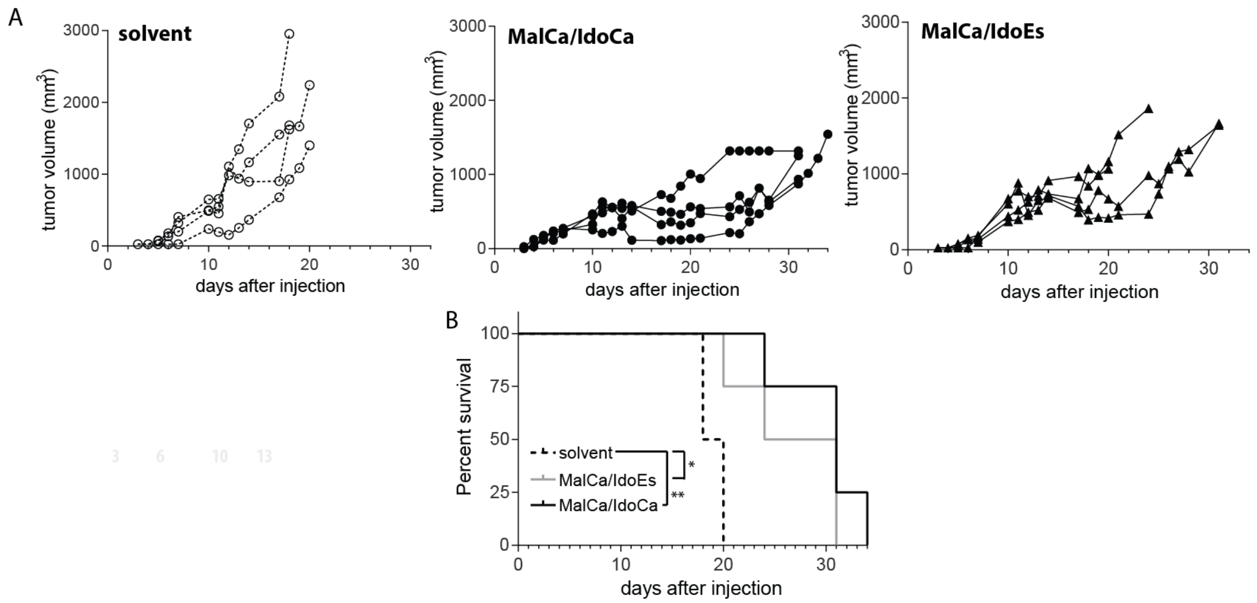

Figure S12: *(A) Therapy of CT26-bearing Balb/c mice treated (i.v.) with* ***MalCa/IdoCa*** *or* ***MalCa/IdoEs*** *at concentrations equimolar to 9 mg/kg oxaliplatin. Tumor growth was measured daily. Each graph shows individual values from one treatment group. (B) Kaplan-Meier blot from (A). Significance was calculated using Log-rank (Mantel-Cox) test.*

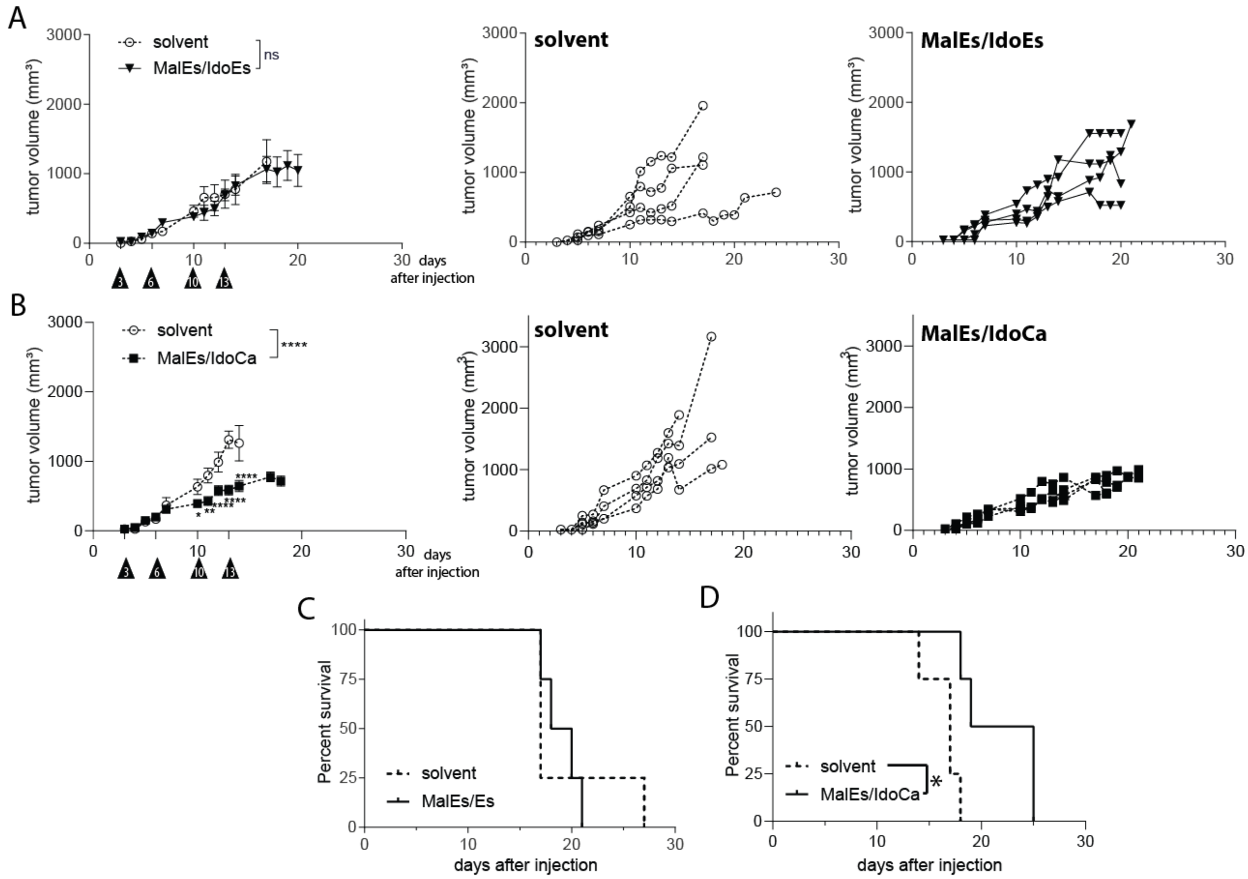

Figure S13: *Therapy of CT26 bearing Balb/c mice treated (i.v.) with **MalEs/IdoEs** (A) or **MalEs/IdoCa** (B) at concentrations equimolar to 9 mg/kg oxaliplatin. Tumor growth was measured daily. Left graph shows mean ± SEM, black arrows indicate treatments. Significance was calculated in comparison to the solvent group and within each group by multiple comparison analysis and 2-way ANOVA, respectively. * p<0.05, ** p<0.01, *** p<0.001, **** p<0.0001. Middle and right graphs show individual values from each one treatment group. (C) and (D) show respective Kaplan-Meier blot from (A) and (B). Significance was calculated using Log-rank (Mantel-Cox) test.*

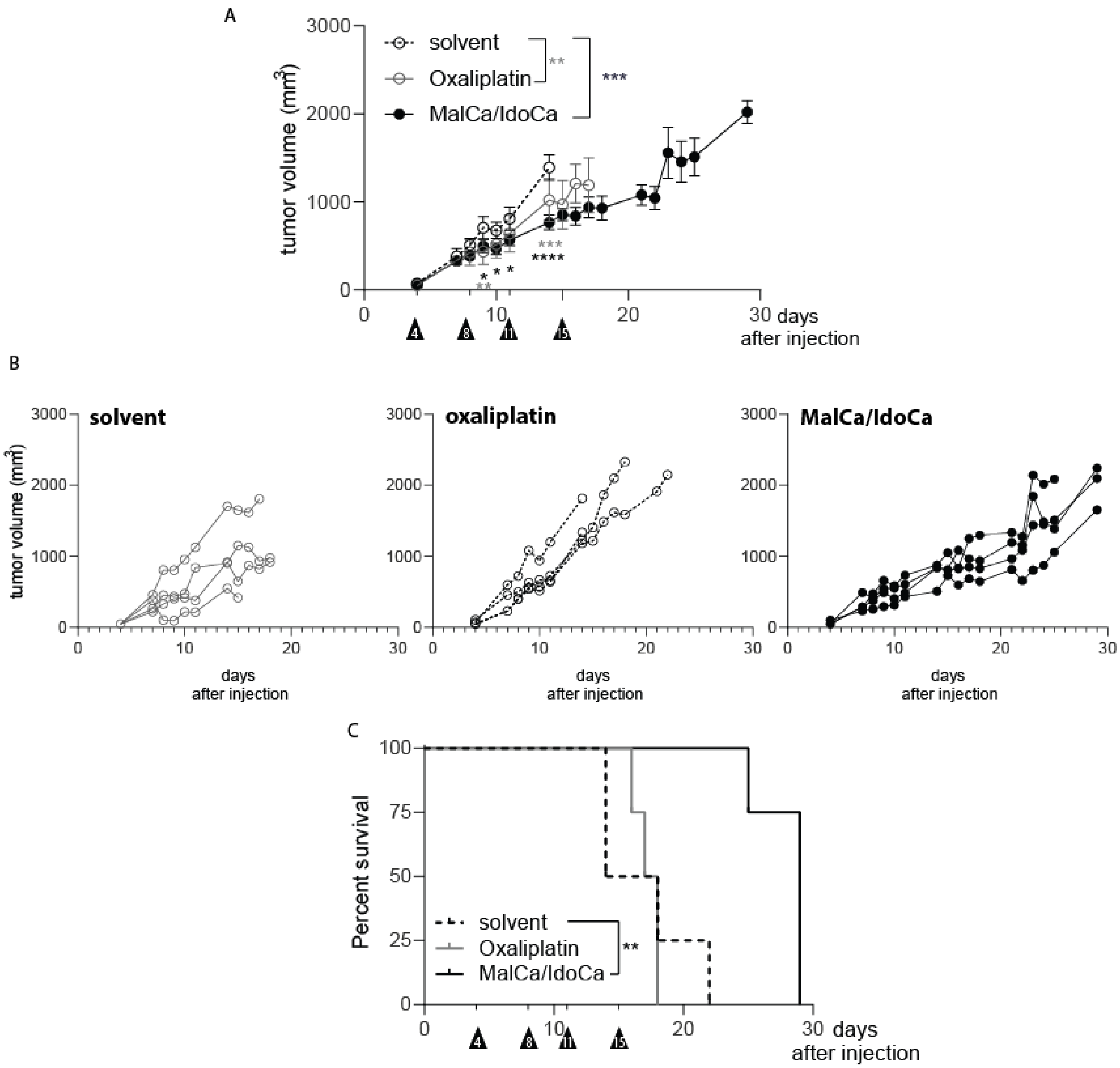

Figure S14: *Therapy of CT26-bearing Balb/c mice treated (i.v.) with* ***oxaliplatin*** *or* ***MalCa/IdoCa*** *at concentrations equimolar to 9 mg/kg oxaliplatin. (A) Tumor growth was measured daily and is shown as mean ± SEM. Black arrows indicate treatments. Significance was calculated in comparison to the solvent group and within each group by multiple comparison analysis and 2-way ANOVA, respectively. Significance between curves was calculated using paired t test. * p<0.05, ** p<0.01, *** p<0.001, **** p<0.0001. (B) shows individual values from (A) from each treatment group. (C) Kaplan-Meier-curve showing survival of the mice. Significance was calculated using Log-rank (Mantel-Cox) test compared to solvent group.*

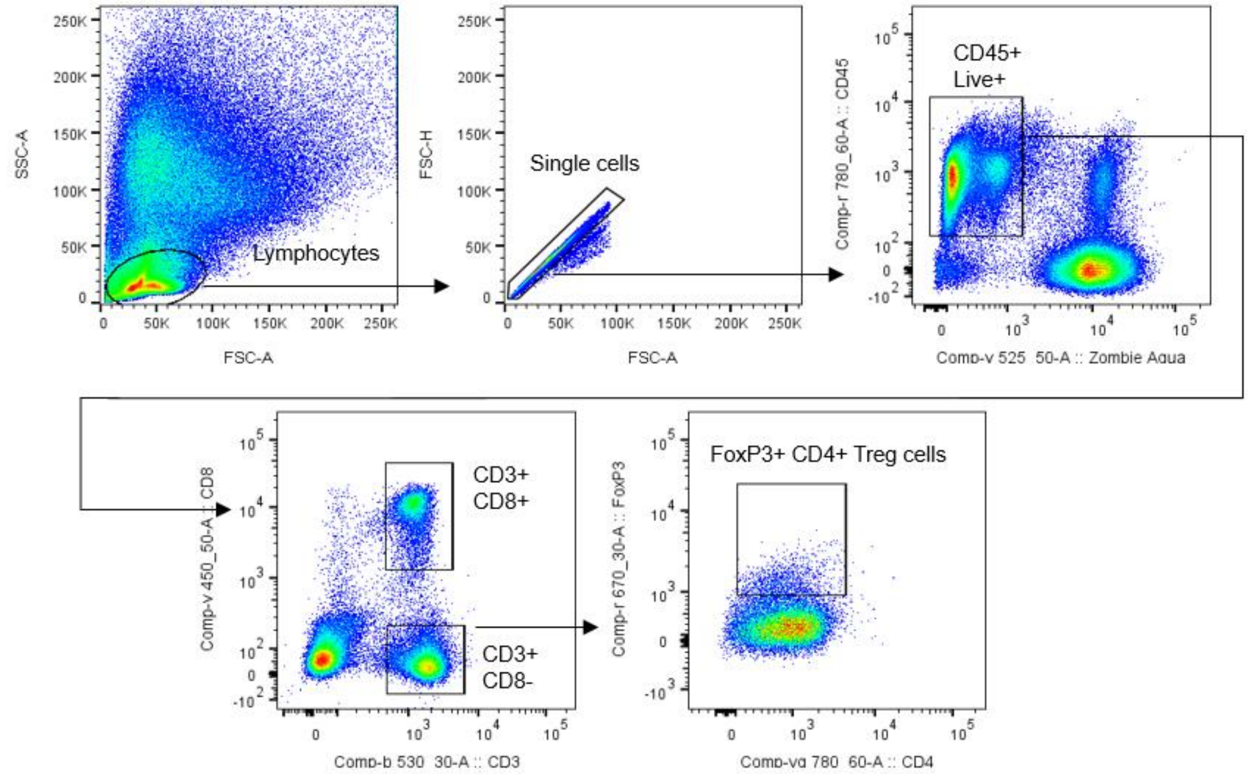

***Figure S15**: Gating strategy of CD8⁺ T cells, CD4⁺ T cells and FoxP3⁺ CD4⁺ Treg from the mouse experiment (tumor-draining lymph nodes).*

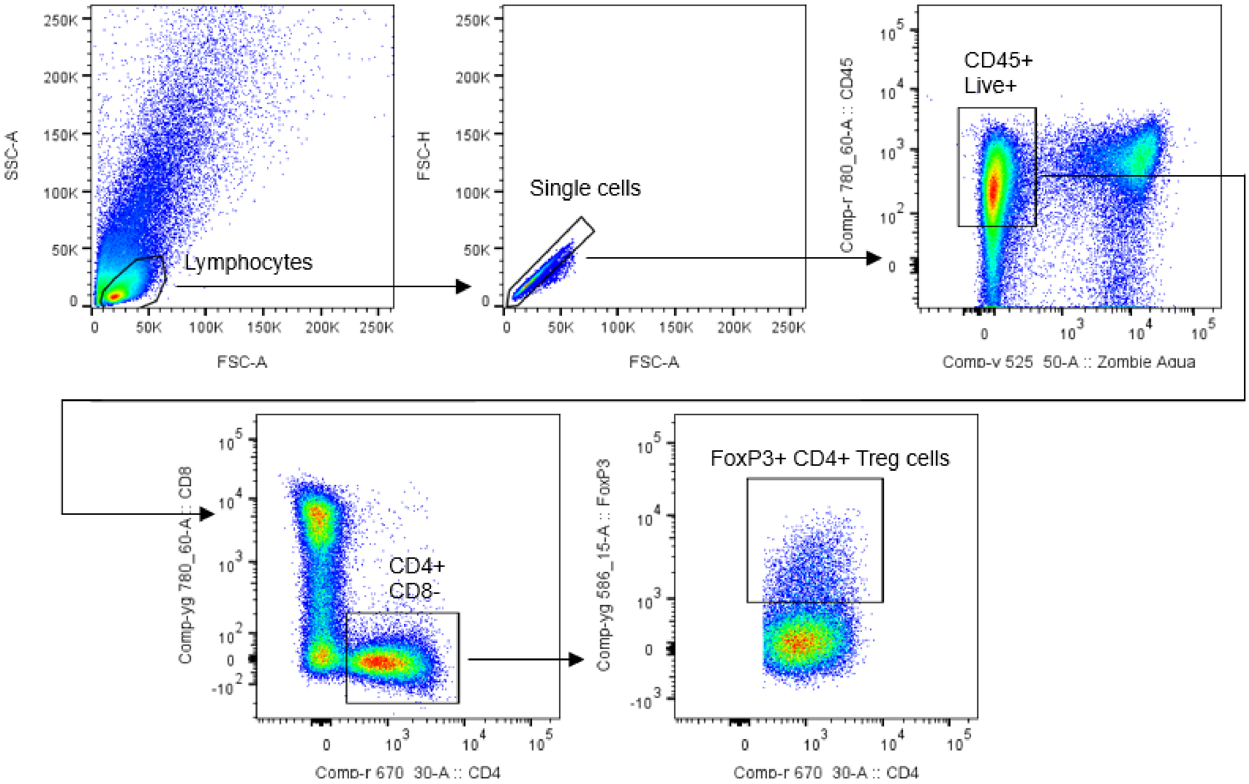

Figure S16: *Gating strategy of human FoxP3[+] CD4[+] Treg from the human PBMC – SKOV3 tumor cell co-culture experiment.*

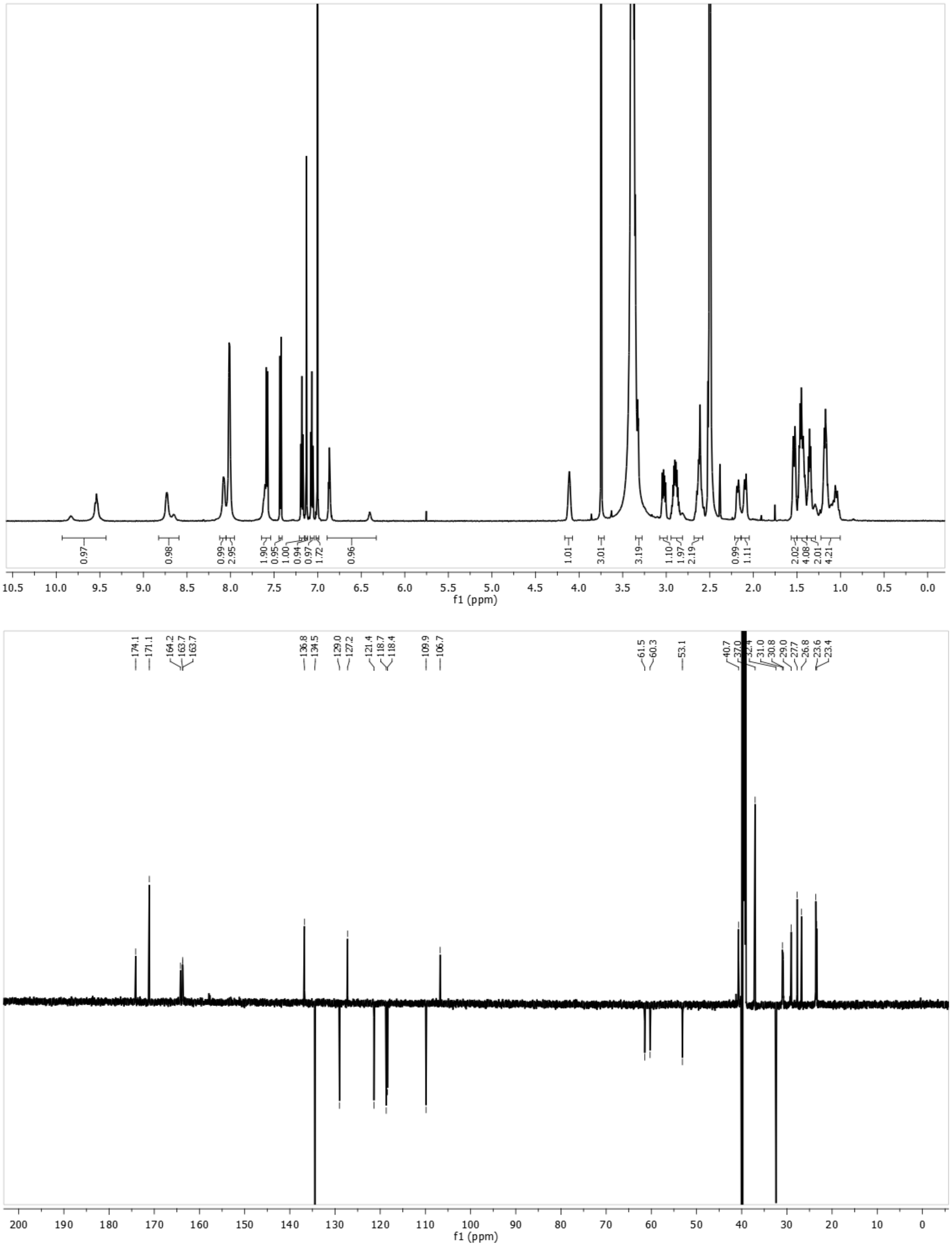

Figure S17: *^{1}H- and ^{13}C-NMR spectra of* ***MalCa/IdoEs*** *in DMSO-d$_6$. The ^{1}H-peaks at ~9.7, ~8.7 and ~6.7 ppm are split due to the different orientations of the carbamate moiety at the platinum core.*

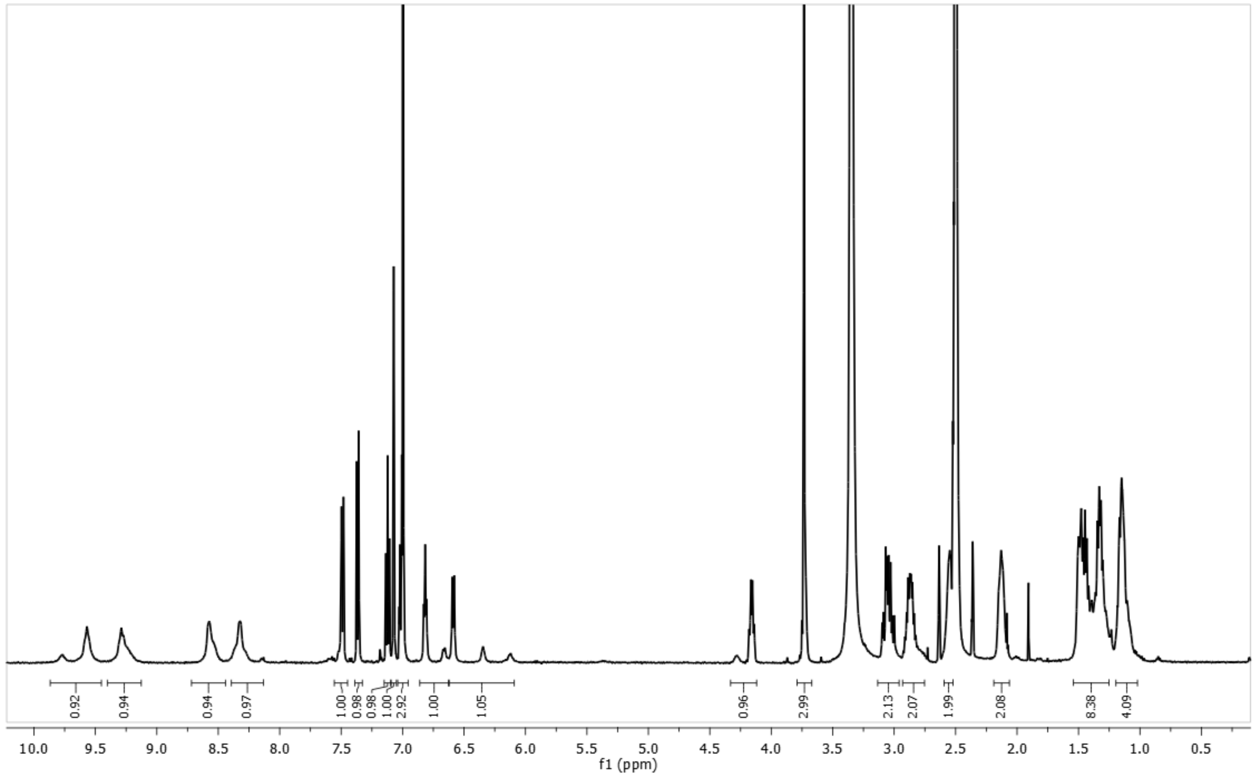

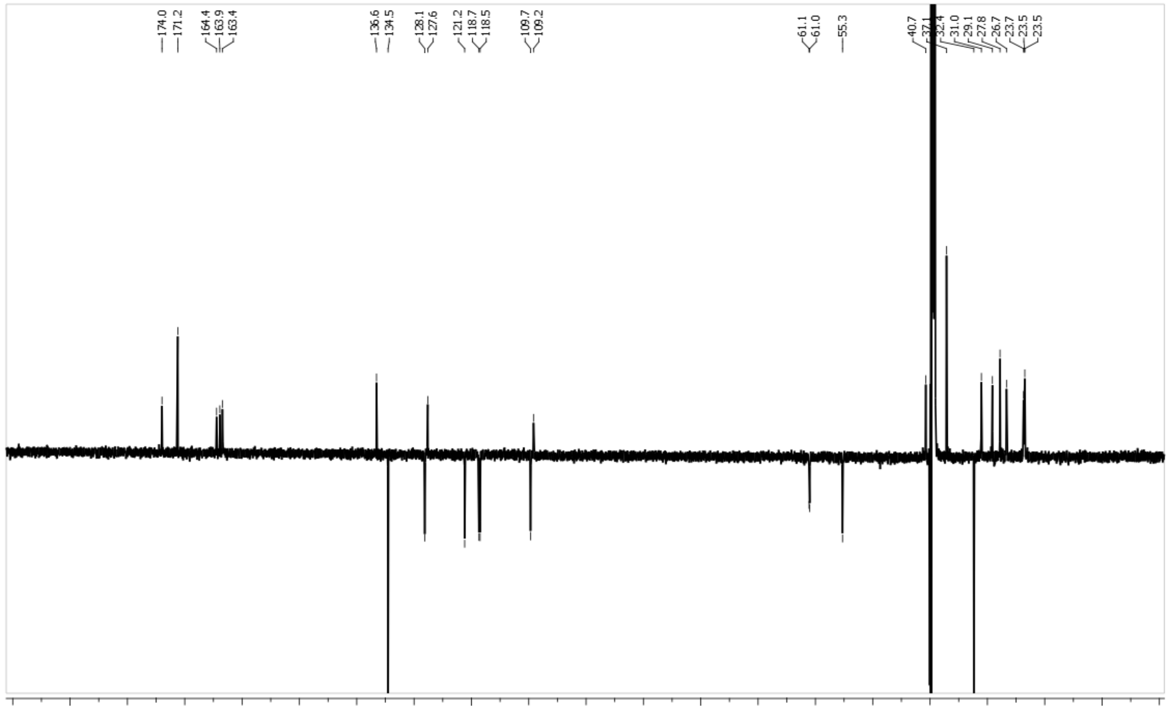

Figure S18: *^{1}H- and ^{13}C-NMR spectra of **MalCa/IdoCa** in DMSO-d_6. The ^{1}H-peaks at ~9.7, ~8.3 and in the range between 6.1 and 6.8 ppm are split due to the different orientations of the carbamate moieties at the platinum core.*

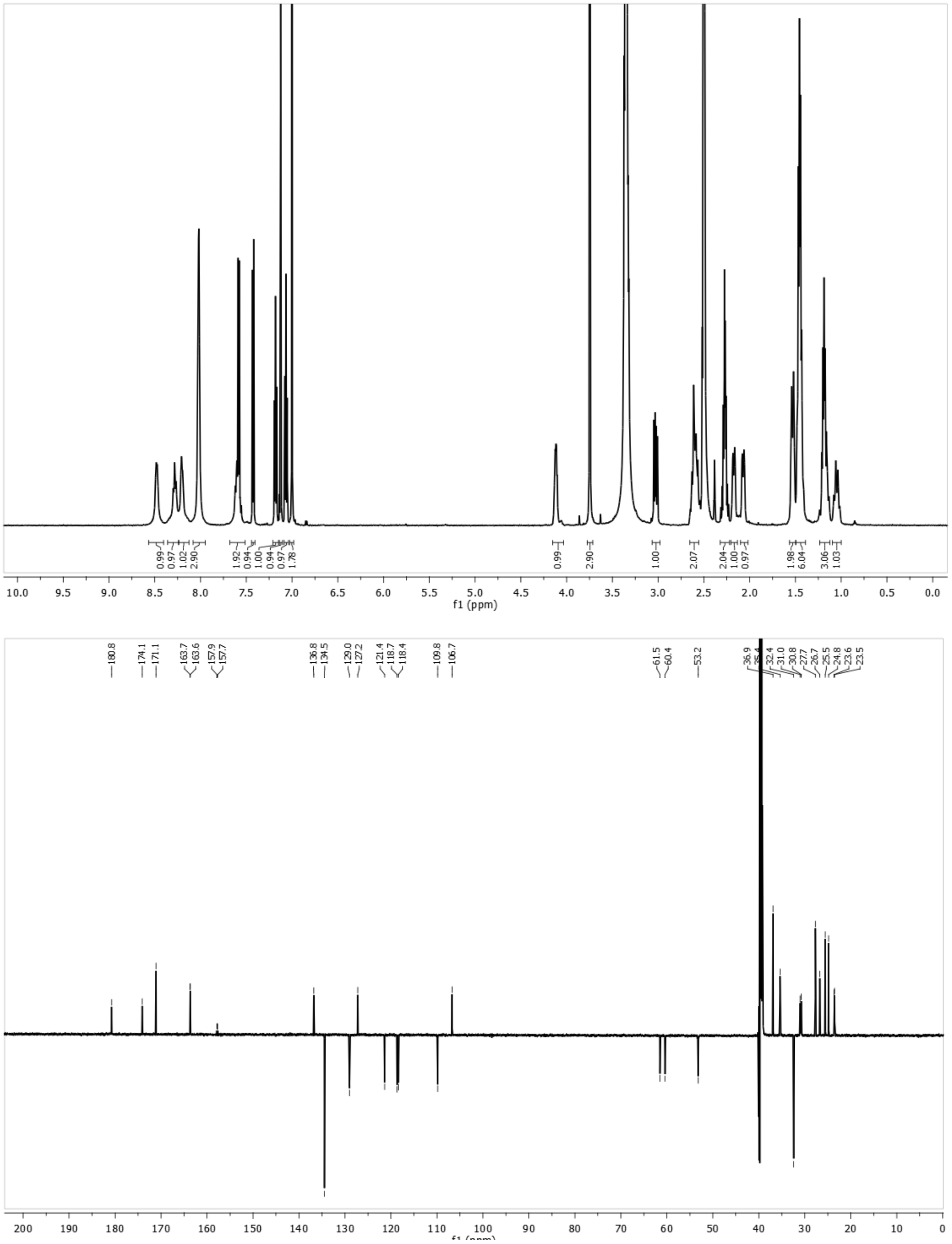

Figure S19: *^{1}H- and ^{13}C-NMR spectra of* ***MalEs/IdoEs*** *in DMSO-d$_6$.*

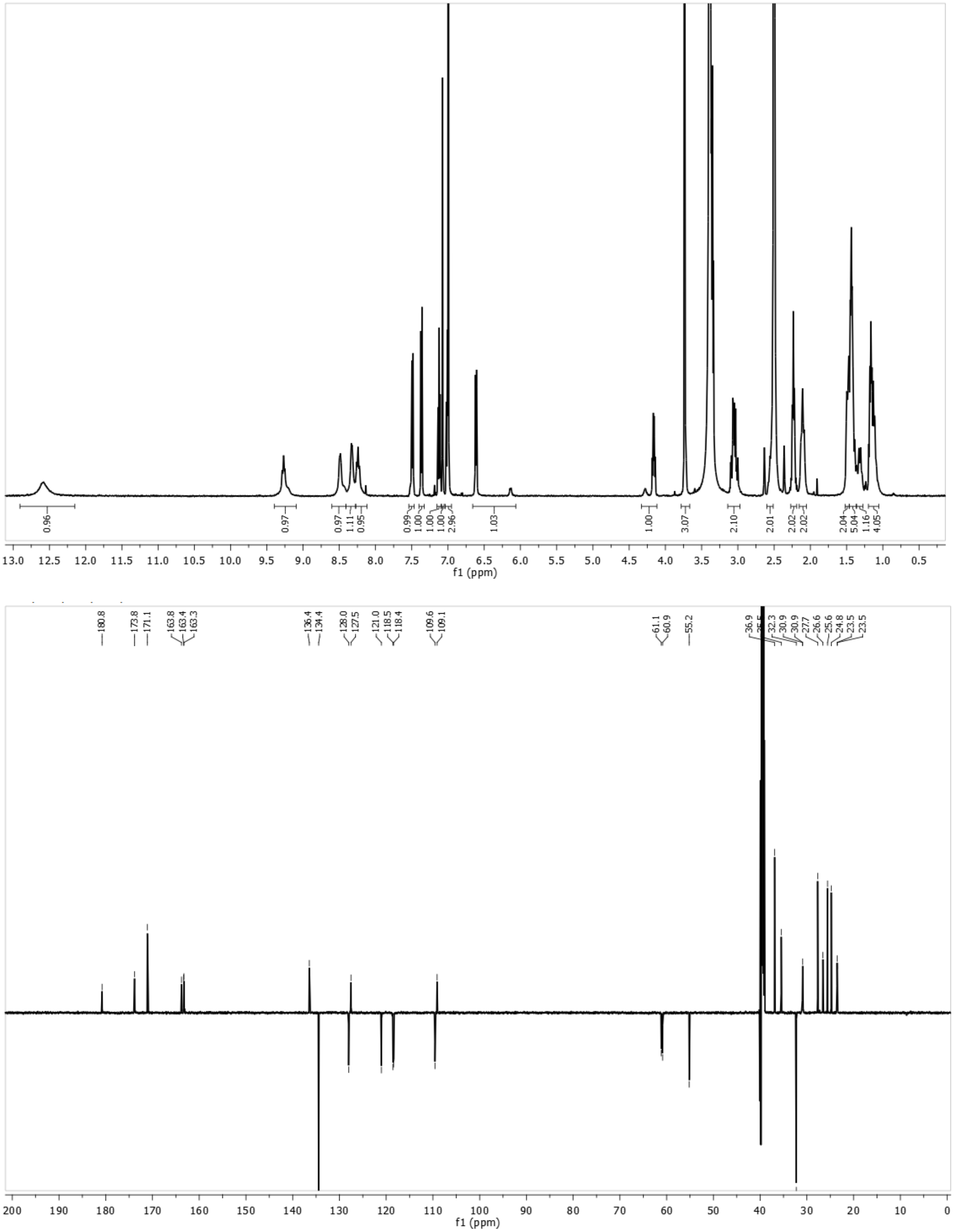

Figure S20: *^{1}H- and ^{13}C-NMR spectra of* ***MalEs/IdoCa*** *in DMSO-d_6. The ^{1}H-peak at ~6.4 ppm is split due to the different orientations of the carbamate moiety at the platinum core.*

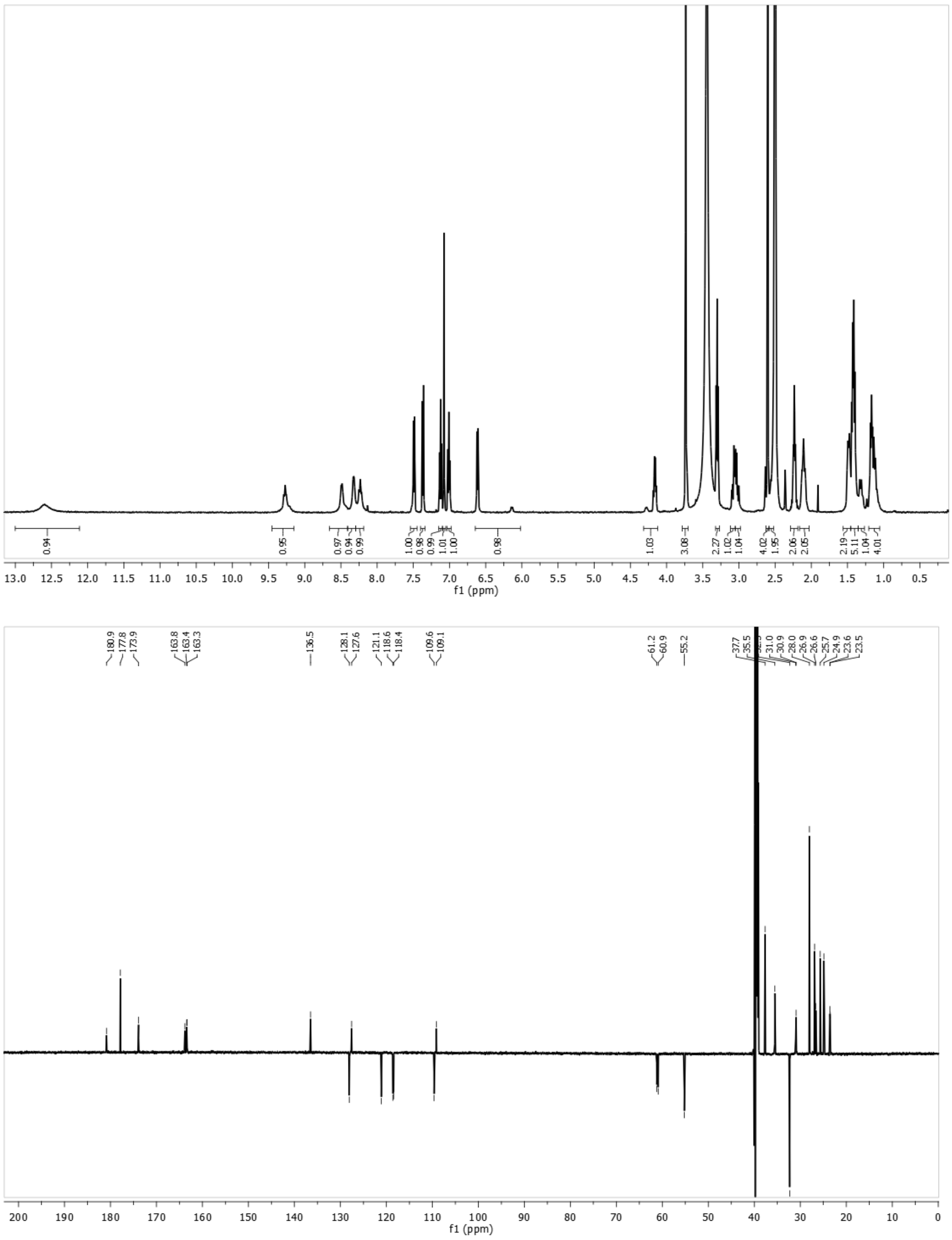

Figure S21: *¹H- and ¹³C-NMR spectra of* ***SucEs/IdoCa*** *in DMSO-d₆. The ¹H-peak at ~6.4 ppm is split due to the different orientations of the carbamate moiety at the platinum core.*

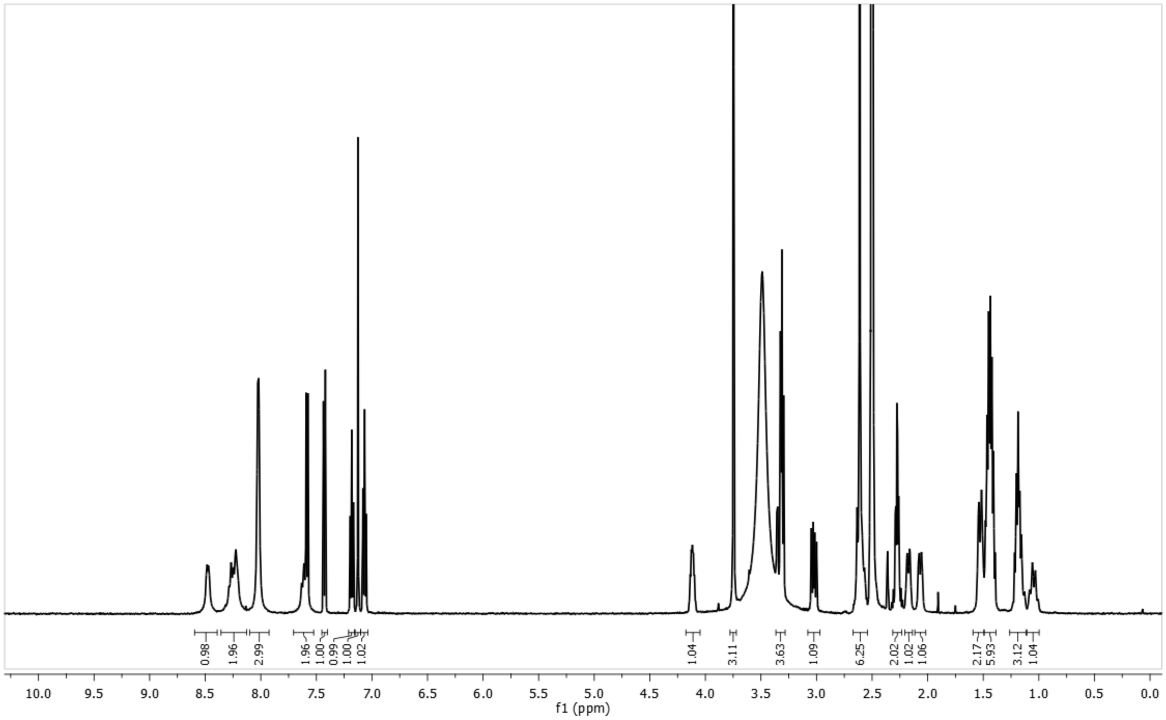

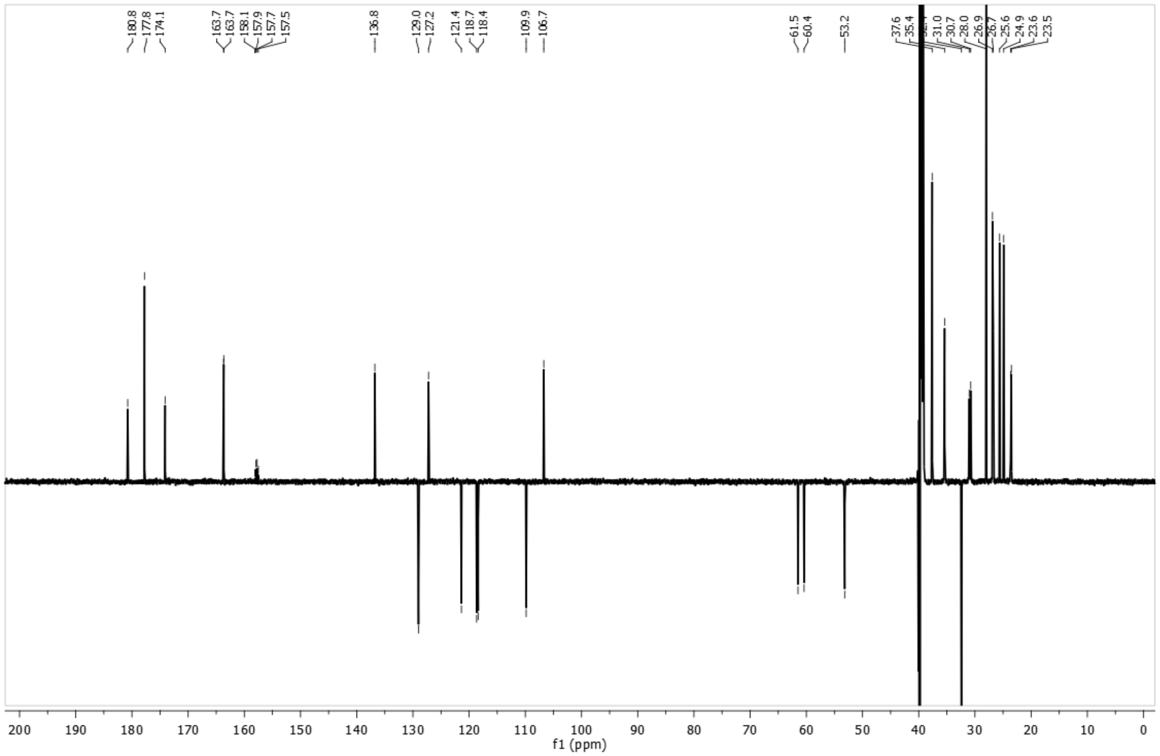

Figure S22: *^{1}H- and ^{13}C-NMR spectra of* ***SucEs/IdoEs*** *in DMSO-d_6.*

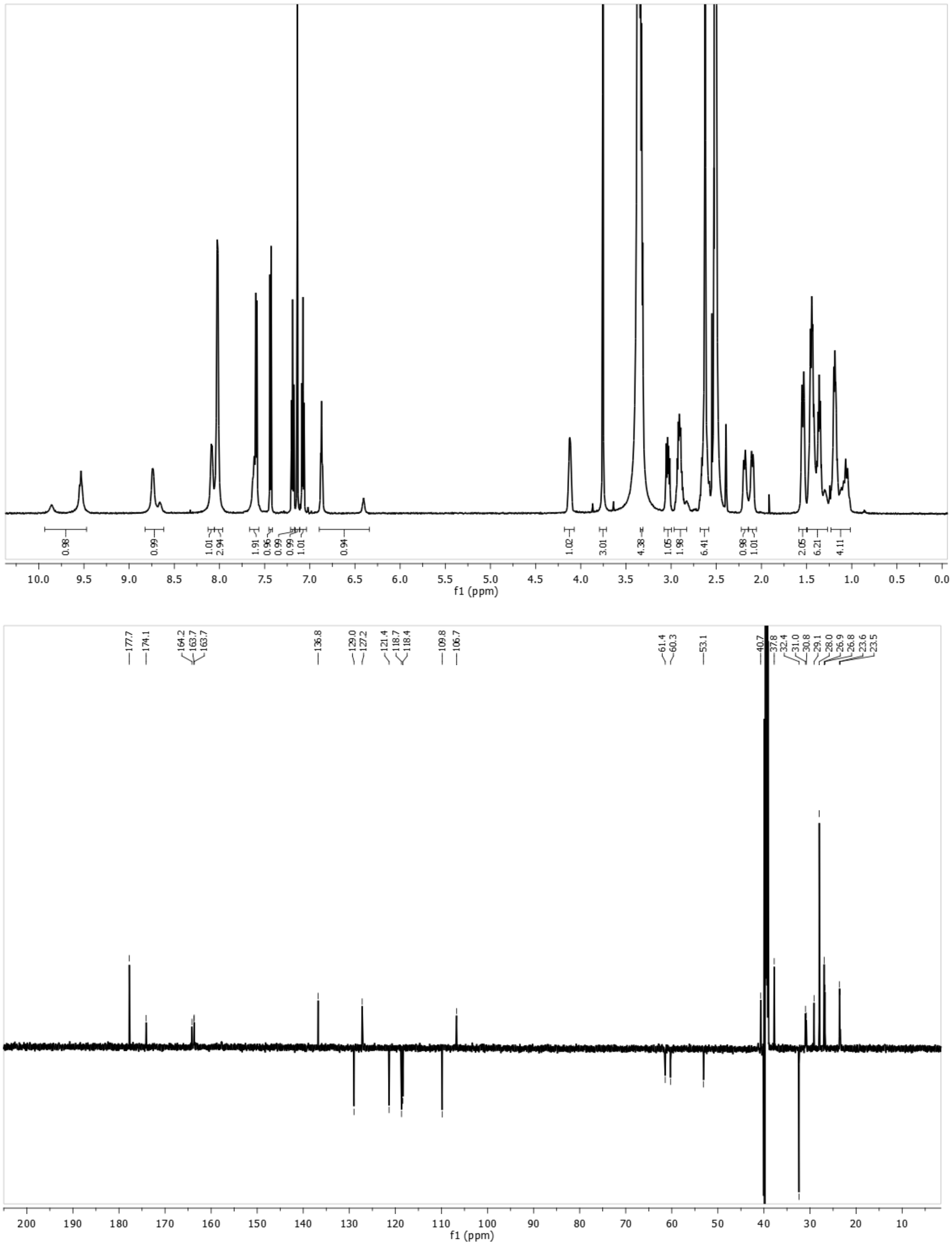

Figure S23: *^{1}H- and ^{13}C-NMR spectra of **SucCa/IdoEs** in DMSO-d₆. The ^{1}H-peaks at ~9.7, ~8.7 and ~6.7 ppm are split due to the different orientations of the carbamate moiety at the platinum core.*

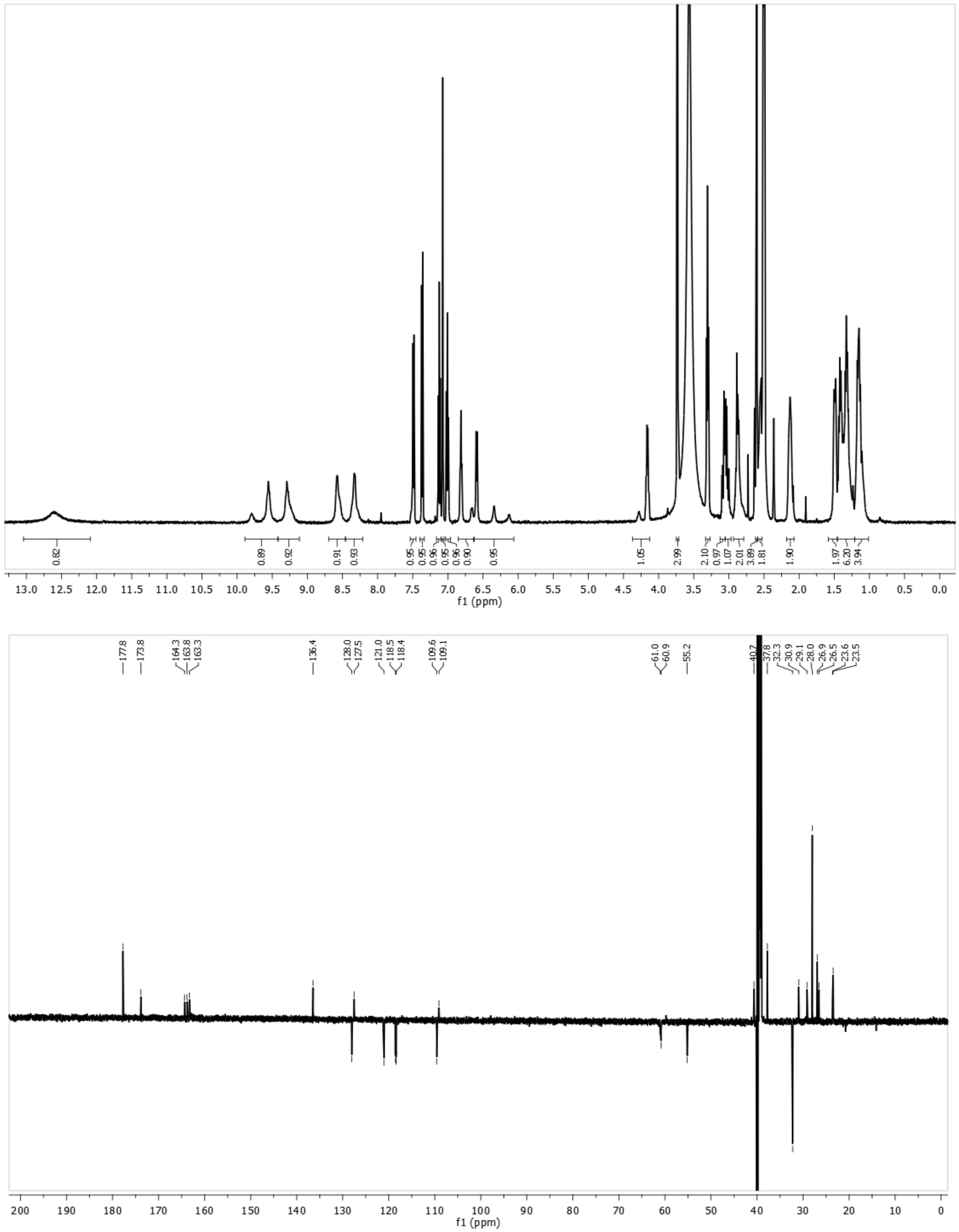

Figure S24: *^{1}H- and ^{13}C-NMR spectra of* ***SucCa/IdoCa*** *in DMSO-d$_6$. The ^{1}H-peaks at ~9.7 and in the range between 6.1 and 6.8 ppm are split due to the different orientations of the carbamate moieties at the platinum core.*

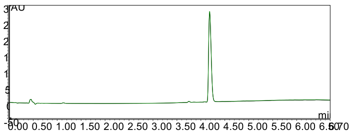

Figure S25: *UHPLC-chromatogram of **MalEs/IdoEs** at 254 nm.*

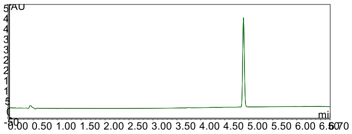

Figure S26: *UHPLC-chromatogram of **MalCa/IdoCa** at 254 nm.*

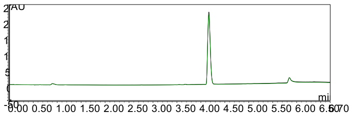

Figure S27: *UHPLC-chromatogram of **MalCa/IdoEs** at 254 nm.*

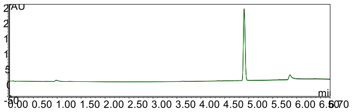

Figure S28: *UHPLC-chromatogram of* ***MalEs/IdoCa*** *at 254 nm.*

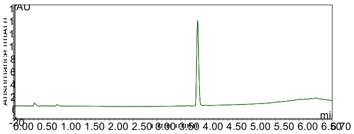

Figure S29: *UHPLC-chromatogram of* ***SucEs/IdoEs*** *at 254 nm.*

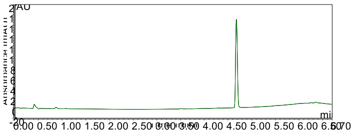

Figure S30: *UHPLC-chromatogram of* ***SucEs/IdoCa*** *at 254 nm.*

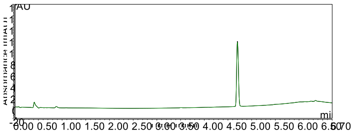

Figure S31: *UHPLC-chromatogram of* ***SucCa/IdoCa*** *at 254 nm.*

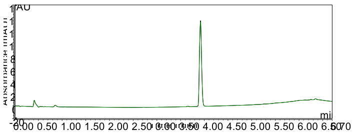

Figure S32: *UHPLC-chromatogram of* ***SucCa/IdoEs*** *at 254 nm.*

Table S1: *Cytotoxicity determined by MTT assay in murine CT26 and human HCT116 colon cancer cells after 72 h incubation with and without 5 eq. AA.*

**CT26 - IC_{50} values (μM) - 72 h**							
		**-**			**+ 5 eq. AA**		
	mean		SD	mean		SD	ratio
oxaliplatin	1.90	±	0.34	2.11	±	0.69	0.90
OAc/OAc	42.34	±	10.99	33.54	±	2.85	1.26
SucCa/OAc	55.17	±	12.64	40.39	±	5.62	1.37
SucEs/IdoCa	90.46	±	3.14	46.03	±	3.86	1.97
SucCa/IdoCa	82.49	±	8.19	43.65	±	6.11	1.89
SucEs/IdoEs	44.94	±	4.81	18.13	±	3.48	2.48
SucCa/IdoEs	31.86	±	6.11	16.94	±	3.21	1.88

**HCT116 - IC_{50} values (μM) - 72 h**							
		**-**			**+ 5 eq. AA**		
	mean		SD	mean		SD	ratio
oxaliplatin	0.76	±	0.16	0.74	±	0.14	1.02
OAc/OAc	14.33	±	2.50	11.50	±	0.92	1.25
SucCa/OAc	42.02	±	7.39	21.79	±	2.38	1.93
SucEs/IdoCa	82.11	±	10.02	20.82	±	2.03	3.94
SucCa/IdoCa		>100		24.74	±	2.49	≥4
SucEs/IdoEs	5.45	±	1.42	4.89	±	1.10	1.12
SucCa/IdoEs	7.23	±	1.72	5.35	±	1.28	1.35

Table S2: *Cytotoxicity determined by MTT assay in human embryonic kidney cells (HEK) and human hepatic cell line (WRL68) after 72 h.*

**IC50 values (µM) - 72 h**						
	**HEK293**			**WRL68**		
	mean		SD	mean		SD
oxaliplatin	2.10	±	0.27	8.35	±	1.23
OAc/OAc	12.21	±	0.48	33.58	±	8.39
SucCa/OAc	50.83	±	10.26		>100	
SucEs/IdoCa	77.49	±	16.10		>100	
SucCa/IdoCa	81.23	±	21.34		>100	
SucEs/IdoEs	44.16	±	8.32	81.69	±	0.40
SucCa/IdoEs	33.19	±	9.03	76.36	±	6.67

Table S3: *Cytotoxicity determined by MTT assay in human SKOV3 and VM7 cancer cells after 72 h incubation with and without 5 eq. AA.*

**SKOV3 - IC_{50} values (µM) - 72 h**							
	**-**			**+ 5 eq. AA**			
	mean		SD	mean		SD	ratio
oxaliplatin	0.76	±	0.16	43.35	±	4.88	1.05
OAc/OAc	14.33	±	2.50				
SucCa/OAc	>100			>100			
SucEs/IdoCa	>100			>100			
SucCa/IdoCa	>100			>100			
SucEs/IdoEs	>100			>100			
SucCa/IdoEs	>100			>100			

**VM7- IC_{50} values (µM) - 72 h**			
	mean		SD
oxaliplatin	3.94	±	0.78
OAc/OAc	43.49	±	3.33
SucCa/OAc	>100		
SucEs/IdoCa	>100		
SucCa/IdoCa	>100		
SucEs/IdoEs	>100		
SucCa/IdoEs	>100		

Table S4: *Ratio of mean platinum level of tissue samples from figure 8.*

	Platinum ratio	
	tumor:liver	tumor:kidney
oxaliplatin	0.3	0.3
OAc/OAc	0.4	0.3
MalEs/IdoCa	0.7	1.2
MalCa/IdoCa	0.5	0.5
MalEs/IdoEs	0.2	0.5
MalCa/IdoEs	0.6	0.6

Table S5: *SEC-HPLC parameters for SEC-ICP-MS measurements.*

HPLC column:	Acquity UPLC BEH 200Å 1.7 µm, 4.6x150 mm
Eluent:	50 mM CH_3COONH_4, pH = 6.8
Flow rate:	400 µL/min
Injection volume:	5 µL
Column temperature:	37°C
Autosampler temperature:	37°C

Table S6: *ICP-MS parameters for SEC-ICP-MS measurements.*

Nebulizer:	Quartz
Spray chamber:	Scott type
Nebulizer gas flow:	1.08 L/min
Aux. gas flow:	0.9 L/min
Plasma gas flow:	15 L/min
Reaction gas (oxygen):	30 %
ICP RF power:	1550 W
m/z measured:	195, 48

Table 7: *ICP-MS parameters for the measurement of platinum levels in HCT116 cells and tumor tissues.*

RF power	1550 W
Nebulizer	MicroMist
Spray chamber	Scott double-pass
Spraying chamber temp.	2°C
Monitored Isotopes	^{185}Re, ^{195}Pt, ^{196}Pt
Measurement modes	standard mode
Plasma gas	15 L min^{-1}
Nebulizer gas	1.08 L min^{-1}
Auxiliary gas	0.90 L min^{-1}
Cones	Ni
Cell entrance	–60 V
Cell exit	–110 V
Integration time	0.1 or 0.3 s

Table S8: *List of antibodies from Biolegend that were used for multi-color flow cytometry experiments.*

**Fluorophore**	**Antigen**	**Reactivity**	**Clone**	**Dilution**
APC/Cyanine 7	CD45	Mouse	30-F11	1:200
FITC	CD3ε	Mouse	145-2C11	1:500
BV 421™	CD8a	Mouse	53-6.7	1:100
PE/Cyanine7	CD4	Mouse	GK1.5	1:200
AF® 647	FOXP3	Mouse	MF-14	1:50
APC/Cyanine 7	CD45	Human	2D1	1:50
FITC	CD3	Human	UCHT1	1:33
PE/Cyanine 7	CD8	Human	SK1	1:66
APC	CD4	Human	A161A1	1:100
PE	FOXP3	Human/Mouse/Rat	150D	1:25

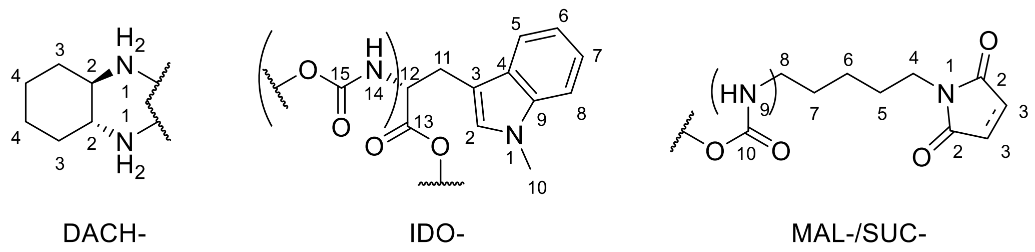

Scheme S1: *NMR numbering scheme for the different ligand systems.*
